# Polycomb scaffolding subunit EED supports the SCLC neuroendocrine identity and *EGFR*-mutant LUAD-to-SCLC transformation

**DOI:** 10.1172/JCI199454

**Published:** 2026-08-18

**Authors:** Yixiang Li, Yasmin N. Laimon, Hyeonseo Cho, Marina Vivero, Gabriel Roberti De Oliveira, Maxwell D. Seager, Andrew Delcea, Varunika Savla, Yuting Chen, Yavuz T. Durmaz, Xintao Qiu, Shweta Kukreja, Rong Li, Talal El Zarif, Wesley Lu, McKayla Van Orden, Jacob E. Berchuck, Roderick T. Bronson, Shuqiang Li, Hongbin Ji, David A. Barbie, Katerina Politi, Matthew L. Freedman, Henry W. Long, Sabina Signoretti, Matthew G. Oser

**Affiliations:** 1 Department of Medical Oncology, Dana-Farber Cancer Institute and Brigham and Women’s Hospital, and; 2 Department of Pathology, Brigham and Women’s Hospital, Harvard Medical School, Boston, Massachusetts, USA.; 3 State Key Laboratory of Cell Biology, Shanghai Institute of Biochemistry and Cell Biology, Center for Excellence in Molecular Cell Science, Chinese Academy of Sciences, Shanghai, China.; 4 Center for Functional Cancer Epigenetics and; 5 Translational Immunogenomics Lab, Dana-Farber Cancer Institute, Boston, Massachusetts, USA.; 6 Winship Cancer Institute, Emory University School of Medicine, Atlanta, Georgia, USA.; 7 Division of Immunology, Department of Microbiology and Immunobiology, Harvard Medical School, Boston, Massachusetts, USA.; 8 University of Chinese Academy of Sciences, Beijing, China.; 9 School of Life Science, Hangzhou Institute for Advanced Study, University of Chinese Academy of Sciences, Hangzhou, China.; 10 School of Life Science and Technology, Shanghai Tech University, Shanghai, China.; 11 Departments of Pathology and Internal Medicine (Section of Medical Oncology), Yale School of Medicine and Yale Cancer Center, New Haven, Connecticut, USA.; 12 Department of Oncologic Pathology, Dana-Farber Cancer Institute, Boston, Massachusetts, USA.; 13 Department of Medicine, Brigham and Women’s Hospital, Harvard Medical School, Boston, Massachusetts, USA.

**Keywords:** Genetics, Oncology, Epigenetics, Lung cancer, Mouse models

## Abstract

Lung cancer histological subtypes include lung adenocarcinoma (LUAD) and small-cell lung cancer (SCLC). Although usually distinct, rare combined LUAD/SCLC tumors occur, and LUAD can transform into SCLC as a mechanism of resistance to targeted therapies, particularly in *EGFR*-mutant LUAD tumors with *RB1/TP53* inactivation. Although PRC2 complex expression increases during this transformation, its functional role remains unclear. Using CRISPR-based autochthonous immunocompetent GEMMs, we found that inactivation of EED, the core PRC2 scaffolding subunit, impaired SCLC tumorigenesis and promoted LUAD histological identity, likely through a NEUROD1-positive intermediate state. Mechanistically, EED loss derepressed bivalent genes comarked by H3K27me3 and H3K4me3, including LUAD oncogenic RAS, PI3K, and MAPK pathway genes and NEUROD1. These same LUAD oncogenic signaling genes were bivalently repressed in human SCLC patient-derived xenografts, suggesting a conserved PRC2-dependent mechanism that represses LUAD oncogenic signaling and thereby supports the SCLC neuroendocrine identity. In a complementary *EGFR*-mutant LUAD GEMM with *Rb1* and *Trp53* inactivation, EED inactivation at tumor initiation prevented the emergence of SCLC histology after EGFR oncogene withdrawal and redirected recurrent tumors toward mucinous LUAD states with reduced spontaneous metastasis. These findings identify PRC2/EED as a regulator of SCLC neuroendocrine identity and nominate pharmacologic EED inhibition for future investigation in therapy-associated LUAD-to-SCLC transformation.

## Abstract 2

This study shows that EED, a scaffolding protein in PRC2, helps small cell lung cancer keep its neuroendocrine identity by repressing lung adenocarcinoma genes.

## Graphical abstract

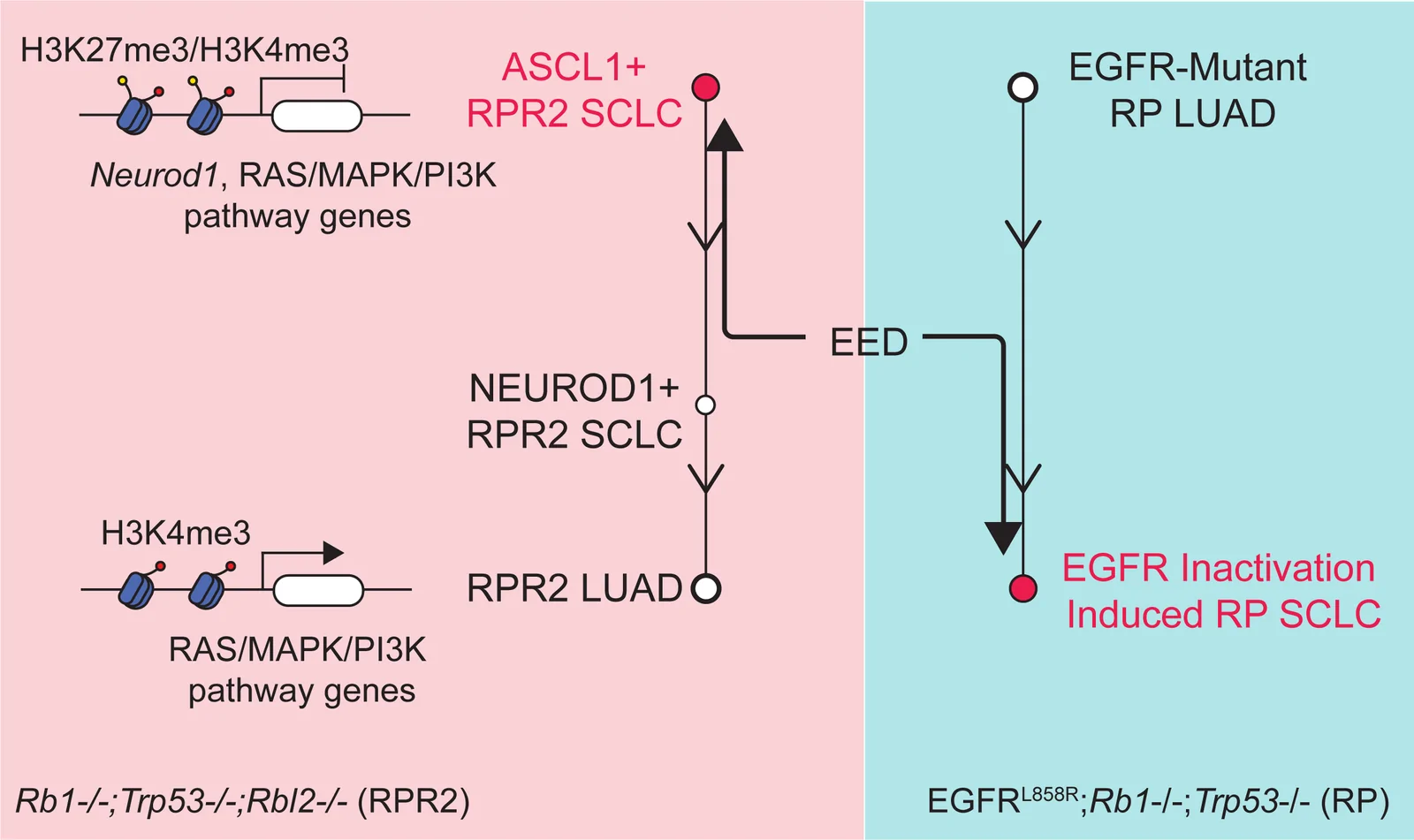

## Introduction

Lung cancer is the leading cause of cancer death worldwide and comprises non–small cell lung cancer (NSCLC), including lung adenocarcinoma (LUAD) and squamous cell carcinoma, as well as small-cell lung cancer (SCLC) (1). Although most lung cancers present with a single histology, rare combined tumors containing LUAD, squamous cell carcinoma, and/or SCLC components are observed (2, 3). Lung cancer histologies can also interconvert through histological transformation, most commonly when *EGFR*-mutant LUAD transforms to SCLC under selective pressure from EGFR inhibition, a clinical scenario associated with poor prognosis and limited treatment options (4–8). SCLC histological transformation has also been observed upon resistance to other targeted therapies in LUAD, and a similar neuroendocrine transformation occurs in prostate adenocarcinoma after androgen deprivation therapy (3, 9).

Human genomic studies have identified recurrent alterations associated with LUAD-to-SCLC transformation, including near-universal *RB1* and *TP53* loss (10–12). Concurrent *RB1* and *TP53* alterations in *EGFR*-mutant LUAD increase SCLC transformation risk by 43-fold (13), and PI3K pathway alterations, including *PIK3CA* mutations and *PTEN* loss, are also associated with SCLC transformation (5, 7, 14). Consistent with these observations, the first *EGFR*-mutant LUAD-to-SCLC Genetically Engineered Mouse Model (GEMM) showed that efficient transformation requires *Rb1* (R) and *Trp53* (P) loss, nondegradable c-Myc overexpression (M), and suppression of the initiating EGFR L858R oncogene, with *Pten* loss further enabling SCLC formation from alveolar type II cells in the RPM model (15). However, the functional and epigenetic mechanisms that constrain lung cancer histological states and drive LUAD-to-SCLC transformation remain poorly understood.

SCLC exists broadly in 4 molecular subtypes: ASCL1, NEUROD1, POU2F3, and inflammatory (16, 17). ASCL1 and NEUROD1 tumors exhibit intratumoral heterogeneity (18) and subtype plasticity, with ASCL1-to-NEUROD1 transitions driven by c-MYC activation (19) and KDM6A loss (20).

EED is the core scaffolding subunit of PRC2, which deposits H3K27me3 to repress target genes (21, 22). EZH2 is highly expressed in SCLC relative to LUAD (23, 24), SCLC transformation is correlated with elevated EZH2 expression and PRC2 activity (5), and EZH2 inhibition suppresses SCLC growth (24). Prior studies also showed that EED inactivation in *KRAS*-driven LUAD promotes mucinous adenocarcinoma differentiation (25, 26). However, whether PRC2 is functionally required for the ASCL1-positive SCLC neuroendocrine phenotype or LUAD-to-SCLC transformation remains unknown. Here, we hypothesized that PRC2 is important for SCLC tumorigenesis and acts to support the SCLC neuroendocrine identity. We focused on EED because it is an obligate PRC2 core component whose inhibition suppresses PRC2 activity independent of potential EZH1 compensation after EZH2 inhibition (27). Using CRISPR-engineered autochthonous immunocompetent GEMMs, we demonstrate that EED supports the SCLC neuroendocrine phenotype and LUAD-to-SCLC transformation.

## Results

### EED inactivation promotes a shift from SCLC toward LUAD histology in an RPR2 GEMM in vivo.

To study the role of EED in SCLC tumorigenesis and test whether PRC2 activity supports the ASCL1-positive neuroendocrine SCLC phenotype in vivo, we used a CRISPR/Cas9-based, autochthonous, immunocompetent RPR2 GEMM of SCLC developed by our group (20, 28–31). In this model, candidate genes are genetically inactivated at tumor initiation together with *Rb1*, *Trp53*, and *Rbl2* (RPR2). Similar to the classical RPR2 GEMM (32), the CRISPR-based RPR2 model gives rise to ASCL1-subtype SCLC that highly and homogeneously expresses ASCL1 with no detectable NEUROD1 expression (20, 28, 29, 31). We generated adenoviruses that encode (a) CMV-Cre to activate Cas9 expression; (b) sgRNAs targeting *Rb1*, *Trp53*, and *Rbl2* (RPR2); and (c) an sgRNA targeting *Eed* or a nontargeting sgRNA as a control (Figure 1A). These adenoviruses simultaneously inactivated all 4 targets and induced Cre expression in vitro (Supplemental Figure 1A; supplemental material available online with this article; https://doi.org/10.1172/JCI199454DS1). We then intratracheally injected these adenoviruses into lox-stop-lox (LSL) Cas9-P2A-GFP mice to generate EED-inactivated RPR2 or EED-WT RPR2 tumors (Figure 1A). All Cas9-positive tumor cells in this model are GFP-positive. We targeted *Eed* rather than *Ezh2* because EED is a core scaffolding protein of the PRC2 complex, thereby avoiding potential EZH1 paralog compensation (33).

Consistent with prior studies nominating EZH2 as a therapeutic target in SCLC (23, 24), EED inactivation significantly delayed tumorigenesis, although tumors eventually formed in most mice injected with EED-inactivated RPR2 adenovirus (Figure 1, B–D). Strikingly, approximately 65% of EED-inactivated RPR2 mice developed tumors with pure LUAD histology and no evidence of SCLC (*n* = 24 of 37), whereas 27% developed independent LUAD and SCLC tumors within the same animal (*n* = 10 of 37), and only 1 mouse developed pure SCLC (Figure 1, C and D, Supplemental Figure 1B, and Supplemental Table 1, tab 1). IHC confirmed that all tumor cells were GFP-positive and had complete loss of EED and the PRC2 functional mark H3K27me3, while only SCLC tumors, and not LUAD tumors, expressed the neuroendocrine marker synaptophysin (Figure 1E, Supplemental Figure 1C, and Supplemental Table 1, tab 2). Consistent with prior work (20), EED-WT RPR2 SCLC tumors highly and homogeneously expressed ASCL1 and lacked NEUROD1 expression, whereas EED-inactivated RPR2 LUAD tumors lost ASCL1 expression (Figure 1E). CRISPR amplicon sequencing confirmed indels in *Eed* and *Rb1* in select tumors (Supplemental Figure 1, D and E). Whereas most EED-WT RPR2 SCLC tumors metastasized to the liver, consistent with prior RPR2 studies (29, 34) and the highly metastatic nature of SCLC (1), EED-inactivated RPR2 LUAD tumors were confined to the lung without evidence of distant metastasis (Figure 1F). Together, these findings demonstrate strong selective pressure for a phenotypic shift from SCLC to LUAD following EED loss. However, because EED was inactivated at tumor initiation and tumors were analyzed at endpoint, these data do not distinguish when this selection toward LUAD occurred during tumorigenesis.

Given these findings, together with prior studies showing that SCLC transformation is associated with high EZH2 expression and increased PRC2 activity–associated gene signatures (5), we next asked whether PRC2 complex expression in SCLC is unique among epigenetic regulators. We analyzed publicly available RNA-seq data from 2 independent cohorts comparing transformed SCLC with pretransformed LUAD (5, 35) across 120 epigenetic modifiers (Figure 1G, Supplemental Figure 1F, and Supplemental Table 1, tab 3). Only 10 epigenetic modifiers were consistently upregulated in SCLC across both datasets, with EZH2 emerging as the most highly differentially expressed epigenetic regulator (Figure 1H and Supplemental Figure 1, G and H). Together, these data support a model in which PRC2 activity acts to constrain the SCLC neuroendocrine phenotype, with its loss promoting a histology shift toward LUAD.

### EED inactivation Epigenetically Restores LUAD Oncogenic Signatures In Vivo.

To investigate the molecular consequences of EED loss in the RPR2 GEMM, we performed RNA-seq on primary EED-inactivated RPR2 LUAD tumors and EED-WT RPR2 SCLC tumors (Supplemental Table 2, tab 1). Gene set enrichment using MSigDB (35), ENCODE (36), and ChEA (37) showed that genes upregulated in EED-inactivated tumors were enriched for targets normally bound and repressed by PRC2 members, including EZH2 and SUZ12 (Figure 2, A and B), consistent with functional PRC2 loss. EED-inactivated RPR2 LUAD tumors lost canonical neuroendocrine markers, including *Chga*, *Insm1*, and *Syp* (Figure 2C), and gained expression of RTK, KRAS, innate immune, and antigen processing and presentation pathways (Figure 2D). Flow cytometry confirmed restored MHC class I antigen presentation and increased immune and stromal infiltration (Supplemental Figure 2, A–D). Transcriptomic analysis showed that EED-inactivated RPR2 LUAD tumors correlated with pure and pretransformed human LUAD tumors, whereas EED-WT RPR2 SCLC tumors correlated with de novo and transformed SCLC tumors (Supplemental Figure 2E and Supplemental Table 2, tab 5), supporting the relevance of this model to human lung cancer (5).

Histologic analysis revealed that EED-inactivated LUAD tumors displayed mucinous differentiation (Supplemental Figure 2F), a feature observed in subsets of human LUAD associated with *KRAS* mutations, *NKX2-1* loss, and gastrointestinal lineage gene expression (38–41). Correspondingly, EED-inactivated LUAD tumors upregulated gastrointestinal lineage markers including *Cdx2*, *Hnf4a*, and *Pdx1*, were enriched for gastrointestinal development signatures (Figure 2E and Supplemental Figure 2G), exhibited features of mucinous LUAD tumors (Supplemental Figure 2H), and lost NKX2-1 protein expression (Supplemental Figure 2I).

To define the chromatin-level changes underlying this reprogramming, we performed ATAC-seq (Assay for Transposase-Accessible Chromatin using sequencing) and H3K27ac ChIP-seq on EED-inactivated LUAD tumors and EED-WT SCLC tumors (Figure 2, F–I, Supplemental Figure 2, J and K, and Supplemental Table 2, tabs 3 and 4). Integrated analysis revealed that approximately 80% of upregulated genes gained both chromatin accessibility and H3K27 acetylation (Figure 2J), indicating widespread epigenetic activation. These included gastrointestinal differentiation factors *Hnf4a* and *Cdx2* and the LUAD oncogene *Met* (Figure 2, K and L). HNF4A motifs were also enriched in regions with increased chromatin accessibility and H3K27ac in EED-inactivated RPR2 LUAD tumors (Figure 2, M and N). Together, these findings indicate that EED loss ultimately promotes LUAD histology in vivo associated with epigenetic activation of LUAD oncogenic programs and gastrointestinal differentiation factors (Figure 2O).

### EED inactivation promotes rare NEUROD1-positive SCLC with early features of LUAD oncogenic signaling.

Although EED-inactivated RPR2 mice predominantly developed LUAD tumors, a small subset formed tumors with SCLC histology (Figure 1C). These rare EED-inactivated RPR2 SCLC tumors showed complete loss of EED by CRISPR amplicon sequencing and IHC as well as complete loss of H3K27me3 by IHC (Supplemental Figure 1, C and D). Compared with EED-WT RPR2 SCLC tumors, EED-inactivated RPR2 SCLC tumors exhibited distinct morphology with giant cells and enlarged nuclei and were transcriptionally enriched for EMT, inflammatory response, and pancreatic β cell signatures (Figure 3, A–C, and Supplemental Table 3, tabs 1 and 2). A defining marker of the pancreatic β cell signature was *Neurod1*, which marks the NEUROD1-high SCLC-N subtype of human SCLC (11, 16, 17). These tumors showed high *Neurod1* and NEUROD1-correlated gene expression with reduced *Ascl1* and ASCL1-correlated gene expression (Figure 3, D and E), suggesting that EED inactivation promotes ASCL1-to-NEUROD1 subtype switching in vivo. FPKM values for key neuroendocrine and LUAD genes are shown in Supplemental Table 3, tab 3.

ATAC-seq and H3K27ac ChIP-seq similarly showed that NEUROD1/NEUROG3 and ISL1/2 motifs were among the top motifs with increased chromatin accessibility in EED-inactivated RPR2 SCLC tumors (Supplemental Figure 3, A–I), with *Neurod1* and *Isl1* gaining both chromatin accessibility and H3K27ac (Figure 3, F–H, and Supplemental Table 3, tabs 4 and 5). LUAD-associated RTK signaling genes, including *Met* and *Fgfr1*, were also epigenetically activated in EED-inactivated RPR2 SCLC tumors relative to EED-WT RPR2 SCLC tumors (Figure 3, F–H). Genetic EED inactivation in the ASCL1-positive murine SCLC cell line 1014 increased *Neurod1*, *Met*, and *Fgfr1* mRNA expression, as well as NEUROD1 protein levels (Figure 3, I and J, and Supplemental Table 3, tab 6). Similarly, acute treatment with clinical-grade EZH1/2 or EED inhibitors increased NEUROD1 protein expression in vitro (Supplemental Figure 3J). In contrast to EED-inactivated RPR2 SCLC tumors in vivo, genetic or pharmacologic EED inactivation did not reduce ASCL1 protein expression in 1014 cells in vitro (Figure 3I and Supplemental Figure 3J), suggesting that ASCL1 reduction in vivo is indirect and requires tumor microenvironmental influences and/or evolutionary selection. RNA-seq also identified an EED-inactivated RPR2 tumor positive for POU2F3, which marks a non-neuroendocrine SCLC subtype (Supplemental Figure 4, A–C) (17). However, EED inactivation in 1014 cells did not induce POU2F3 expression (Supplemental Figure 4D), suggesting that emergence of POU2F3-positive SCLC after EED loss is likely indirect.

Finally, ChIP-seq for EED and H3K27me3 identified 6,072 direct EED target genes, 289 of which were epigenetically activated and expressed in EED-inactivated RPR2 SCLC tumors (Figure 3, K and L, Supplemental Figure 3K, and Supplemental Table 4, tabs 1 and 2), including *Neurod1*, *Isl1*, and *Pdx1* (Figure 3M and Supplemental Figure 3L). EED and H3K27me3 were highly enriched at the *Neurod1* locus in EED-WT RPR2 SCLC tumors, without corresponding H3K27ac enrichment or chromatin accessibility, consistent with direct PRC2-mediated repression. In contrast, EED and H3K27me3 were not strongly enriched at the *Ascl1* locus, which showed prominent H3K27ac enrichment and chromatin accessibility, consistent with active gene expression (Supplemental Figure 3, M and N). Together, these findings demonstrate that EED directly represses NEUROD1 and that EED inactivation derepresses NEUROD1, promoting rare NEUROD1-positive SCLC tumors with partial activation of LUAD-associated genes.

### EED Inactivation Promotes LUAD Histology Likely Through a NEUROD1-Positive Intermediate State.

Although EED loss promoted NEUROD1 expression in vitro and generated occasional NEUROD1-positive SCLC tumors in vivo, most tumors in EED-inactivated RPR2 mice were LUAD tumors (Figure 1, C and D), suggesting that sustained SCLC histology is selected against and/or that LUAD fate is favored after EED loss. In line with this possibility, histological examination of LUAD-bearing lungs from EED-inactivated RPR2 mice revealed rare early neuroendocrine-positive lesions expressing SCLC subtype transcription factors, including ASCL1, NEUROD1, and POU2F3 (Supplemental Figure 4E and Supplemental Table 5, tab 1). To better define the relationship of NEUROD1 to lung cancer histological subtypes formed after EED inactivation in vivo, we performed IHC for NEUROD1 and ASCL1 (Figure 4A). EED-inactivated RPR2 SCLC tumors expressed NEUROD1, whereas EED-WT RPR2 SCLC tumors expressed ASCL1 (Figure 4A). Surprisingly, EED-inactivated RPR2 tumors with pure LUAD histology retained sporadic NEUROD1-positive cells intermixed within tumors (Figure 4, A and B). This was unique to EED-inactivated RPR2 LUAD tumors, as NEUROD1-positive cells were not detected in a de novo LUAD from an EGFR-mutant RPR2 GEMM or in EED-WT RPR2 SCLC tumors (Figure 4, A and B, and Supplemental Table 5, tab 2). *Neurod1* mRNA was also elevated in EED-inactivated RPR2 LUAD tumors relative to EED-WT RPR2 SCLC tumors (Figure 4C), suggesting that EED-inactivated RPR2 LUAD tumors may have evolved from NEUROD1-positive SCLC cells.

To investigate this possibility, we performed single-nucleus RNA-seq (snRNA-seq) on 3 independent EED-inactivated RPR2 LUAD tumors. Unsupervised clustering identified 4 tumor cell clusters (Figure 4, D and E). Cluster 3 expressed neuroendocrine markers, including *Neurod1*, *Chga*, and *Ncam1*, and represented approximately 4% of tumor cells, whereas other clusters expressed LUAD genes such as *Met* (Figure 4F). InferCNV revealed distinct CNV landscapes across tumors (Supplemental Figure 5, A–C), but *Neurod1*-positive cluster 3 cells shared copy number alterations with other tumor clusters from the same sample, suggesting derivation from a common clone (Supplemental Figure 5, A–C) (42). CNV-based phylogenetic reconstruction showed that *Neurod1*-positive cluster 3 cells had fewer CNV events than other tumor clusters and could represent an ancestral population from which other clusters arose (Figure 4G and Supplemental Figure 5, D–F). Pseudotime analysis similarly showed early expression of neuroendocrine genes, followed by persistent *Neurod1* and *Ncam1* expression, eventual loss of neuroendocrine genes, and upregulation of LUAD and gastrointestinal differentiation genes, including *Met*, *Hnf4a*, and *Nr1h4* (Figure 4H and Supplemental Figure 5, G and H). EMT genes, including *Cdh6*, *Clu*, and *Itga2*, were also elevated during the persistent *Neurod1*- and *Ncam1*-positive intermediate state.

To validate this trajectory, we performed scRNA-seq on EED-inactivated RPR2 LUAD tumors (*n* = 4) and SCLC tumors (*n* = 2) and compared them with published scRNA-seq data from EED-WT RPR2 SCLC tumors (*n* = 3) (Figure 5A and Supplemental Figure 6A) (20). As expected, EED-inactivated SCLC tumors expressed *Neurod1, Isl1*, and other neuroendocrine markers, whereas EED-inactivated LUAD tumors lost neuroendocrine features and expressed *Hnf4a* and *Muc13* (Supplemental Figure 6B). Unsupervised ordering predicted a single trajectory from EED-WT RPR2 SCLC (*Ascl1*-positive) to EED-inactivated RPR2 SCLC (*Neurod1*-positive) to EED-inactivated RPR2 LUAD (*Hnf4a*-positive), supporting *Neurod1*-positive SCLC as an intermediate state before LUAD formation (Supplemental Figure 6, C–E).

### LUAD histology after EED inactivation requires cues from the tumor immune microenvironment.

Despite upregulating *Neurod1* and *Met*, EED inactivation in 1014 cells in vitro did not induce LUAD lineage markers or a full LUAD phenotypic shift, suggesting that additional tumor microenvironmental factors and/or evolutionary selection are required for the complete shift to LUAD in vivo (Figure 3J). To test this, we transplanted EED-isogenic 1014 cells into immunocompetent BL6J and immunodeficient NCr nude mice (Figure 5B). In immunodeficient hosts, EED-inactivated tumors retained high NEUROD1 and failed to activate MAPK signaling. In contrast, EED-inactivated tumors in immunocompetent mice had lower NEUROD1 and increased MAPK signaling and *Met*, suggesting that the tumor immune microenvironment promotes LUAD-associated signaling after EED loss (Figure 5, C and D, and Supplemental Figure 7A). However, EED-inactivated 1014 tumors did not fully acquire LUAD histology or induce HNF4A (Supplemental Figure 7, A–D), likely reflecting differences between the transplantation model and the autochthonous GEMM. In the autochthonous GEMM, EED was inactivated at tumor initiation in individual lung cells, so selection against EED loss manifested as delayed tumorigenesis and eventual escape as LUAD. In contrast, in the transplantation model, EED was CRISPR-inactivated in a preestablished polyclonal population of 1014 SCLC cells before implantation, allowing cells retaining intact EED through incomplete editing or in-frame edits to outgrow. Consistent with this, endpoint tumors showed variable restoration of EED protein across tumor cells, suggesting that EED loss was selected against, particularly in immunocompetent hosts (Figure 5C, Supplemental Figure 7B, and Supplemental Table 5, tab 3).

To identify immune-derived cues that might promote LUAD histology, we analyzed the tumor immune microenvironment (Figure 5A). EED-WT RPR2 SCLC tumors contained relatively few immune cells, whereas EED-inactivated RPR2 LUAD tumors showed increased neutrophil and macrophage infiltration, and EED-inactivated RPR2 SCLC tumors showed increased macrophages (Figure 5E). M2-like macrophages expressing *Msr1*, *Arg1*, and *Tgfb1* were enriched in both EED-inactivated LUAD and SCLC tumors (Figure 5, F and G, Supplemental Figure 6, F and G, and Supplemental Table 5, tab 4). Cell–cell interaction analysis comparing TIME ligands and tumor cell receptors in EED-inactivated LUAD tumors versus EED-WT SCLC tumors identified 58 enriched receptor-ligand pairs (Figure 5, H and I) (43). Cross-referencing with EED-inactivated 1014 RNA-seq (Figure 3I) identified 14 upregulated tumor-expressed receptors with corresponding TIME ligands, including LUAD RTK oncogenes *Met*, *Ret*, and *Fgfr2*, all converging on RAS signaling (Figure 5, H and I, and Supplemental Table 5, tab 5). Notably, *Met* was the second-highest expressed receptor, with its ligand *Hgf* highly expressed by macrophages in EED-inactivated RPR2 LUAD tumors. Together, these findings suggest that NEUROD1-positive tumor cells represent a transitional state toward LUAD following EED loss and that the tumor immune microenvironment promotes LUAD RTK oncogenic signaling to complete this process in vivo (Figure 5J).

### EED Directly Binds and Represses RAS, PI3K, and MAPK Pathway LUAD Oncogenic Signaling Genes.

To mechanistically understand how EED maintains the ASCL1-positive SCLC neuroendocrine state and why its loss promotes LUAD histology, we performed ChIP-seq for EED and H3K27me3, identifying 6,072 direct EED target genes (Figure 3K). Of these, 831 were epigenetically activated and expressed in EED-inactivated RPR2 LUAD tumors (Figure 6A and Supplemental Table 4, tab 3), including LUAD oncogenes such as *Met* and gastrointestinal lineage genes including *Hnf4a* and *Cdx2* (Figure 6B). Activated EED targets were enriched for EMT, gastric cancer signatures, and RAS, PI3K, and MAPK signaling (Figure 6C and Supplemental Figure 8A). Consistent with these epigenomic data, immunoblot and IHC analyses demonstrated elevated phospho-ERK and phospho-AKT, providing protein-level evidence of MAPK and PI3K pathway activation in EED-inactivated RPR2 LUAD tumors (Figure 6D and Supplemental Figure 8, B and C). In contrast, EED-inactivated SCLC tumors showed increased phospho-AKT, but not phospho-ERK, consistent with an intermediate state. Only 151 EED target genes were commonly activated in both EED-inactivated RPR2 LUAD and SCLC tumors (Figure 6E), yet this shared set was also enriched for RAS and PI3K/AKT pathway genes (Figure 6F and Supplemental Figure 8D), including the RAS guanine nucleotide exchange factor Rasgrp1 (Figure 6G). Whole-exome sequencing of 6 EED-inactivated RPR2 LUAD tumors did not reveal recurrent copy number alterations or somatic mutations in canonical LUAD oncogenic drivers, with only 1 tumor harboring KRAS G12D and another *Kras* amplification (Supplemental Figure 9A). Together, these data support a dominant epigenetic mechanism driving RAS/PI3K/MAPK activation after EED loss.

RAS pathway activation is a conserved hallmark of oncogenic signaling downstream of nearly all oncogenic RTKs in LUAD and antagonizes the neuroendocrine phenotype in murine SCLC (44), a finding we validated in human ASCL1-positive SCLC cell lines overexpressing WT or mutant KRAS G12C (Supplemental Figure 9, B and C). To test whether EED loss creates RAS pathway dependence, we derived 5 cell lines from EED-inactivated RPR2 LUAD tumors — 1350, 1339, 1343, 1344, and 1345 — which grew as adherent monolayers, similar to LUAD lines and unlike SCLC lines (Supplemental Figure 9, D and E). These lines exhibited elevated phospho-ERK and were highly sensitive to the pan-RAS inhibitor daraxonrasib (RMC-6236) (45) and MEK inhibitor trametinib relative to EED-WT RPR2 SCLC cells (Figure 6, H and I, and Supplemental Figure 9, F and G). Consistent with a direct on-target effect, reexpression of WT EED, but not a catalytically inactive EED mutant, in 2 EED-inactivated LUAD lines downregulated MAPK and RAP1 signaling pathways and PRC2 target genes (Supplemental Figure 10, A–G, and Supplemental Table 4, tabs 4 and 5). However, EED reexpression was insufficient to restore neuroendocrine gene expression (Supplemental Table 4, tabs 4 and 5), consistent with EED loss initiating a shift toward LUAD histology but requiring additional tumor microenvironmental cues to complete this process (Figure 5). Together, these data demonstrate that EED directly represses RAS signaling to maintain the SCLC neuroendocrine phenotype and that EED loss induces RAS oncogenic signaling to promote LUAD.

### LUAD histology after PRC2 loss is associated with reactivation of bivalently marked RAS/PI3K/MAPK Pathway Genes and NEUROD1.

Although 6,072 genes were marked by both EED and H3K27me3, relatively few were activated upon EED loss: 289 in SCLC and 831 in LUAD (Figure 3L and Figure 6A). Because bivalent genes, marked by both activating H3K4me3 and repressive H3K27me3, are selectively derepressed upon PRC2 inhibition during differentiation (46), we hypothesized that bivalent genes would be preferentially activated after EED loss and include regulators of SCLC-to-LUAD histological transformation (Figure 7A). To identify bivalent genes, we performed H3K4me3 ChIP-seq in EED-WT RPR2 SCLC tumors and overlaid these peaks with H3K27me3 peaks from the same samples, identifying 7,622 bivalent promoters (Figure 7B, Supplemental Figure 11, A and B, and Supplemental Table 6, tab 1). Consistent with prior studies (47, 48), GREAT and hallmark enrichment analyses showed that bivalent genes were enriched for MHC class I antigen presentation and EMT (Supplemental Figure 11, C–E).

Consistent with our hypothesis, 417 of 831 genes activated in LUAD (~50%) and 118 of 289 genes activated upon EED loss in SCLC (41%) had bivalent promoters (Figure 7, C and E, and Supplemental Table 6, tab 2). Bivalent EED direct targets reactivated in both EED-inactivated SCLC and LUAD had higher expression than nonbivalent reactivated targets (Figure 7, D and F), consistent with bivalency poising genes for activation upon H3K27me3 loss (46). Bivalent genes activated in LUAD tumors after EED loss were enriched for PI3K/AKT and MAPK signaling, including *Akt1*, *Mapkapk3*, and upstream growth factor receptors such as *Erbb2*, with *Akt1* also induced in EED-inactivated RPR2 SCLC (Figure 7, G–J). These findings indicate that EED directly represses bivalent RAS/PI3K/MAPK pathway genes poised for activation upon PRC2 loss.

*Neurod1* was also a bivalent target reactivated in EED-inactivated RPR2 SCLC and among the most highly expressed genes (Figure 7, E, F, and K). In contrast, some reactivated EED direct targets, including gastrointestinal lineage genes *Hnf4a*, *Cdx1*, and *Cdx2*, were not bivalent (Figure 7, D and L), suggesting their activation requires further selection in vivo, consistent with their lack of induction after EED inactivation in 1014 cells in vitro (Figure 3J). Together, these findings suggest that H3K27me3/H3K4me3 bivalency poises RAS/PI3K/MAPK pathway genes and *Neurod1* for robust activation after EED loss.

### EED inactivation at tumor initiation prevents emergence of SCLC histology in an EGFR-mutant GEMM with Rb1 and Trp53 inactivation after EGFR withdrawal.

Our data showed that EED supports ASCL1-positive SCLC histology in the RPR2 GEMM, with EED inactivation promoting LUAD histology (Figures 1–7). We therefore hypothesized that EED/PRC2 activity may also be important for acquisition of SCLC histology during LUAD-to-SCLC transformation, a resistance mechanism observed in *EGFR*-mutant LUAD tumors harboring *RB1* and *TP53* alterations (13). To test this, we developed an *EGFR*-mutant LUAD GEMM capable of histological transformation under selective pressure from EGFR suppression. We used a doxycycline-inducible (Dox-inducible) EGFR L858R transgenic model (49, 50) crossed with *Trp53*^fl/fl^, LSL rtTA, and LSL-Cas9-P2A-GFP mice (Figure 8A). We then generated adenoviruses encoding CMV-Cre to activate Cas9 and rtTA expression, sgRNAs targeting *Rb1*, and either an sgRNA targeting *Eed* or a nontargeting control sgRNA, generating *EGFR*-mutant LUAD GEMMs with concurrent *Rb1* and *Trp53* inactivation that were either EED-inactivated (*EGFR*-mutant ERP) or EED-WT (*EGFR*-mutant CRP). Immunoblotting validated Cre expression and efficient RB1 and EED inactivation (Supplemental Figure 12A).

Adenoviruses were intratracheally injected, and 1 week later mice were started on Dox chow to induce EGFR L858R expression. Pilot experiments showed that after 20 weeks on Dox, mice developed dyspnea with lung pathology consistent with *EGFR*-mutant LUAD, matching the expected latency of this model (Supplemental Figure 12, B and C) (49, 50). Therefore, after approximately 20 weeks, *EGFR*-mutant CRP and ERP mice were randomized to remain on Dox (Dox ON) or switch to regular chow (Dox OFF) to suppress EGFR L858R expression and were followed to endpoint (Figure 8B). Consistent with EGFR L858R acting as a potent oncogene, *EGFR*-mutant CRP and ERP Dox ON mice succumbed more rapidly, with median overall survival of 30.3 and 31.0 weeks, respectively. Survival was prolonged in Dox OFF mice, with median overall survival of 57.9 and 57.1 weeks for EGFR-mutant CRP and ERP cohorts, respectively (Figure 8C).

*EGFR*-mutant CRP Dox OFF and ERP Dox OFF mice developed distinct recurrent disease phenotypes. Tumors recurred in *EGFR*-mutant CRP Dox OFF mice, with 7 of 12 developing spontaneous metastases to liver, kidney, and brain, whereas no *EGFR*-mutant ERP Dox OFF mice developed distant metastases (Figure 8D, Supplemental Figure 12D, and Supplemental Table 7, tab 1). This was consistent with the absence of distant metastases when EED was inactivated at tumor initiation in the RPR2 GEMM (Figure 1F). Despite this difference, median overall survival was similar between *EGFR*-mutant CRP Dox OFF and ERP Dox OFF mice (Figure 8C), consistent with primary lung tumor burden and respiratory compromise driving endpoint in both groups (Supplemental Figure 12, E and F, and Supplemental Table 7, tab 1). Because analyses were performed at endpoint, we cannot distinguish whether EED loss suppresses early dissemination versus later metastatic outgrowth. However, recurrent tumors still developed in *EGFR*-mutant ERP Dox OFF mice, indicating that the reduction in metastasis does not simply reflect absence of recurrent disease.

IHC validated Dox-dependent EGFR L858R expression, GFP expression in tumor cells, and complete EED loss only in *EGFR*-mutant ERP tumors (Supplemental Figure 12G). DNA sequencing and immunoblotting confirmed expected genetic inactivation patterns in *EGFR*-mutant CRP and ERP tumors (Supplemental Figure 12, C, H, and I). Consistent with *RB1* and *TP53* alterations increasing SCLC transformation risk after EGFR inhibition in human *EGFR*-mutant LUAD (13), tumors in 3 of 12 *EGFR*-mutant CRP Dox OFF mice recurred as SCLC, whereas no *EGFR*-mutant ERP Dox OFF mice developed SCLC histology (0 of 12) (Figure 8E). IHC showed that transformed SCLC tumors in *EGFR*-mutant CRP Dox OFF mice either expressed ASCL1 alone or heterogeneously expressed ASCL1 and NEUROD1, whereas *EGFR*-mutant ERP counterparts were negative (Figure 8F and Supplemental Table 7, tab 2). This resembles human *EGFR*-mutant transformed SCLC, which shows heterogeneous transcription factor expression and enrichment for variant subtypes, including NEUROD1 (51), and supports transformation as a mechanism of escape from EGFR L858R suppression rather than de novo SCLC formation, as NEUROD1 expression is not observed in the RPR2 SCLC GEMM (19, 20, 31, 52). Consistent with the EED-inactivated RPR2 model, *EGFR*-mutant ERP Dox ON and Dox OFF tumors also showed LUAD with mucinous features (Figure 8F).

Bulk RNA-seq confirmed increased neuroendocrine markers, including *Syp*, *Insm1*, and *Ncam1*, as well as *Ascl1*, heterogeneous *Neurod1*, and enrichment for SCLC-related signatures, including MYC, G2M, and pancreas β cell programs, in *EGFR*-mutant CRP Dox OFF transformed SCLC tumors relative to *EGFR*-mutant CRP Dox ON, *EGFR*-mutant CRP Dox OFF LUAD, and *EGFR*-mutant ERP tumors (Figure 8G, Supplemental Figure 13, A–C, and Supplemental Table 7, tab 3). Transformed SCLC tumors also had lower MAPK target gene expression (Supplemental Figure 13D). *EGFR*-mutant CRP Dox OFF LUAD tumors were higher-grade tumors than *EGFR*-mutant CRP Dox ON LUAD tumors and were enriched for EMT signatures, consistent with poorly differentiated histology and increased metastatic propensity (Supplemental Figure 13E and Supplemental Table 7, tab 1). In line with the EED-inactivated RPR2 model, mucinous LUAD, tumor-associated macrophages, and neutrophil signatures were enriched in *EGFR*-mutant ERP Dox ON and Dox OFF tumors relative to *EGFR*-mutant CRP counterparts (Supplemental Figure 13, A, F, and G, and Supplemental Table 7, tab 4). Together, these data demonstrate that *EGFR*-mutant LUAD tumors with *Rb1* and *Trp53* loss can recur after EGFR oncogene withdrawal through distinct trajectories, including SCLC transformation or aggressive metastatic LUAD recurrence. EED inactivation prevents emergence of SCLC histology and reduces spontaneous metastasis, but does not prevent recurrence, instead redirecting tumor evolution toward LUAD with mucinous differentiation (Supplemental Figure 13H).

Although these studies demonstrate that EED inactivation at tumor initiation prevents SCLC histology after EGFR oncogene withdrawal, they do not determine whether pharmacologic EED inhibition suppresses SCLC transformation during tumor maintenance. Because therapeutic studies in the EGFR ON/OFF GEMM would require prolonged treatment in a long-latency autochthonous model, we tested whether pharmacologic EED inhibition attenuates early neuroendocrine changes after EGFR withdrawal in vitro. We generated 3 independent cell lines — 1525, 1533, 1544 — from *EGFR*-mutant CRP tumors maintained on Dox, each with EGFR L858R expression and *Rb1* and *Trp53* inactivation (Figure 8H). EGFR withdrawal reduced proliferation in 2 of 3 lines (Supplemental Figure 13I). In all 3 lines, EGFR withdrawal induced the neuroendocrine marker INSM1 (53), which was attenuated by the clinical-grade EED inhibitor MAK683 (54), while MAK683 completely suppressed H3K27me3, confirming on-target PRC2 inhibition (Figure 8I). RNA-seq in 1525 cells showed that EGFR withdrawal enriched gene signatures associated with SCLC, including neuroendocrine, E2F target, G2M checkpoint, and MYC-associated signatures (15), which were attenuated by MAK683 (Figure 8J). Thus, pharmacologic EED inhibition attenuates INSM1 induction and selected SCLC- and proliferation-associated programs following EGFR withdrawal in vitro. However, these experiments do not establish that pharmacologic EED inhibition suppresses SCLC transformation in established tumors in vivo, which requires future study.

### H3K27me3 represses LUAD oncogenic programs in human SCLC through bivalent silencing of RAS, PI3K, and MAPK pathway genes.

Having identified that PRC2 represses LUAD oncogenic programs in murine SCLC models, we next asked whether similar bivalent repression patterns occur in human lung cancer. We analyzed H3K27me3 and H3K4me3 ChIP-seq data from 5 de novo neuroendocrine SCLC patient-derived xenografts (PDXs) and 4 de novo *EGFR*-mutant LUAD PDXs (Figure 9, A, B, and E) (55). ChEA and ENCODE analyses of H3K27me3 peaks identified SUZ12 and EZH2 as dominant regulators in both histologies, validating the dataset (Supplemental Figure 14, A and B). Consistent with our murine data and prior studies (47, 48), Gene Ontology analysis showed that MHC class I antigen presentation genes were enriched for H3K27me3 in SCLC, whereas axon guidance pathways were enriched for H3K27me3 in LUAD (Supplemental Figure 14, C and D), indicating that H3K27me3 repression patterns distinguish key features of SCLC and LUAD. De novo neuroendocrine SCLC had more numerous and stronger H3K27me3 peaks than LUAD, with top SCLC-gained H3K27me3 peaks including PI3K and MAPK pathway genes (Figure 9, C and D, and Supplemental Table 6, tab 3), mirroring our GEMM findings. Consistently, bulk H3K27me3 was elevated in *EGFR*-mutant CRP Dox OFF tumors that transformed to SCLC relative to *EGFR*-mutant CRP Dox ON LUAD tumors (Supplemental Figure 14, E and F).

We next identified bivalent promoters comarked by H3K27me3 and H3K4me3 in these human PDXs. SCLC PDXs contained 548 bivalent genes compared with 202 in LUAD (Figure 9F, Supplemental Figure 14G, and Supplemental Table 6, tab 4). Bivalent genes in SCLC were enriched for RAS, PI3K/AKT, and MAPK signaling, including *RASGRP1* (Figure 9, G and H), and were largely distinct from those in LUAD (Supplemental Figure 14, H and I). Notably, approximately 90% of RAS/PI3K/AKT/MAPK pathway genes that gained H3K27me3 in SCLC were bivalent (Figure 9I), and the genome-wide H3K27me3 signal was higher across these pathways in SCLC than in LUAD (Figure 9J). Finally, because *RB1* and *TP53* loss are nearly universal in SCLC (10), common in LUAD tumors that transform to SCLC (13), and *RB1* loss promotes E2F activity that drives PRC2 component expression (56), we asked whether *RB1/TP53* inactivation was associated with elevated PRC2 expression in human LUAD. Indeed, *RB1/TP53*-mutant LUAD tumors expressed higher levels of *EZH2*, *EED*, and *SUZ12* than *RB1/TP53*-WT LUAD tumors (Figure 9, K–M, and Supplemental Figure 14, J–L). Together, these data provide evidence in human tumors that H3K27me3 represses LUAD-associated oncogenic signaling programs through bivalent chromatin in SCLC, supporting a mechanism through which the PRC2 complex promotes the neuroendocrine phenotype.

## Discussion

Here, we demonstrate that EED, a core PRC2 component, supports the ASCL1-positive neuroendocrine identity and histological state of SCLC during tumorigenesis. In the RPR2 genomic context, which normally drives SCLC formation, tumors largely escaped EED inactivation by adopting LUAD histology. Conversely, in an *EGFR*-mutant LUAD GEMM with *Rb1* and *Trp53* inactivation, EED inactivation at tumor initiation prevented emergence of SCLC histology following EGFR oncogene withdrawal. Together, these findings identify EED as an important regulator of SCLC neuroendocrine identity and lung cancer histological state selection. During revision, Kopparam et al. similarly reported that PRC2 loss alters SCLC tumorigenesis and creates vulnerabilities to combined EZH2 and G9a/GLP inhibition in established SCLC models (57). Our findings extend these observations by showing that PRC2/EED regulates lung cancer histological identity in vivo, including the ASCL1-positive neuroendocrine phenotype and *EGFR*-mutant LUAD-to-SCLC transformation.

Mechanistically, EED directly binds and promotes H3K27me3-mediated repression of RAS, PI3K, and MAPK signaling genes, as well as NEUROD1, providing a mechanism to suppress LUAD oncogenic signaling and support ASCL1-positive SCLC identity. These loci are bivalent, marked by H3K27me3 and H3K4me3, rendering them poised for activation upon PRC2 loss. Thus, EED inactivation promotes LUAD histology by derepressing bivalent targets, likely through a NEUROD1-positive intermediate state requiring tumor microenvironmental cues (Supplemental Figure 15). ChIP-seq analysis of human neuroendocrine SCLC PDXs revealed bivalent chromatin also at RAS, PI3K, and MAPK pathway genes, supporting conservation of this PRC2-dependent mechanism in human SCLC.

Human lung cancers can exist as combined LUAD/SCLC tumors and can transform from LUAD to SCLC as a resistance mechanism to targeted therapy (2, 3). Our data suggest that mechanisms promoting PRC2 activity may favor SCLC histology in these settings. Although SCLC-to-LUAD transformation is rarely observed clinically, this may reflect limited efficacy of current SCLC therapies and insufficient selective pressure to escape the neuroendocrine state. With approval of the DLL3 bispecific T cell engager tarlatamab (58, 59), it is important to determine whether resistance to effective neuroendocrine-targeted therapies can involve histological transformation to NSCLC. Consistent with this possibility, a recent case report described acquired tarlatamab resistance associated with low DLL3 expression and transformation to NSCLC (60).

EED inactivation in both SCLC RPR2 GEMMs and *EGFR*-mutant RP LUAD GEMMs produced LUAD tumors with aberrant gastrointestinal differentiation programs. Prior work similarly showed that EED loss promotes mucinous LUAD histology in *KRAS*-mutant LUAD GEMMs (25, 26), suggesting that PRC2 normally represses gastrointestinal differentiation in lung tumors. Our findings extend this work by demonstrating that EED also supports the ASCL1-positive SCLC state and constrains LUAD oncogenic signaling in an *Rb1/Trp53*-inactivated SCLC context. This function may mirror foregut endoderm patterning, where PRC2 likely represses gastrointestinal differentiation programs such as HNF4A, WNT, and FGF signaling to promote respiratory fate (61). EED inactivation also derepressed NEUROD1 in vitro and promoted ASCL1-to-NEUROD1 subtype switching in vivo. We found that NEUROD1 is a direct bivalent EED target, enabling rapid activation upon PRC2 loss. Thus, whereas our prior work showed that KDM6A maintains an epigenetic state permissive for the ASCL1 subtype by activating ASCL1-associated genes (20), this study suggests that PRC2 supports the ASCL1-positive subtype by repressing NEUROD1-associated programs.

Following EED inactivation, NEUROD1-positive SCLC tumors were rarely sustained, and nearly all mice ultimately developed LUAD tumors, suggesting that NEUROD1-positive SCLC tumors are transient, selected against, or give rise to LUAD. We observed NEUROD1-positive cells within LUAD tumors that emerged after EED loss, and snRNA-seq identified a NEUROD1-expressing, EMT-like subpopulation, supporting a model in which LUAD arises from SCLC through a NEUROD1-positive intermediate. EED loss alone was insufficient to induce LUAD in vitro, implying that the tumor immune microenvironment helps promote LUAD histology in vivo, potentially by enhancing LUAD oncogenic signaling. Alternatively, PRC2 loss increases MHC class I expression in SCLC (47, 48), which may enhance immune visibility and clearance of neuroendocrine SCLC cells and favor LUAD outgrowth as a potential immune evasion state. Future studies will determine whether NEUROD1 functionally promotes LUAD emergence from SCLC, how the tumor immune microenvironment contributes to state selection, and whether histological transformation reflects tumor-intrinsic reprogramming, immune selection, or both.

LUAD-to-SCLC transformation is a well-established resistance mechanism following targeted therapy, most often in EGFR-mutant LUAD tumors with co-occurring RB1 and TP53 alterations (2, 3, 5). Although these alterations increase transformation risk by 43-fold (13), only a subset of patients undergo SCLC transformation clinically (8), and similarly only a subset of tumors in our EGFR-mutant model developed SCLC histology after EGFR oncogene withdrawal. This incomplete penetrance likely reflects the absence of additional co-occurring events, such as PI3K pathway activation or MYC overexpression, that are frequently associated with transformation (5, 14, 15). Future studies will test the requirement for EED in additional EGFR-mutant transformation models incorporating these cooperating alterations.

Importantly, although EED inactivation at tumor initiation prevented SCLC histology and reduced spontaneous metastasis after EGFR oncogene withdrawal in *EGFR*-mutant LUAD tumors with *Rb1* and *Trp53* loss, it did not eliminate tumor recurrence. Instead, EED loss redirected tumor evolution toward mucinous LUAD states. Thus, EED inactivation reshapes resistance trajectories and tumor state selection rather than fully suppressing resistance, highlighting potential trade-offs associated with EED inhibition.

There are limitations to this study. Because tumors were initiated with CMV-Cre and analyzed at endpoint, we cannot exclude the possibility that LUAD tumors arising after EED inactivation originated from a distinct cell of origin rather than from histological transformation of neuroendocrine SCLC. However, EED inactivation in ASCL1-positive SCLC derepressed NEUROD1, and clonally related NEUROD1-positive cells were interspersed within EED-inactivated LUAD tumors, an observation not seen in other LUAD GEMMs, supporting a model in which LUAD arises from neuroendocrine tumor cells. Definitive proof will require lineage tracing and EED inactivation in established SCLC tumors. Future studies will use lung cell type–specific promoters, lineage tracing, and genetic or pharmacologic EED inactivation in established tumors to define its role during tumor maintenance.

Our findings nominate PRC2/EED inhibition for future preclinical investigation in EGFR-mutant LUAD tumors with co-occurring RB1 and TP53 inactivation, a genotype associated with increased SCLC transformation risk (8, 13). However, EED was genetically inactivated at tumor initiation, not pharmacologically inhibited in established EGFR-driven tumors. In addition, short-term MAK683 treatment attenuated INSM1 induction and selected SCLC- and proliferation-associated programs (15) after EGFR oncogene withdrawal in vitro but did not model complete SCLC transformation. Future studies are required to determine whether clinical-grade EED inhibitors can alter LUAD-to-SCLC transformation in established tumors driven by EGFR or other LUAD oncogenic drivers, such as ALK rearrangements or KRAS mutations, where SCLC transformation has also been observed, albeit less commonly (62).

## Methods

Additional detailed experimental methods are provided in the Supplemental Methods.

### Sex as a Biological Variable.

For all studies with the RPR2 and *EGFR*-mutant GEMMs, both male and female mice were used at roughly equal numbers. For the SCLC xenograft model in BL6J and NCr nude mice in Figure 5, only female mice were employed for ease of use. Given these were transplantation xenograft experiments, we expect the findings to be relevant to more than one sex; however, sex-specific effects were not formally assessed in the transplantation experiments.

### Statistics.

For GSEA, RNA-seq, ATAC-seq, and ChIP-seq enrichment analyses, statistical significance was corrected for multiple hypothesis testing. Survival was analyzed by log-rank test (Figure 1B and Figure 8C), correlations by Pearson’s correlation (Supplemental Figure 2E), and GREAT analysis by binomial *P* value as described previously (63). For Figure 5D, Figure 9, K–M, and Supplemental Figure 14, J–L, 1-way ANOVA followed by multiple comparisons adjusted by Tukey’s test was used to calculate adjusted 2-sided *P* values. Unless otherwise indicated, all other comparisons used unpaired, 2-sided Student’s *t* test. *P* values less than 0.05 were considered statistically significant. Data are presented as the mean ± SEM unless otherwise indicated.

The number of independent biological experiments is indicated in each figure legend. Representative immunoblots are shown for Figures 3I, Figure 6H, Supplemental Figure 3J, Supplemental Figure 9, B and F, and Supplemental Figure 10A, each performed in at least 3 biologically independent experiments. Immunoblots in Figure 5C, Figure 6D, Supplemental Figure 12C, and Supplemental Figure 14E contain multiple independent tumors. Representative H&E and IHC images are from independent tumors from independent mice. For Figure 1, 48 mice were randomized to sg*Eed* RPR2 or sgControl RPR2 adenovirus; for Figure 8, 59 mice were randomized to sg*Eed* RP or sgControl RP adenovirus. Sequencing cores/vendors were blinded to sample identity. Cell culture experiments were repeated in at least 3 biologically independent experiments or as indicated. Sample sizes were not predetermined statistically but are similar to prior studies (28, 29). Data distribution was assumed normal but not formally tested. Cell culture experiments were not blinded. No data were excluded.

### Study approval.

All mouse experiments complied with NIH guidelines and were approved by Dana-Farber Cancer Institute (DFCI) Animal Care and Use Committee (DFCI protocol 19-009). All adenoviral and lentiviral transduction experiments complied with the Biohazard Control Committee (DFCI protocol 19-1133).

### Data availability.

Processed bulk RNA-seq differential expression and FPKM data are provided in Supplemental Tables 2–4 and 7. Processed H3K27ac ChIP-seq, EED/H3K27me3 ChIP-seq, H3K27me3/H3K4me3 ChIP-seq, and ATAC-seq peak data are provided in Supplemental Tables 2–4 and 6. Raw and processed sequencing data have been deposited in the Gene Expression Omnibus under accession numbers GSE293599 (bulk RNA-seq), GSE293598 (ChIP-seq), GSE293600 (ATAC-seq), GSE293597 (scRNA-seq), and GSE293596 (snRNA-seq). Data points are provided in the Supporting Data Values file. This study did not use custom code. Additional data and materials are available from the corresponding author upon reasonable request.

## Author contributions

YL and MGO conceptualized the study and methodology; performed investigation, validation, data curation, analysis, and visualization; and wrote the manuscript. MGO also provided resources, supervision, project administration, and funding. YNL, HC, GRDO, and TEZ performed investigation, validation, data curation, and analysis. MV, YTD, AD, XQ, SK, RL, RTB, SL, WL, MVO, VS, and MDS performed investigation. KP, JBE, MLF, HWL, SS, and DAB provided resources, supervision, and project administration. YC and HJ provided editorial feedback.

## Conflict of interest

MGO reports grants from Novartis, Circle Pharma, Amgen, Auron Therapeutics, Eli Lilly, Takeda, and BMS. KP reports grants from AstraZeneca, Roche/Genentech, Boehringer Ingelheim, and D2G Oncology; personal fees from AstraZeneca and Revelio Therapeutics; and royalties from Memorial Sloan Kettering Cancer Center/MolecularMD for EGFR T790M mutation testing. KP is cofounder of and consultant for Revelio Therapeutics. MLF is a cofounder and reports personal fees and other support from Precede Biosciences.

## Funding support

This work is the result of NIH funding, in whole or in part, and is subject to the NIH Public Access Policy. Through acceptance of this federal funding, the NIH has been given a right to make the work publicly available in PubMed Central.

- NIH grant P01CA295524 (to MGO and DAB).
- NIH grant K99CA304214 (to YL).
- NIH grant P50CA265826 (to MGO).
- NIH grant R01CA263715 (to KP).
- NIH grant R50CA251956 (to SL).
- American Cancer Society grant PF-24-1252572-01-CCB (to YL).
- The Neil S. Hirsch Foundation (to MGO and YL).
- The Kaplan Family Fund (to MGO).
- NIH shared instrumentation grant 1S10OD036228-01 for the DFCI Molecular Biology Core Facility’s Illumina NovaSeq X Plus.
- National Cancer Institute Cancer Center support grant NIH 5 P30 CA06516 to the Dana-Farber/Harvard Cancer Center Specialized Histopathology Core.

## Supplementary Material

Supplemental data

Unedited blot and gel images

Supplemental table 1

Supplemental table 2

Supplemental table 3

Supplemental table 4

Supplemental table 5

Supplemental table 6

Supplemental table 7

Supporting data values

## Figures and Tables

**Figure 1:**
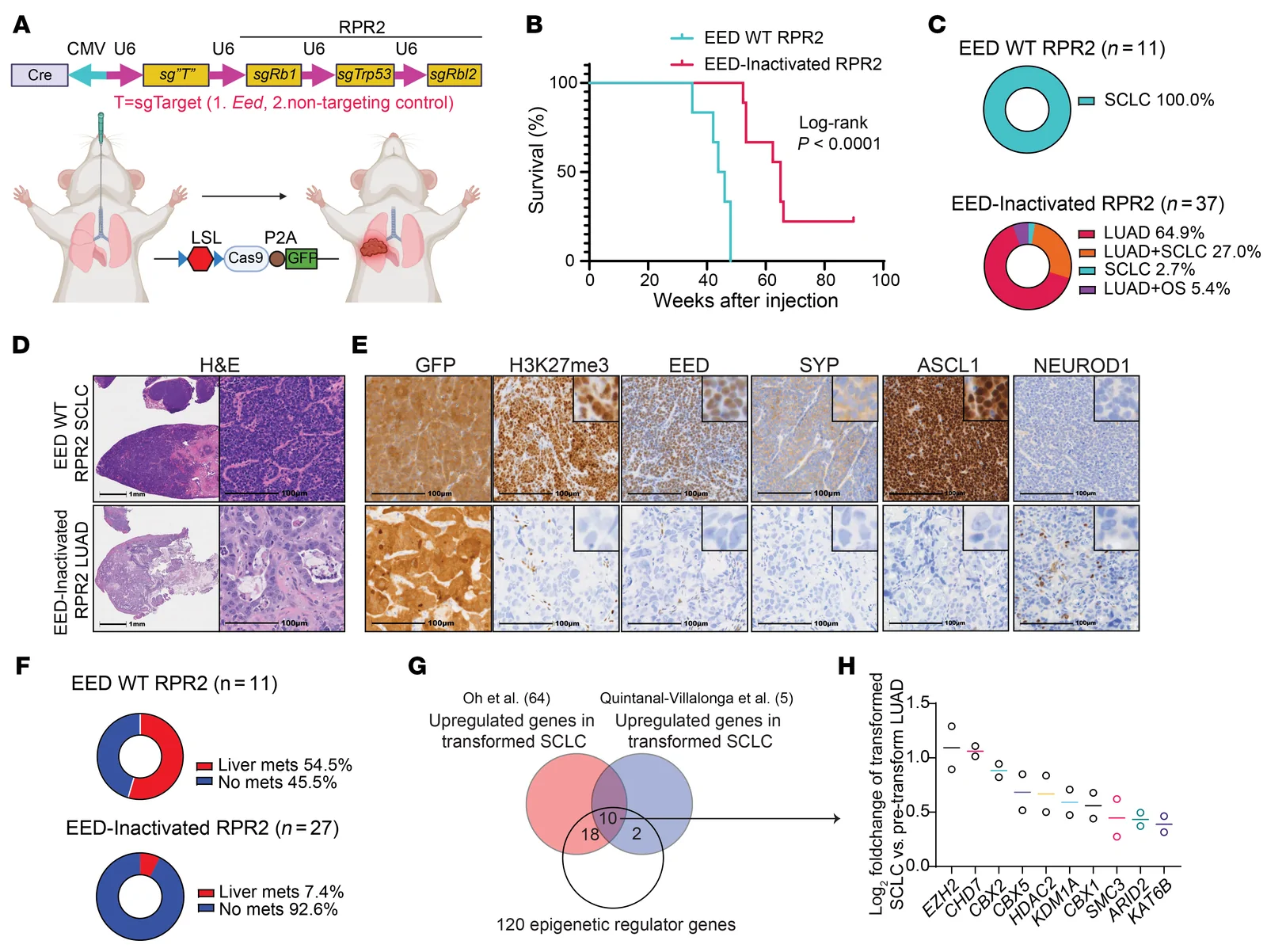
EED inactivation promotes a shift from SCLC toward LUAD histology in an RPR2 GEMM in vivo. (**A**) Schematic of the adenovirus used for intratracheal injection into the lungs of LSL-Cas9 mice to generate EED-inactivated or *EED*-WT autochthonous SCLC tumors. RPR2: sg*Rb1*, sg*Trp53*, sg*Rbl2*; sg“T”: sg*Eed* or sgControl (nontargeting sgRNA). (**B**) Kaplan-Meier survival estimate of LSL-Cas9 mice intratracheally injected with adenoviruses as indicated. Median overall survival: EED-WT RPR2, 44.93 weeks; EED-inactivated RPR2, 65.00 weeks. *n* = 6 EED-WT RPR2 mice, *n* = 9 EED-inactivated RPR2 mice. Log-rank (Mantel-Cox) test was used to calculate *P* values. (**C**) Pie chart of lung cancer histology in EED-inactivated RPR2 tumors (*n* = 37 tumors from 33 independent mice) and EED-WT RPR2 tumors (*n* = 11 tumors from 8 independent mice). OS: osteosarcoma. (**D** and **E**) Representative H&E (**D**) and IHC (**E**) staining for GFP, H3K27me3, EED, synaptophysin (SYP), ASCL1, and NEUROD1 in EED-WT RPR2 SCLC tumors (*n* = 11, 2, 2, 2, 2, 2, and 10, respectively) and EED-inactivated RPR2 LUAD tumors (*n* = 36, 6, 4, 5, 3, 3, and 10, respectively). Scale bars: 1 mm (**D**), 100 μm (**E**). Insets are ×9 magnification of the corresponding main image. (**F**) Pie chart of liver metastases from EED-inactivated RPR2 (*n* = 27 independent mice) and EED-WT RPR2 (*n* = 11 independent mice) mice. (**G**) Venn diagram of differentially upregulated genes in transformed SCLC versus pretransformed LUAD from Oh et al. (64) and Quintanal-Villalonga et al. (5) overlapped with 120 epigenetic regulator genes (see Supplemental Table 1, tab 3). (**H**) Dot plot of log_2_ fold change of RNA-seq expression of transformed SCLC versus pretransformed LUAD from **G**. See also Supplemental Figure 1.

**Figure 2:**
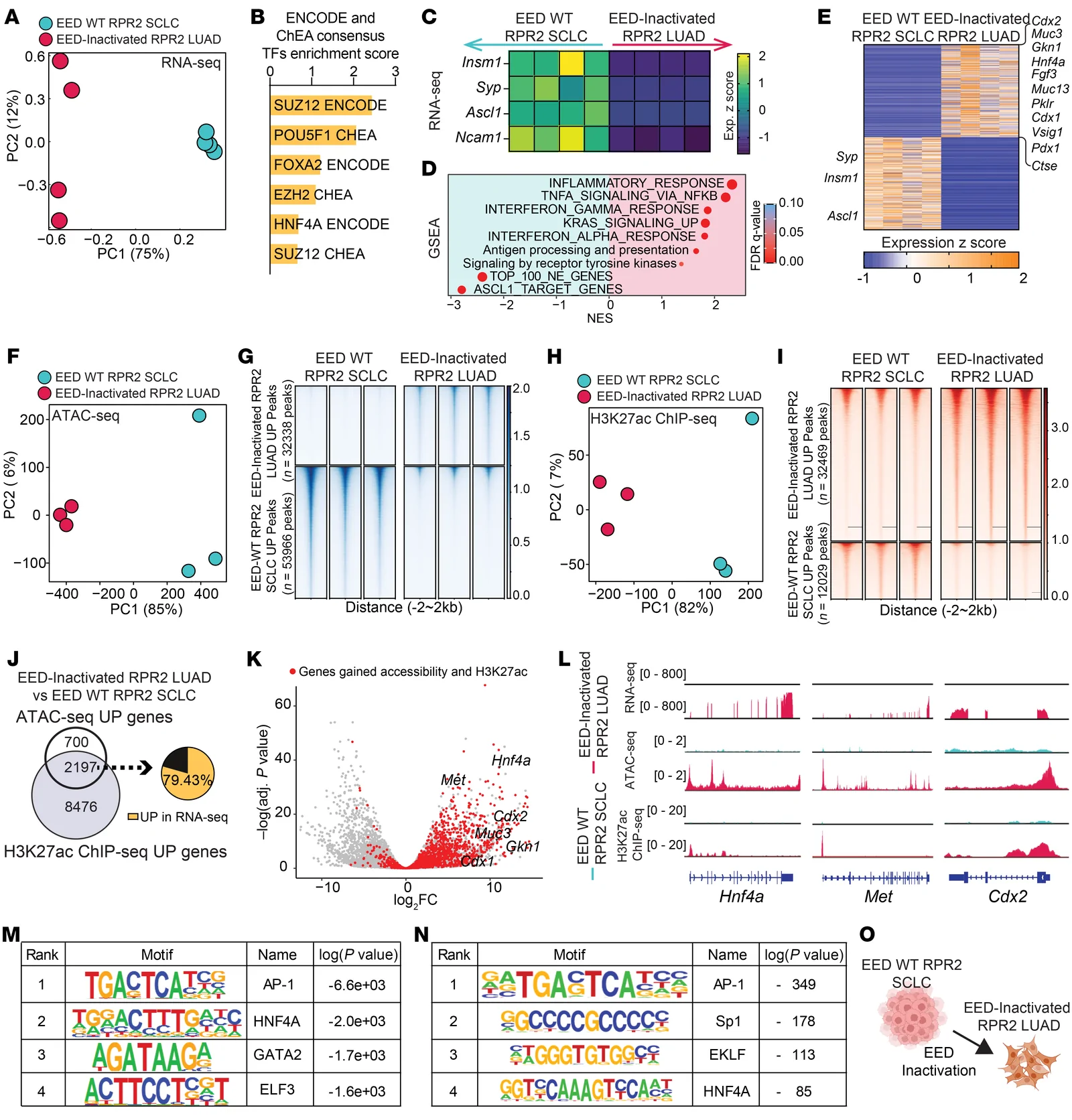
EED inactivation epigenetically restores LUAD oncogenic signatures in vivo. (**A**) Principal component analysis (PCA) of RNA-seq from 4 independent EED-WT RPR2 SCLC tumors (blue) and 4 independent EED-inactivated RPR2 LUAD tumors (red). (**B**) ENCODE/ChEA consensus transcription factor (TF) enrichment from RNA-seq in **A** showing top TF targets enriched in EED-inactivated RPR2 LUAD versus EED-WT RPR2 SCLC tumors. TFs with adjusted *P* value < 0.05 are shown. (**C**) Heatmap of neuroendocrine marker expression *z* scores from RNA-seq in **A**. Dark blue, low; yellow, high. (**D**) GSEA dot plot from RNA-seq in **A**. Dot color indicates FDR *q* value. Red, low; blue, high. Dot size indicates percentage of leading-edge genes. NES: normalized enrichment score. (**E**) Heatmap of top 500 differentially upregulated and downregulated genes ranked by *z* score. Red, high; blue, low. (**F** and **H**) PCA of ATAC-seq (**F**) and H3K27ac ChIP-seq (**H**) from 3 independent EED-WT RPR2 SCLC tumors (blue) and 3 independent EED-inactivated RPR2 LUAD tumors (red). (**G** and **I**) Heatmaps of differential ATAC-seq peaks (**G**) and H3K27ac ChIP-seq peaks (**I**). (**J** and **K**) Schematic (**J**) and volcano plot (**K**) showing differentially upregulated genes that gained both accessibility and H3K27ac in EED-inactivated RPR2 LUAD versus EED-WT RPR2 SCLC. log_2_FC: log_2_ fold change. (**L**) Averaged RNA-seq, ATAC-seq, and H3K27ac ChIP-seq tracks at *Hnf4a*, *Met*, and *Cdx2*. (**M** and **N**) HOMER motif analysis from ATAC-seq (**M**) and H3K27ac ChIP-seq (**N**). (**O**) Schematic showing escape from EED inactivation through SCLC-to-LUAD histological switching. Figure created with BioRender. See also Supplemental Figure 2.

**Figure 3:**
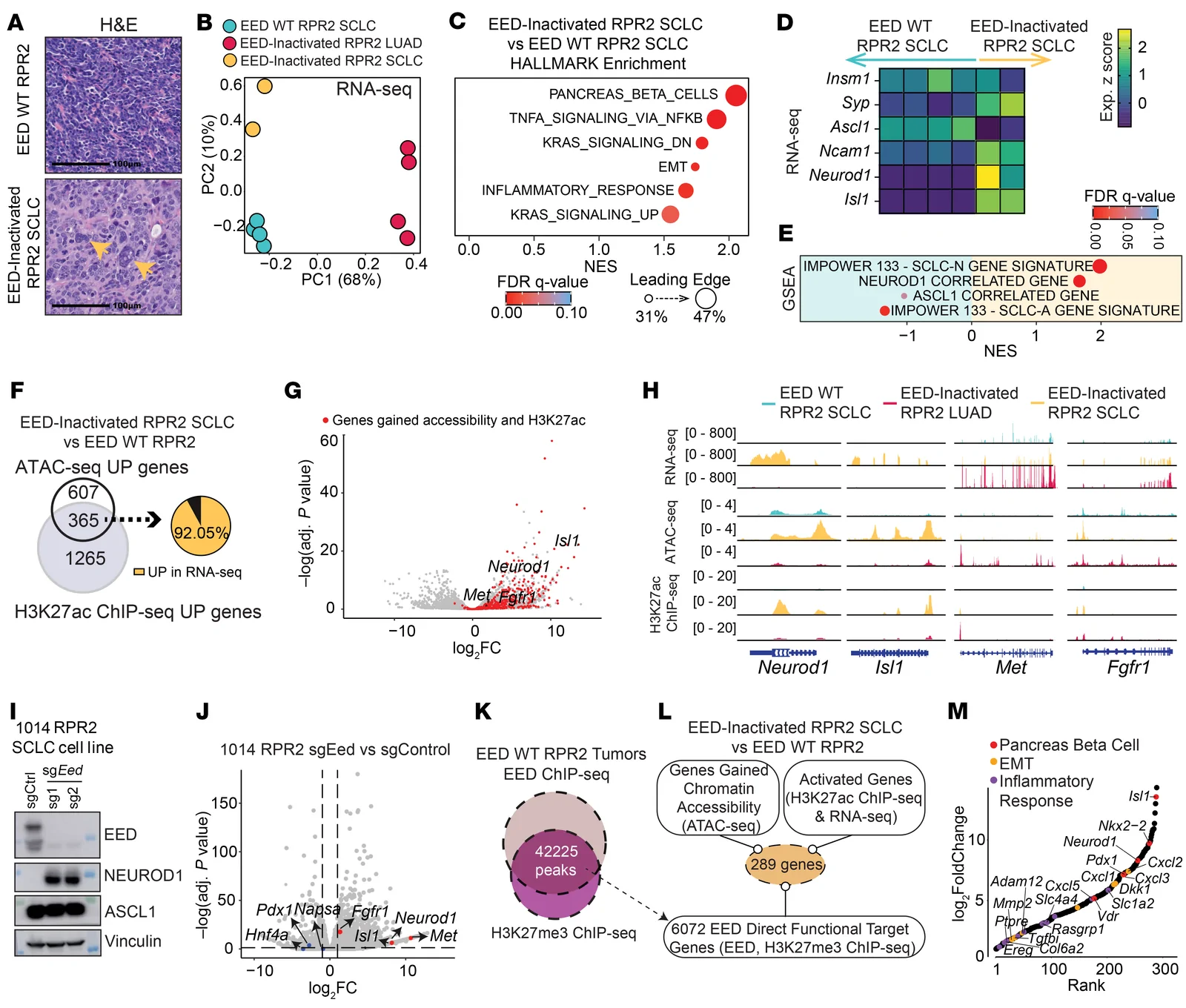
EED inactivation promotes rare NEUROD1-positive SCLC with early features of LUAD oncogenic signaling. (**A**) Representative H&Es of EED-WT (*n* = 11) and EED-inactivated (*n* = 2) RPR2 SCLC tumors. Yellow arrows indicate giant-cell features. Scale bars: 100 μm. (**B**) PCA of RNA-seq from EED-WT and EED-inactivated RPR2 SCLC and LUAD tumors. (**C**) Hallmark GSEA dot plot comparing EED-inactivated versus EED-WT RPR2 SCLC tumors. (**D**) Heatmap of neuroendocrine marker expression from *z* scored RNA-seq in **B**. Dark blue, low; yellow, high. (**E**) GSEA dot plot of indicated gene sets from RNA-seq in **B**. For **C** and **E**, dot color indicates FDR *q* value. Red, low; blue, high. Dot size indicates percentage of leading-edge genes. NES: normalized enrichment score. (**F** and **G**) Schematic (**F**) and volcano plot (**G**) showing upregulated genes that gained both accessibility and H3K27ac in EED-inactivated versus EED-WT RPR2 SCLC. log_2_FC, log_2_ fold change. (**H**) Averaged RNA-seq, ATAC-seq, and H3K27ac ChIP-seq tracks at *Neurod1*, *Isl1*, *Met*, and *Fgfr1*. (**I** and **J**) Representative immunoblot from 3 experiments (**I**) and RNA-seq volcano plot (**J**) of 1014 RPR2 EED-WT SCLC cells transduced with sgRNAs targeting *Eed* or sgControl. In **J**, vertical lines indicate absolute fold change of 2; horizontal line indicates adjusted *P* value = 0.05. (**K**) Schematic identifying direct EED targets by overlap of EED and H3K27me3 ChIP-seq in EED-WT RPR2 SCLC tumors. (**L**) Schematic integrating RNA-seq, ATAC-seq, and H3K27ac ChIP-seq to identify direct functional EED targets in EED-inactivated SCLC. (**M**) Rank plot of RNA-seq log_2_ fold change of functional EED targets in EED-inactivated SCLC. EMT: epithelial-to-mesenchymal transition. See also Supplemental Figure 3.

**Figure 4:**
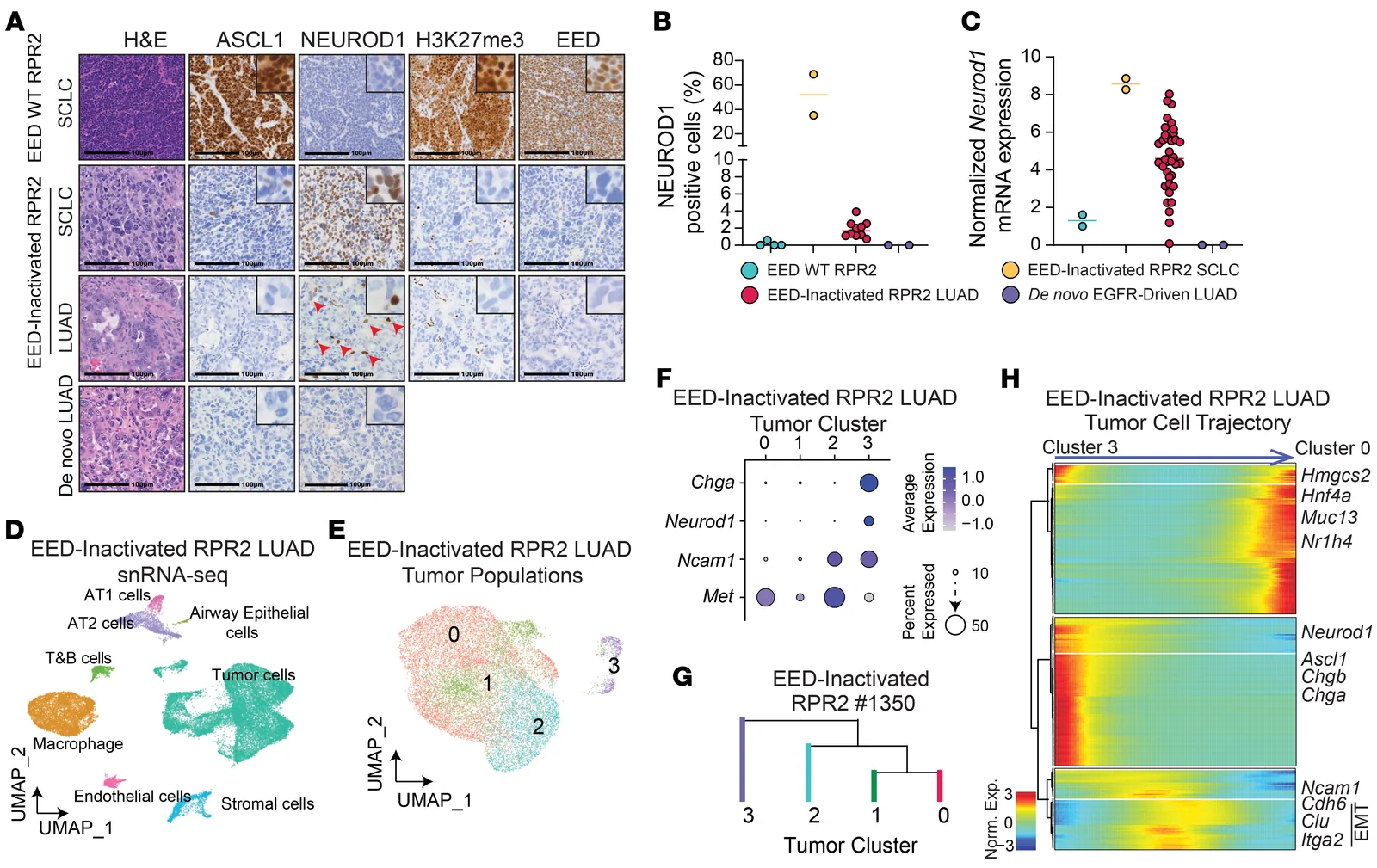
EED inactivation promotes LUAD histology likely through a NEUROD1-positive intermediate state. (**A**) Representative H&E and IHC for ASCL1, NEUROD1, H3K27me3, and EED in EED-WT RPR2 SCLC (*n* = 11, 2, 4, 2, and 2, respectively), EED-inactivated RPR2 SCLC (*n* = 2, 2, 2, 2, and 2, respectively), EED-inactivated RPR2 LUAD (*n* = 24, 3, 10, 4, and 5, respectively), and de novo LUAD tumors (*n* = 2, 2, and 2, respectively). Arrows indicate rare scattered NEUROD1-positive cells. Scale bars: 100 μm. Insets, ×9 magnification. (**B**) Quantitation of NEUROD1-positive cells from **A**. *n* = 4 EED-WT RPR2 SCLC, *n* = 2 EED-inactivated RPR2 SCLC, *n* = 10 EED-inactivated RPR2 LUAD, and *n* = 2 de novo LUAD tumors. (**C**) Normalized *Neurod1* mRNA expression by RT-qPCR in indicated GEMM tumors. *n* = 2 EED-WT RPR2 SCLC, *n* = 2 EED-inactivated RPR2 SCLC, *n* = 39 EED-inactivated RPR2 LUAD, and *n* = 2 de novo LUAD tumors. (**D** and **E**) UMAP of all cells (**D**) and tumor cells (**E**) from snRNA-seq of 3 EED-inactivated RPR2 LUAD tumors from independent mice. *n* = 27,117 total cells; *n* = 16,590 tumor cells. (**F**) Dot plot showing average expression of indicated genes in each tumor cell cluster. Dot size indicates percentage of expressing cells; color indicates expression level. Blue, high; white, low. (**G**) Phylogenetic tree of tumor cell clusters from EED-inactivated RPR2 LUAD tumor 1350 from **E** using maximum parsimony method. (**H**) Heatmap of normalized expression of top 300 trajectory-defining genes along the EED-inactivated RPR2 LUAD tumor cell trajectory. Blue, low; red, high. See also Supplemental Figures 4 and 5.

**Figure 5:**
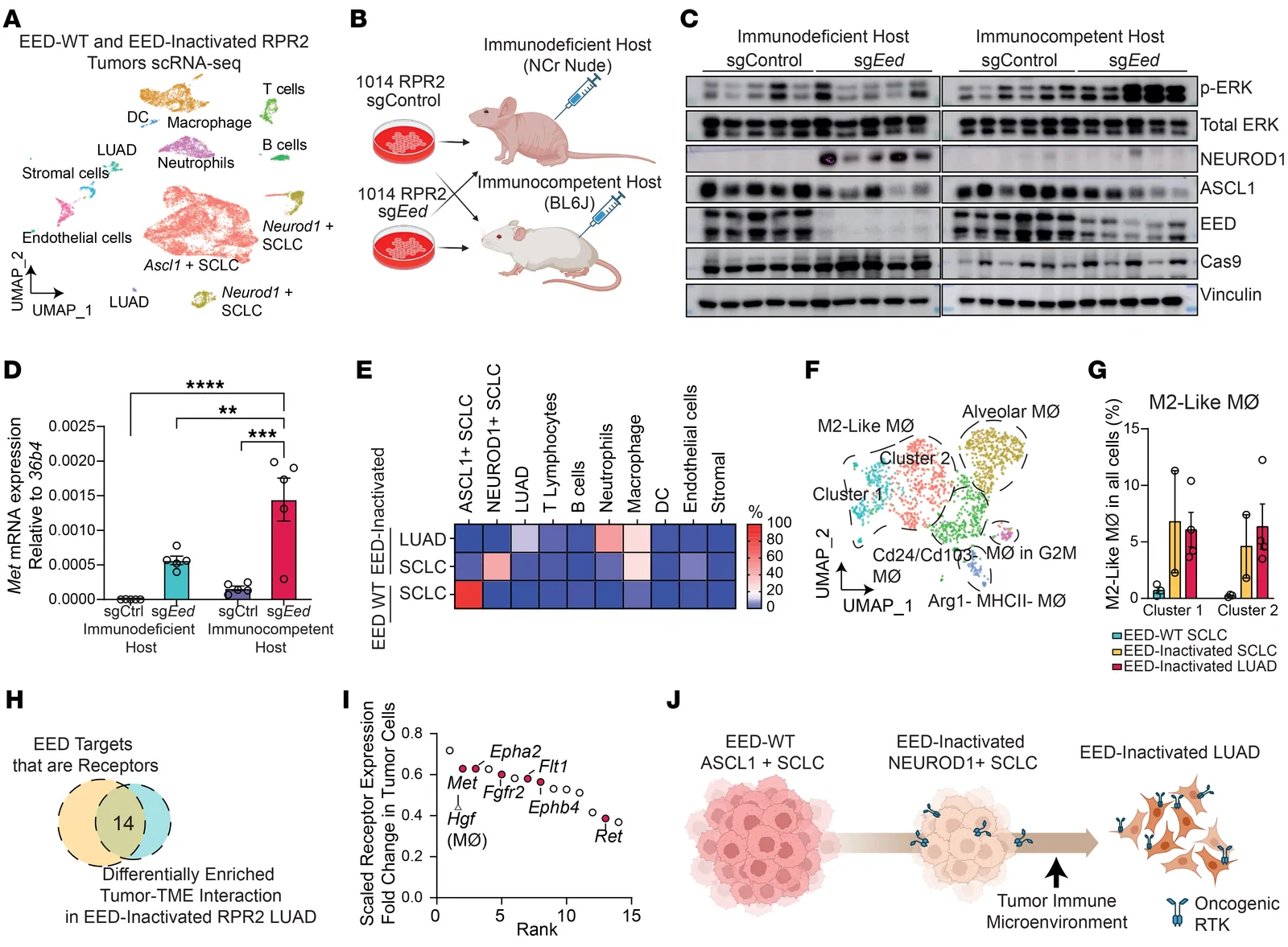
LUAD histology after EED inactivation requires cues from the tumor immune microenvironment. (**A**) UMAP of scRNA-seq from 3 independent EED-WT SCLC (*n* = 7,889 cells), 2 EED-inactivated SCLC (*n* = 2,064 cells), and 4 EED-inactivated LUAD (*n* = 2,013 cells) tumors; each tumor was from an independent mouse. Mϕ, macrophage. (**B**–**D**) Schematic (**B**), immunoblot (**C**), and RT-qPCR (**D**) of 1014 RPR2 EED-WT SCLC cells transduced with sg*Eed* or sgControl and implanted subcutaneously in immunodeficient NCr nude or immunocompetent BL6J mice. For **D**, 1-way ANOVA with Tukey’s test was used to calculate 2-sided *P* values adjusted for multiple comparisons. **P < 0.01, ***P <0.001, ****P < 0.0001. (**E**) Heatmap of indicated tumor and immune cell types from scRNA-seq in **A** comparing EED-inactivated LUAD, EED-inactivated SCLC, and EED-WT SCLC tumors. (**F** and **G**) UMAP (**F**) and quantification (**G**) of 2 M2-like macrophage clusters as percentages of total cells in each sample. (**H** and **I**) Schematic (**H**) and rank plot (**I**) of scaled receptor expression fold changes in EED-inactivated LUAD tumor cells for receptor-ligand interactions enriched in EED-inactivated LUAD versus EED-WT SCLC tumors and receptors upregulated after CRISPR-mediated EED inactivation in 1014 cells in vitro. For *Met*, the corresponding ligand gene, *Hgf*, and tumor microenvironment component with higher expression, macrophages, are indicated. (**J**) Schematic showing LUAD histology arising through a NEUROD1-positive transient intermediate state requiring cues from the tumor immune microenvironment. Figure created with BioRender. See also Supplemental Figures 6 and 7.

**Figure 6:**
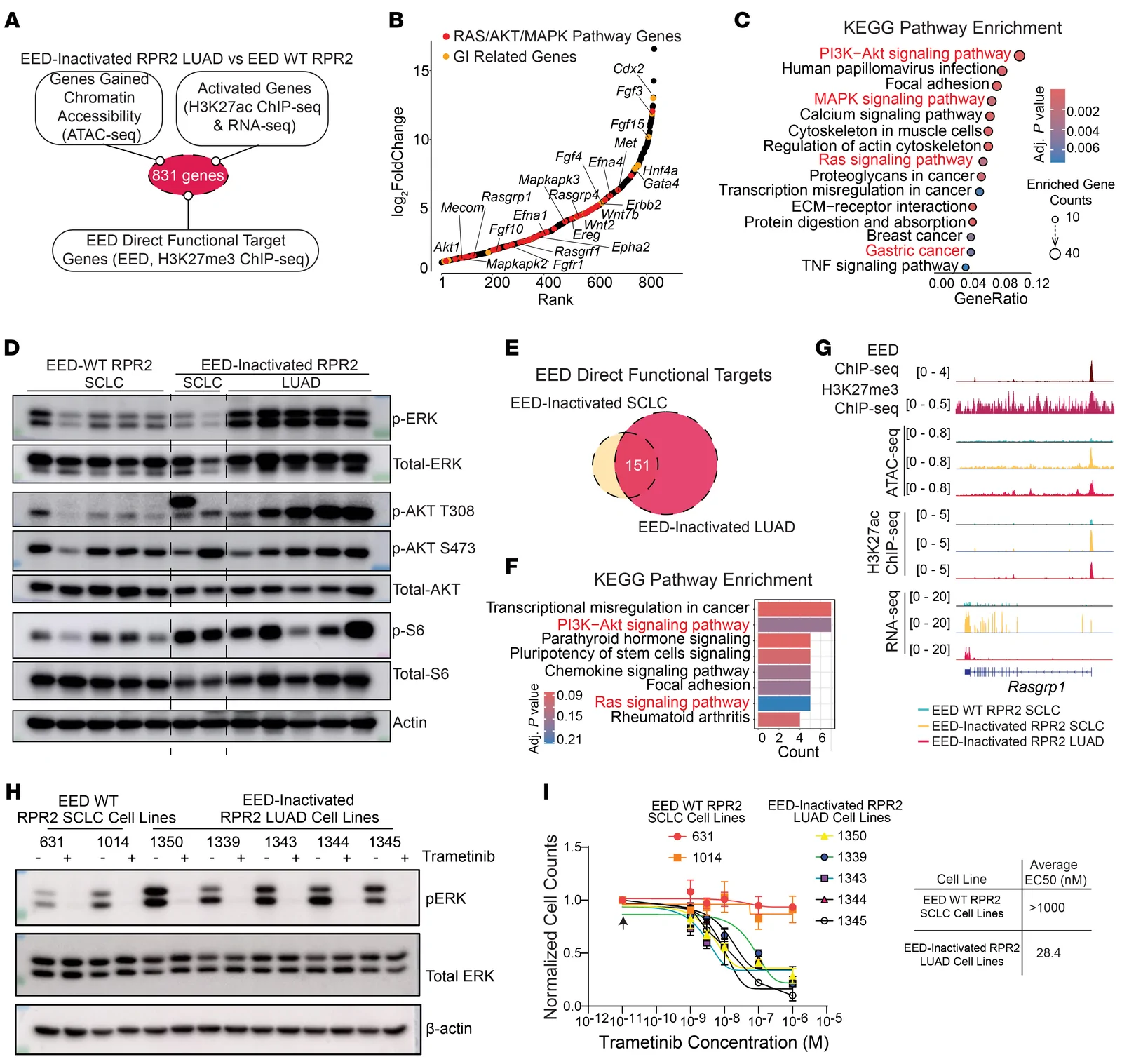
EED directly binds and represses RAS, PI3K, and MAPK pathway LUAD oncogenic signaling genes to support the SCLC neuroendocrine identity. (**A**) Schematic integrating direct EED targets with genes reactivated upon EED loss using RNA-seq, ATAC-seq, and H3K27ac ChIP-seq to identify direct functional EED targets in EED-inactivated LUAD tumors. (**B**) Rank plots of RNA-seq log_2_ fold change of functional EED targets in EED-inactivated LUAD tumors. (**C**) KEGG pathway enrichment of direct functional EED targets in EED-inactivated LUAD tumors. Dot color indicates Benjamini-Hochberg–adjusted (BH-adjusted) *P* value. Red, low; blue, high. Dot size indicates enriched gene counts. (**D**) Immunoblot of EED-WT SCLC, EED-inactivated SCLC, and EED-inactivated LUAD RPR2 GEMM tumors. (**E** and **F**) Schematic (**E**) and KEGG pathway enrichment analysis (**F**) of overlapping direct functional EED targets in EED-inactivated SCLC and LUAD. Color indicates BH-adjusted *P* value. (**G**) Averaged RNA-seq, ATAC-seq, and H3K27ac ChIP-seq tracks from EED-WT SCLC, EED-inactivated SCLC, and EED-inactivated LUAD tumors, and EED and H3K27me3 ChIP-seq tracks from EED-WT SCLC at *Rasgrp1*. (**H**) Immunoblot of cell lines from Supplemental Figure 9E treated overnight with 1 μM trametinib. (**I**) Normalized dose–response curves of cell lines from **H** treated with trametinib for 6 days, with EC_50_ values shown at right. If 50% killing was not reached at 1,000 nM, 1,000 nM was used for average EC_50_ calculation. Arrow indicates DMSO-treated sample used for normalization. *n* = 3 independent experiments. See also Supplemental Figures 8–10.

**Figure 7:**
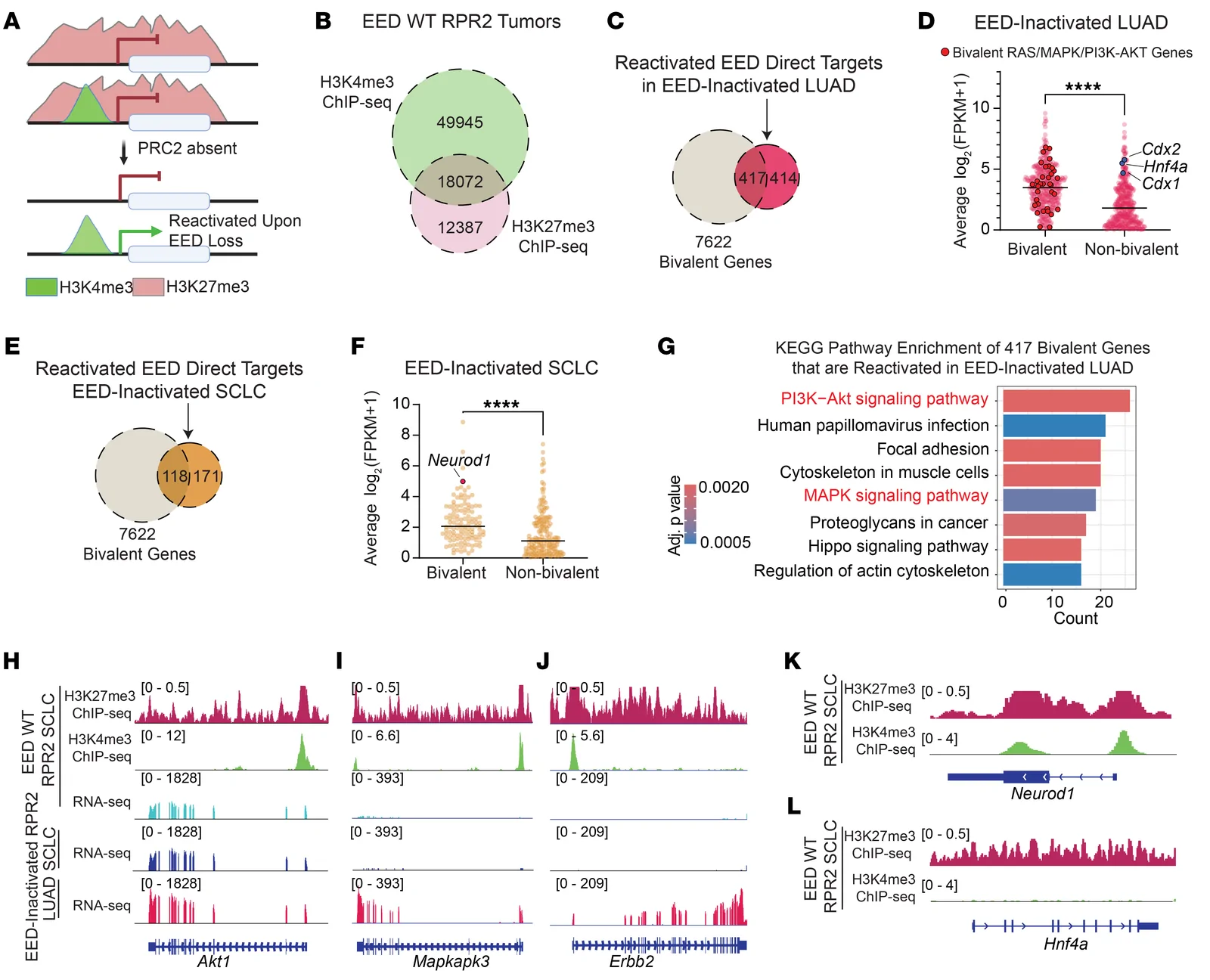
LUAD histology after PRC2 loss is associated with reactivation of bivalently marked RAS/PI3K/MAPK pathway genes and NEUROD1. (**A**) Schematic of genes with bivalent H3K4me3 and H3K27me3 marks poised for reactivation upon PRC2 loss. Figure created with BioRender. (**B** and **C**) Strategy to identify bivalent genes reactivated upon EED loss by overlapping H3K4me3 and H3K27me3 ChIP-seq peaks from EED-WT RPR2 SCLC tumors (**B**) with reactivated EED direct target genes in EED-inactivated RPR2 LUAD tumors (**C**). (**D**) Expression of reactivated EED direct targets that are bivalent or nonbivalent in EED-inactivated LUAD. (**E**) Identification of bivalent genes reactivated upon EED loss in EED-inactivated RPR2 SCLC using strategy from **B**. (**F**) Expression of reactivated EED direct targets that are bivalent or nonbivalent in EED-inactivated SCLC. (**G**) KEGG pathway enrichment of reactivated bivalent EED direct targets in EED-inactivated LUAD. Bar color indicates BH-adjusted *P* value. Red, low; blue, high. Count: enriched gene count. (**H**–**J**) Averaged H3K27me3, H3K4me3 ChIP-seq, and RNA-seq tracks at *Akt1* (**H**), *Mapkapk3* (**I**), and *Erbb2* (**J**), showing bivalent peaks and RNA-seq activation upon EED loss. (**K** and **L**) Averaged H3K27me3 and H3K4me3 ChIP-seq tracks at *Neurod1* (**K**) and *Hnf4a* (**L**), showing that *Neurod1*, but not *Hnf4a*, is bivalently marked. ****P < 0.0001. See also Supplemental Figure 11.

**Figure 8:**
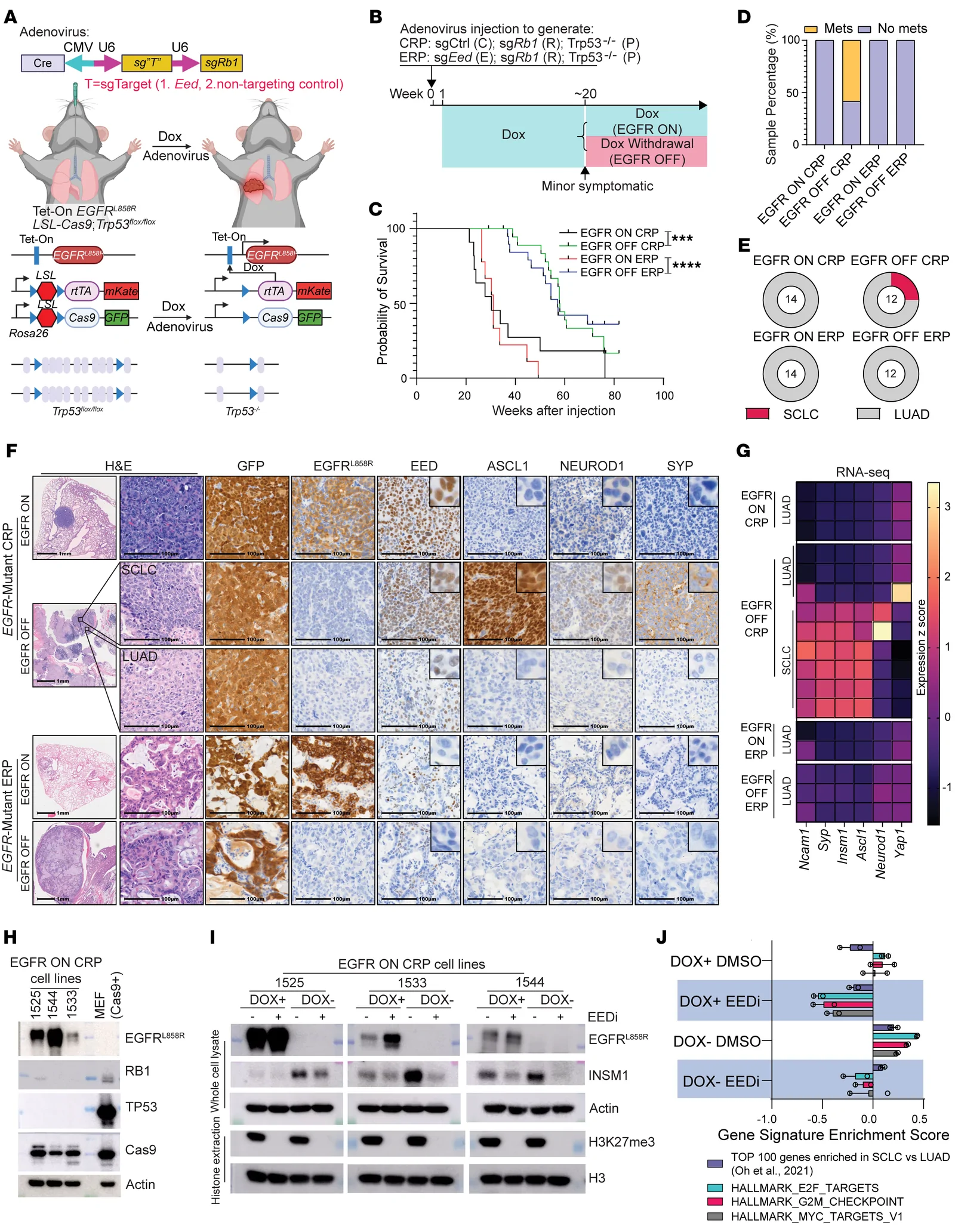
EED inactivation at tumor initiation prevents emergence of SCLC histology in an *EGFR*-mutant GEMM with *Rb1* and *Trp53* inactivation after EGFR withdrawal. (**A**) Schematic of CRISPR-based *EGFR*-mutant LUAD GEMM with *Rb1* and *Trp53* inactivation made isogenic for *Eed* using sg*Eed* to generate ERP tumors or nontargeting sgRNA to generate CRP tumors. (**B**) Timeline of adenovirus injection, Dox induction, and Dox withdrawal. (**C**) Kaplan-Meier survival estimate of mice injected with indicated adenoviruses and treated as in **B**. *P* values calculated by log-rank test. (**D**) Percentage of mice with metastases. (**E** and **F**) Tumor histology pie charts (**E**) and representative H&E and IHC (**F**) for GFP, EGFR L858R, EED, ASCL1, NEUROD1, and SYP from CRP EGFR ON tumors (*n* = 14, 2, 2, 2, 2, 2, and 2, respectively), CRP EGFR OFF tumors (*n* = 12, 4, 3, 4, 4, 4, and 3, respectively), ERP EGFR ON tumors (*n* = 14, 2, 2, 2, 2, 2, and 2, respectively), and ERP EGFR OFF tumors (*n* = 12, 3, 3, 3, 3, 3, and 3, respectively). Scale bars: 1 mm (first column), 100 μm (remaining columns). Insets, ×9 magnification. For **C**–**E**, *n* = 14 EGFR ON CRP, *n* = 12 EGFR OFF CRP, and *n* = 14 EGFR ON ERP mice. For **C**, *n* = 16 EGFR OFF ERP mice; for **D** and **E**, *n* = 12 EGFR OFF ERP mice because 4 did not develop tumors. (**G**) Heatmap of *z* scored SCLC neuroendocrine and non-neuroendocrine marker expression from RNA-seq. Each row is 1 tumor. Black, low; yellow, high. *n* = 3 EGFR ON CRP, *n* = 9 EGFR OFF CRP, *n* = 2 EGFR ON ERP, *n* = 3 EGFR OFF ERP tumors. (**H**) Immunoblot of EGFR ON CRP cell lines cultured in 1 μg/mL Dox. (**I**) Immunoblot of cell lines in **H** cultured with or without Dox and treated with DMSO or MAK-683, 2 μM, for 6 days. (**J**) Gene signature enrichment scores from RNA-seq of EGFR CRP 1525 cells under conditions in **I**. For **I**, representative immunoblots from > 3 biological replicates. For **J**, *n* = 2 biological replicates. CRP, sgControl; sg*Rb1*, *Trp53*−/−; ERP, sg*Eed*; sg*Rb1*, *Trp53*−/−; EGFR ON, Dox ON; EGFR OFF, Dox withdrawal. ***P < 0.001, ****P < 0.0001. See also Supplemental Figures 12 and 13.

**Figure 9:**
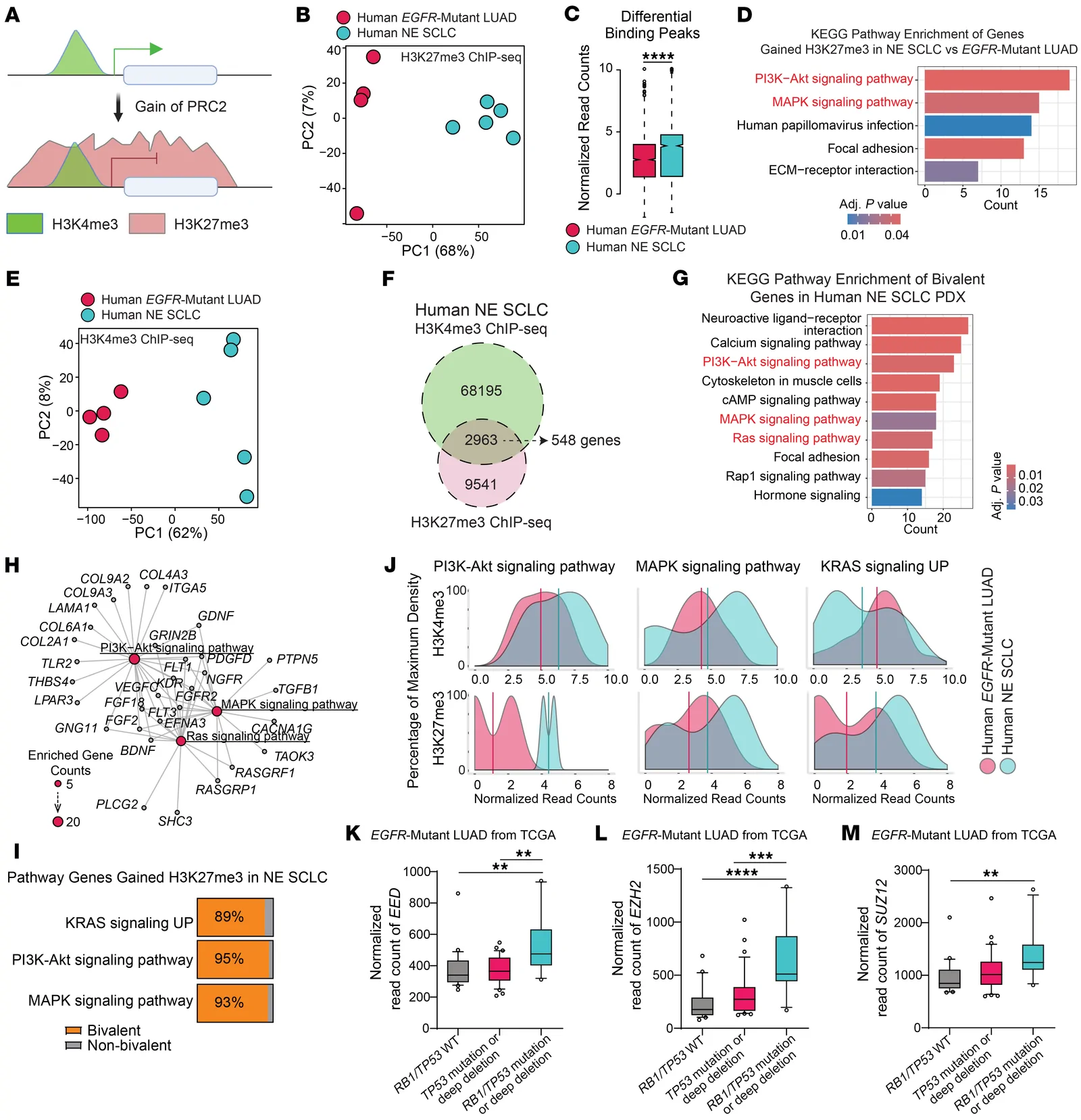
H3K27me3 represses LUAD oncogenic programs in human SCLC through bivalent silencing of RAS, PI3K, and MAPK pathway genes. (**A**) Schematic showing PRC2-mediated repression of genes with bivalent H3K4me3 and H3K27me3 marks. Figure created with BioRender. (**B** and **E**) PCA of H3K27me3 ChIP-seq (**B**) and H3K4me3 ChIP-seq (**E**) from 5 independent human de novo neuroendocrine (NE) SCLC PDX tumors and 4 independent human *EGFR*-mutant LUAD PDX tumors. (**C**) Box-and-whisker plots of normalized H3K27me3 ChIP-seq read counts in the indicated groups. Mann-Whitney *U* test was used to calculate 2-sided *P* value. (**D**) KEGG pathway enrichment of genes mapped to differential H3K27me3 peaks gained in neuroendocrine SCLC versus EGFR-mutant LUAD PDX tumors. (**F**) Schematic identifying bivalent genes marked by H3K4me3 and H3K27me3 in human neuroendocrine SCLC PDX tumors. (**G**) KEGG pathway enrichment of bivalent genes in human neuroendocrine SCLC PDX tumors. For **D** and **G**, bar color indicates BH-adjusted *P* value. Red, low; blue, high. Count: enriched gene count. (**H**) Gene network analysis of enriched RAS, PI3K-AKT, and MAPK pathways from **G**. Dot size indicates enriched gene count. (**I**) Bar plots showing the percentage of H3K4me3-marked genes in the indicated pathways that gained H3K27me3 in neuroendocrine SCLC PDX tumors and are therefore bivalent. (**J**) Density plots of normalized H3K4me3 and H3K27me3 ChIP-seq read counts mapped to genes in KRAS signaling UP, PI3K/AKT, and MAPK KEGG pathways in human neuroendocrine SCLC and *EGFR*-mutant LUAD PDX tumors. (**K**–**M**) RNA-seq batch-normalized expression of *EED* (**K**), *EZH2* (**L**), and *SUZ12* (**M**) in human *EGFR*-mutant LUAD tumors with indicated *RB1* and *TP53* alterations from The Cancer Genome Atlas (65). The box-and-whisker plots depict the 10th and 90th percentile (whiskers), the upper and lower quartiles, and the median. *RB1/TP53* WT, *n* = 22; *TP53* mutation or deep deletion, *n* = 34; *RB1/TP53* mutation or deep deletion, *n* = 10 tumors. One-way ANOVA with Tukey’s test was used to calculate 2-sided *P* values adjusted for multiple comparisons. **P < 0.01, ***P < 0.001, ****P < 0.0001. See also Supplemental Figure 14.

## Version 1 08/18/2026

In-Press Preview

## Version 2 10/15/2026

Electronic publication
