# Supplementary material for: Polycomb scaffolding subunit EED supports the SCLC neuroendocrine identity and EGFR-mutant LUAD-to-SCLC transformation
Source: J Clin Invest. 2026 Aug 18;136(20):e199454. doi: 10.1172/JCI199454 (PMC13668632; doi:10.1172/JCI199454)
Supplement: Supplementary file 1 [file jci-136-199454-s043.pdf]

Li et al. Supplemental Data

Li et al. Supplemental Figures

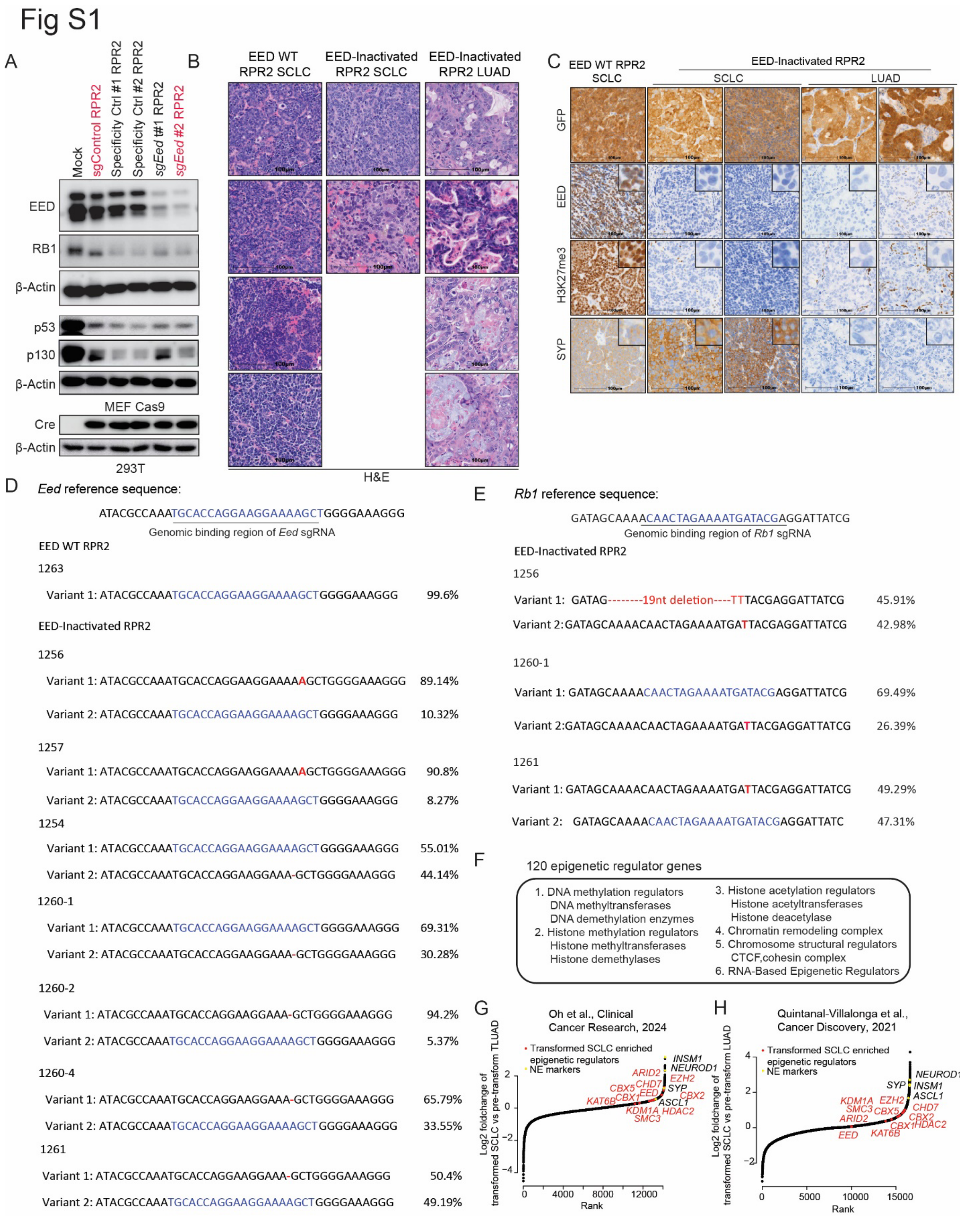
Fig S1
A
Mock
sgControl RPR2
Specificity Ctrl #1 RPR2
Specificity Ctrl #2 RPR2
sgEed #1 RPR2
sgEed #2 RPR2
EED
RB1
β-Actin
p53
p130
β-Actin
MEF Cas9
Cre
β-Actin
293T
B
EED WT RPR2 SCLC
EED-Inactivated RPR2 SCLC
EED-Inactivated RPR2 LUAD
H&E
C
EED WT RPR2 SCLC
EED-Inactivated RPR2
SCLC
LUAD
GFP
EED
H3K27me3
SYP
D
Eed reference sequence:
ATACGCCAAATGCACCAGGAAGGAAAAGCTGGGGAAAGGG
Genomic binding region of Eed sgRNA
EED WT RPR2
1263
Variant 1: ATACGCCAAATGCACCAGGAAGGAAAAGCTGGGGAAAGGG 99.6%
EED-Inactivated RPR2
1256
Variant 1: ATACGCCAAATGCACCAGGAAGGAAAAAGCTGGGGAAAGGG 89.14%
Variant 2: ATACGCCAAATGCACCAGGAAGGAAAAGCTGGGGAAAGGG 10.32%
1257
Variant 1: ATACGCCAAATGCACCAGGAAGGAAAAAGCTGGGGAAAGGG 90.8%
Variant 2: ATACGCCAAATGCACCAGGAAGGAAAAGCTGGGGAAAGGG 8.27%
1254
Variant 1: ATACGCCAAATGCACCAGGAAGGAAAAGCTGGGGAAAGGG 55.01%
Variant 2: ATACGCCAAATGCACCAGGAAGGAAA-GCTGGGGAAAGGG 44.14%
1260-1
Variant 1: ATACGCCAAATGCACCAGGAAGGAAAAGCTGGGGAAAGGG 69.31%
Variant 2: ATACGCCAAATGCACCAGGAAGGAAA-GCTGGGGAAAGGG 30.28%
1260-2
Variant 1: ATACGCCAAATGCACCAGGAAGGAAA-GCTGGGGAAAGGG 94.2%
Variant 2: ATACGCCAAATGCACCAGGAAGGAAAAGCTGGGGAAAGGG 5.37%
1260-4
Variant 1: ATACGCCAAATGCACCAGGAAGGAAA-GCTGGGGAAAGGG 65.79%
Variant 2: ATACGCCAAATGCACCAGGAAGGAAAAGCTGGGGAAAGGG 33.55%
1261
Variant 1: ATACGCCAAATGCACCAGGAAGGAAA-GCTGGGGAAAGGG 50.4%
Variant 2: ATACGCCAAATGCACCAGGAAGGAAAAGCTGGGGAAAGGG 49.19%
E
Rb1 reference sequence:
GATAGCAAAACAACTAGAAAATGATACGAGGATTATCG
Genomic binding region of Rb1 sgRNA
EED-Inactivated RPR2
1256
Variant 1: GATAG--------19nt deletion----TTTACGAGGATTATCG 45.91%
Variant 2: GATAGCAAAACAACTAGAAAATGATTACGAGGATTATCG 42.98%
1260-1
Variant 1: GATAGCAAAACAACTAGAAAATGATACGAGGATTATCG 69.49%
Variant 2: GATAGCAAAACAACTAGAAAATGATTACGAGGATTATCG 26.39%
1261
Variant 1: GATAGCAAAACAACTAGAAAATGATTACGAGGATTATCG 49.29%
Variant 2: GATAGCAAAACAACTAGAAAATGATACGAGGATTATC 47.31%
F
120 epigenetic regulator genes
1. DNA methylation regulators
DNA methyltransferases
DNA demethylation enzymes
2. Histone methylation regulators
Histone methyltransferases
Histone demethylases
3. Histone acetylation regulators
Histone acetyltransferases
Histone deacetylase
4. Chromatin remodeling complex
5. Chromosome structural regulators
CTCF,cohesin complex
6. RNA-Based Epigenetic Regulators
G
Oh et al., Clinical Cancer Research, 2024
Transformed SCLC enriched epigenetic regulators
NE markers
Log2 foldchange of transformed SCLC vs pre-transform TLUAD
Rank
INSM1
NEUROD1
EZH2
SYP
ARID2
CBX5
CHD7
CBX1
EED
KAT6B
CBX2
ASCL1
KDM1A
HDAC2
SMC3
H
Quintanal-Villalonga et al., Cancer Discovery, 2021
Transformed SCLC enriched epigenetic regulators
NE markers
Log2 foldchange of transformed SCLC vs pre-transform LUAD
Rank
NEUROD1
SYP
INSM1
ASCL1
KDM1A
EZH2
SMC3
CBX5
CHD7
ARID2
CBX2
CBX1
HDAC2
EED
KAT6B

Supplemental Figure 1. EED Isogenic RPR2 Adenovirus and SCLC GEMM Tumor Validation and Expression Analysis of Epigenetic Regulators in Human Transformed SCLC. Related to Figure 1.

(**A**) Immunoblot analysis of Cas9 expressing mouse embryonic fibroblast (MEF) cells and 293T cells infected with indicated RPR2 adenoviruses. Adenoviruses in red were used for intratracheal injections in LSL-Cas9 mice. (**B** and **C**) Representative H&E (**B**) and IHC for GFP, EED, H3K27me3, and Synaptophysin (SYP) (**C**) from additional EED-WT RPR2 SCLCs (n=11,2,2,2,2, respectively) and EED-Inactivated RPR2 LUADs (n=24,6,4,5,3, respectively) (see also Figure 1, D and E). Scale bar=100 microns. Insets are 9X magnification of the main image. (**D** and **E**) Sequence variants with percentages at the sgRNA binding region of endogenous *Eed* (**D**) and *Rb1* (**E**) from CRISPR amplicon sequencing of lung tumors from indicated genotypes. Variant nucleotides relative to the reference sequence are indicated in red text. (**F**) Categories of epigenetic regulators included in the 120 epigenetic regulator gene list (see Supplemental Table 1, Tab 3). (**G** and **H**) Rank plots of RNA-seq from Oh et al.(1) (**G**) and Quintanal-Villalonga et al.(2)(**H**) comparing transformed SCLC vs pre-transform LUAD. Red dots: *EED* and genes overlapped among the two above studies and 120 epigenetic regulator gene list. *EED* and *EZH2* ranked 12634 and 14120 of 14213 genes, respectively, in (**G**) and 10017 and 15837 of 16583 genes, respectively, in (**H**). Yellow dots: neuroendocrine genes validating SCLC transformation.

Fig. S2

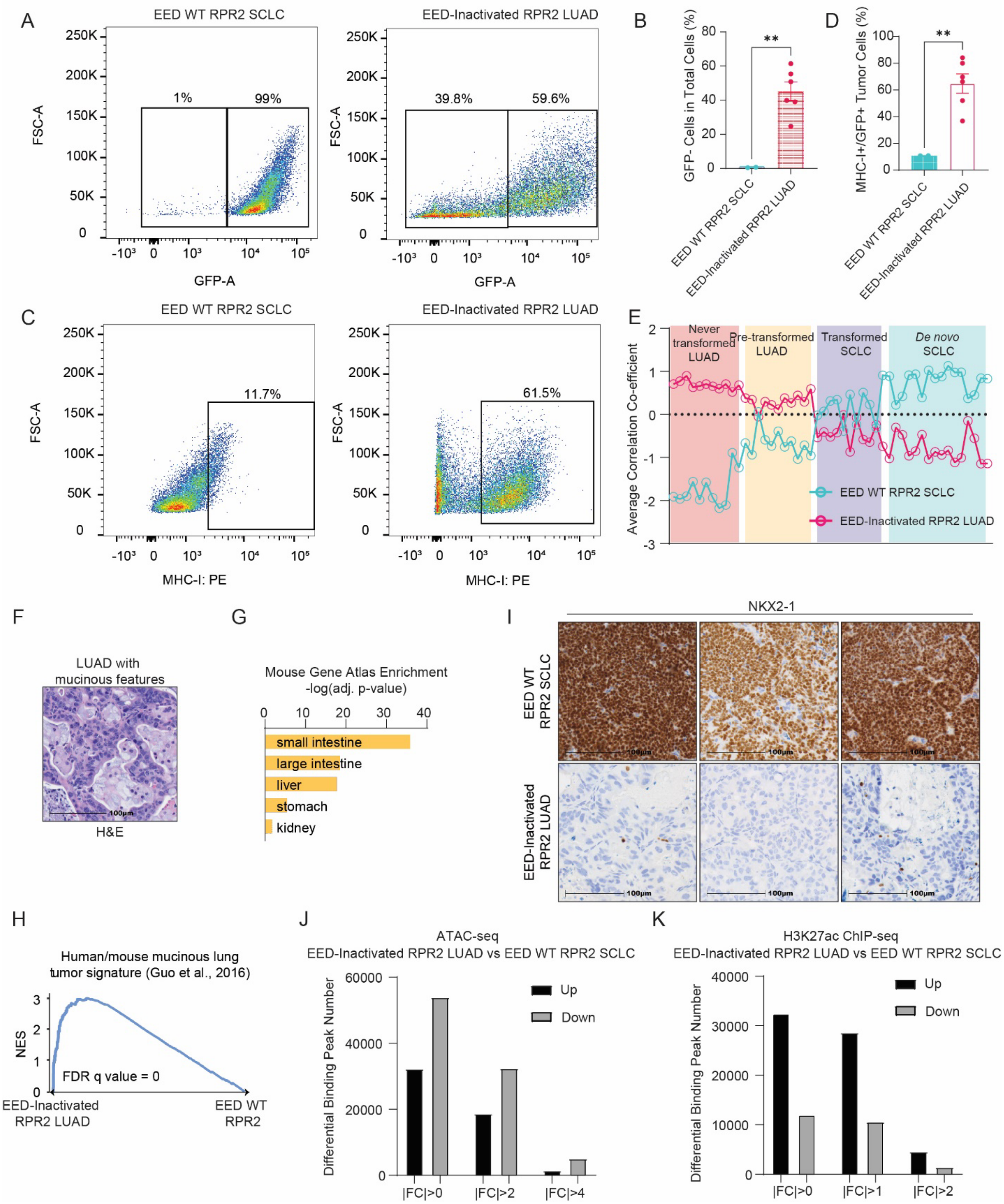

A
EED WT RPR2 SCLC
EED-Inactivated RPR2 LUAD
FSC-A
GFP-A
1%
99%
39.8%
59.6%
B
GFP- Cells in Total Cells (%)
**
EED WT RPR2 SCLC
EED-Inactivated RPR2 LUAD
D
MHC-I+/GFP+ Tumor Cells (%)
**
C
MHC-I: PE
11.7%
61.5%
E
Never transformed LUAD
Pre-transformed LUAD
Transformed SCLC
De novo SCLC
Average Correlation Co-efficient
EED WT RPR2 SCLC
EED-Inactivated RPR2 LUAD
F
LUAD with mucinous features
H&E
G
Mouse Gene Atlas Enrichment
-log(adj. p-value)
small intestine
large intestine
liver
stomach
kidney
I
NKX2-1
EED WT RPR2 SCLC
EED-Inactivated RPR2 LUAD
H
Human/mouse mucinous lung tumor signature (Guo et al., 2016)
NES
FDR q value = 0
EED-Inactivated RPR2 LUAD
EED WT RPR2
J
ATAC-seq
EED-Inactivated RPR2 LUAD vs EED WT RPR2 SCLC
Differential Binding Peak Number
Up
Down
|FC|>0
|FC|>2
|FC|>4
K
H3K27ac ChIP-seq
EED-Inactivated RPR2 LUAD vs EED WT RPR2 SCLC
Differential Binding Peak Number
Up
Down
|FC|>0
|FC|>1
|FC|>2

Supplemental Figure 2. EED Inactivation Epigenetically Promotes Lung Adenocarcinoma with Increased Restored MHC Class I Surface Expression and Increased Infiltration of Stromal and Immune Cells. Related to Figure 2.

(**A-B**) Representative scatter plots (**A**) and quantification (**B**) of flow cytometry analysis for GFP of EED-WT RPR2 SCLC and EED-Inactivated RPR2 LUAD mouse tumors. GFP marks tumor cells and hence increased GFP negative cells in *EED*-Inactivated *RPR2* LUAD mouse tumors indicates an increase in stromal/immune cells within tumors. (**C** and **D**) Representative scatter plots (**C**) and quantification (**D**) of flow cytometry analysis for cell surface MHC class I on GFP-positive tumor cells. For (**B**) and (**D**), n= 2 independent tumors for EED-WT RPR2 SCLC and n=6 independent tumors for EED-Inactivated RPR2 LUAD. Two-sided p-values were calculated using a Student's t test. *=p<0.05, **=p<0.01, ***=p<0.001, ****=p<0.0001. (**E**) Dot plot of the average correlation coefficient between transcriptomic profiles of RPR2 tumors and human lung tumors with different histologies from Quintanal-Villalonga et al. 2021 (2). (**F**) Representative H&E image of EED-Inactivated RPR2 LUAD with mucinous features (n=36). Scale bar=100 microns. (**G**) Bar plot of negative log transformed adjusted p values of top organs from the mouse gene atlas whose transcriptomic profile enriched in differentially upregulated genes in EED-Inactivated RPR2 LUAD vs. EED-WT RPR2 SCLC tumors with an adjusted p-value <0.05. (**H**) Enrichment plot of human/mouse mucinous lung tumor signature from Guo et al. 2017 (3) comparing EED-Inactivated RPR2 LUAD vs EED-WT RPR2 SCLC. (**I**) IHC for NKX2-1 from tumors from 3 individual mice in the indicated groups. Scale bar=100 microns. Insets are 9X magnification of the main image. (**J** and **K**) Bar plots of differential binding peak numbers from ATAC-seq (**J**) and H3K27ac ChIP-seq (**K**) comparing EED-WT RPR2 SCLC and EED-Inactivated RPR2 LUAD mouse tumors at the fold change (FC) thresholds indicated.

Fig. S3

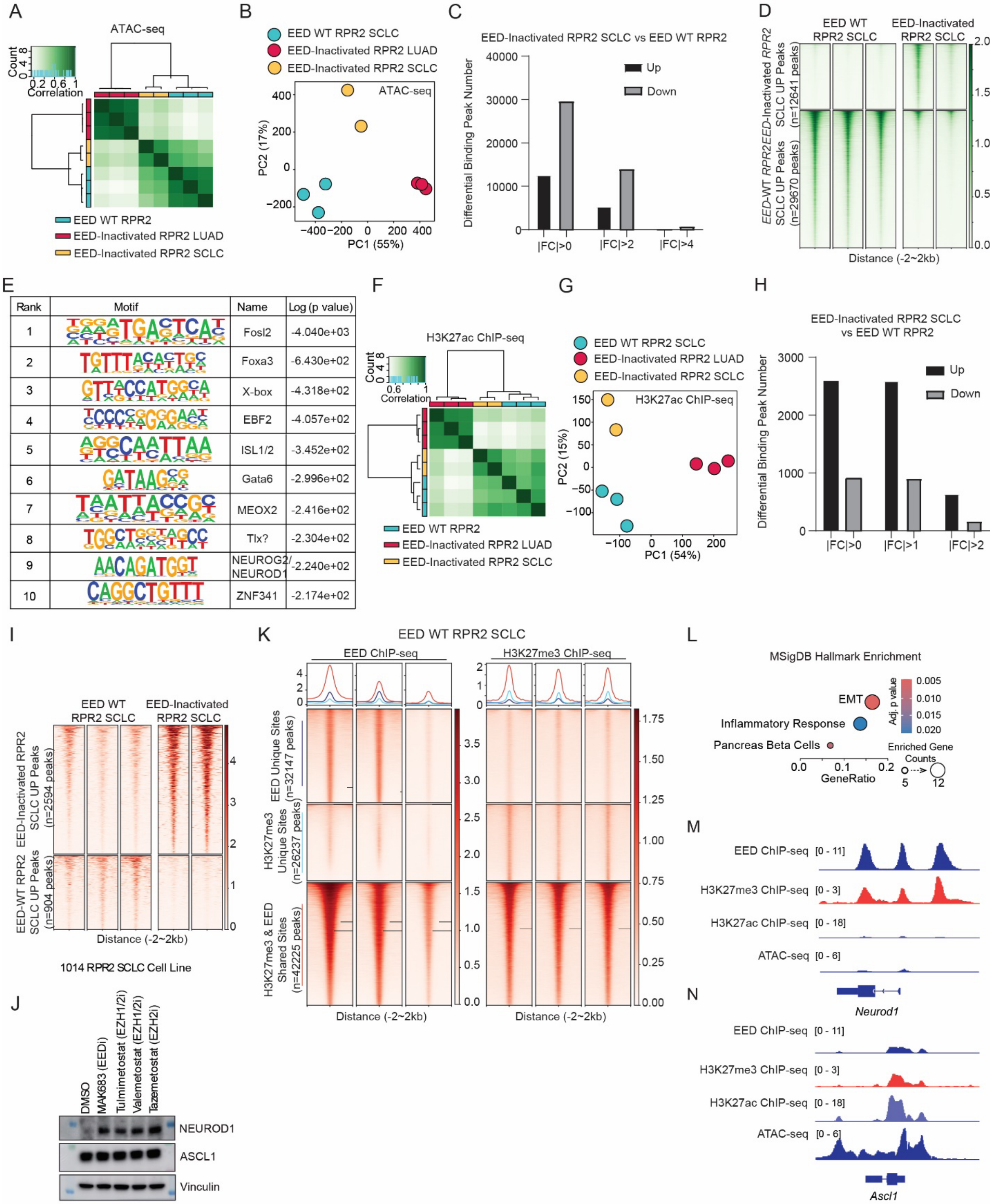
A
ATAC-seq
Count
Correlation
EED WT RPR2
EED-Inactivated RPR2 LUAD
EED-Inactivated RPR2 SCLC
B
EED WT RPR2 SCLC
EED-Inactivated RPR2 LUAD
EED-Inactivated RPR2 SCLC
ATAC-seq
PC2 (17%)
PC1 (55%)
C
EED-Inactivated RPR2 SCLC vs EED WT RPR2
Differential Binding Peak Number
Up
Down
|FC|>0
|FC|>2
|FC|>4
D
EED WT RPR2 SCLC
EED-Inactivated RPR2 SCLC
EED-WT RPR2 SCLC UP Peaks (n=29670 peaks)
EED-Inactivated RPR2 SCLC UP Peaks (n=12641 peaks)
Distance (-2~2kb)
E
Rank | Motif | Name | Log (p value)
1 | Fosl2 | -4.040e+03
2 | Foxa3 | -6.430e+02
3 | X-box | -4.318e+02
4 | EBF2 | -4.057e+02
5 | ISL1/2 | -3.452e+02
6 | Gata6 | -2.996e+02
7 | MEOX2 | -2.416e+02
8 | Tlx? | -2.304e+02
9 | NEUROG2/NEUROD1 | -2.240e+02
10 | ZNF341 | -2.174e+02
F
H3K27ac ChIP-seq
Count
Correlation
EED WT RPR2
EED-Inactivated RPR2 LUAD
EED-Inactivated RPR2 SCLC
G
EED WT RPR2 SCLC
EED-Inactivated RPR2 LUAD
EED-Inactivated RPR2 SCLC
H3K27ac ChIP-seq
PC2 (15%)
PC1 (54%)
H
EED-Inactivated RPR2 SCLC vs EED WT RPR2
Differential Binding Peak Number
Up
Down
|FC|>0
|FC|>1
|FC|>2
I
EED WT RPR2 SCLC
EED-Inactivated RPR2 SCLC
EED-Inactivated RPR2 SCLC UP Peaks (n=2594 peaks)
EED-WT RPR2 SCLC UP Peaks (n=904 peaks)
Distance (-2~2kb)
J
1014 RPR2 SCLC Cell Line
DMSO
MAK683 (EEDi)
Tulmimetostat (EZH1/2i)
Valemetostat (EZH1/2i)
Tazemetostat (EZH2i)
NEUROD1
ASCL1
Vinculin
K
EED WT RPR2 SCLC
EED ChIP-seq
H3K27me3 ChIP-seq
EED Unique Sites (n=32147 peaks)
H3K27me3 Unique Sites (n=26237 peaks)
H3K27me3 & EED Shared Sites (n=42225 peaks)
Distance (-2~2kb)
L
MSigDB Hallmark Enrichment
EMT
Inflammatory Response
Pancreas Beta Cells
GeneRatio
Adj. p value
Enriched Gene Counts
M
EED ChIP-seq [0 - 11]
H3K27me3 ChIP-seq [0 - 3]
H3K27ac ChIP-seq [0 - 18]
ATAC-seq [0 - 6]
Neurod1
N
EED ChIP-seq [0 - 11]
H3K27me3 ChIP-seq [0 - 3]
H3K27ac ChIP-seq [0 - 18]
ATAC-seq [0 - 6]
Ascl1

Supplemental Figure 3. Epigenetic Changes after *EED*-Inactivated RPR2 SCLCs. Related to Figure 3.

(**A** and **B**) Correlation plot (**A**) and PCA plot (**B**) of all ATAC-seq data from EED-Inactivated LUAD, EED-Inactivated SCLC, and EED-WT SCLC lung tumors. (**C**) Differential peak numbers of ATAC-seq comparing EED-Inactivated SCLC vs EED-WT SCLC. (**D** and **I**) Heatmaps of differential binding peaks from ATAC-seq (**D**) and H3K27ac ChIP-seq (**I**) comparing 2 independent EED-Inactivated RPR2 SCLC vs. 3 independent EED-WT RPR2 SCLC tumors. (**E**) HOMER motif enrichment analysis of top enriched motifs in EED-Inactivated SCLC vs EED-WT SCLC. (**F** and **G**) Correlation plot (**F**) and PCA plot (**G**) all H3K27Ac ChIP-seq data from EED-Inactivated LUAD, EED-Inactivated SCLC, and EED-WT SCLC lung tumors. (**H**) Differential peak numbers of H3K27Ac ChIP-seq comparing EED-Inactivated SCLC vs EED-WT SCLC. (**J**) Immunoblot analysis of 1014 RPR2 EED-WT SCLC cells treated with the EZH1/2 or EED inhibitors indicated. MAK683 (EED inhibitor)=2 μM, Tulmimetostat (EZH1/2 inhibitor)=100 nM, Valemetostat (EZH1/2 inhibitor)=100 nM, Tazemetostat (EZH2 inhibitor)=5 μM. (**K**) Heatmaps and profile plots of shared binding peaks of EED and H3K27me3 (red) and unique peaks to EED (purple) and H3K27me3 (blue). White is low, red is high. (**L**) Dot plots of hallmark pathway enrichment analysis of direct functional EED targets in EED-Inactivated SCLC. BH adjusted p values were visualized by the dot color, red=low, blue=high. Enriched gene counts were visualized by the size of the dots. EMT: Epithelial to mesenchymal transition. NES: Normalized enrichment score. (**M** and **N**) Tracks of averaged EED, H3K27me3, and H3K27ac ChIP-seq and ATAC-seq data across all samples (n=3) from the EED-WT SCLC mouse lung tumors at *Neurod1* (**M**) *and Ascl1* (**N**). FC=Fold Change. For correlation plots, green is high, white is low.

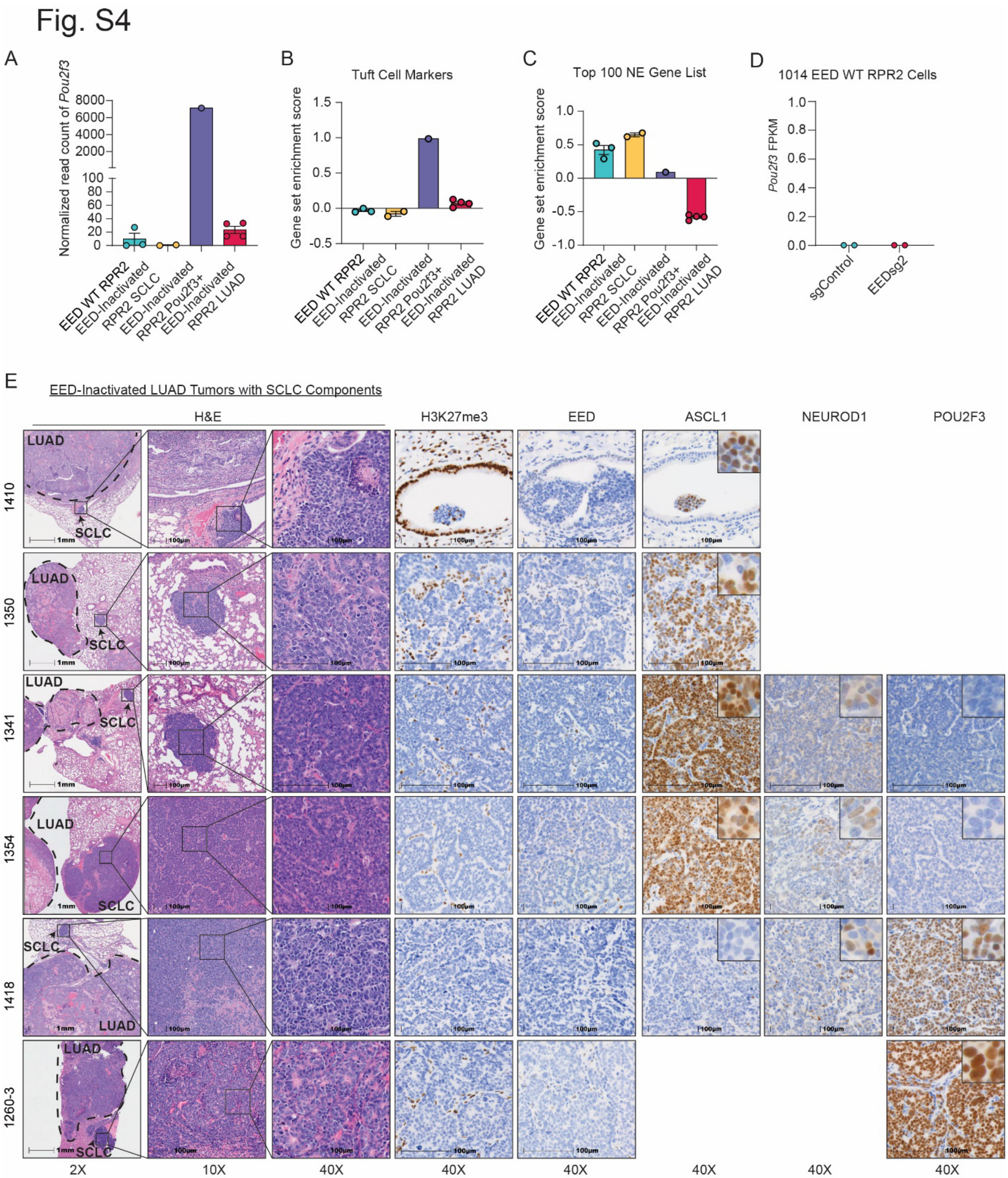

Fig. S4
A
Normalized read count of Pou2f3
EED WT RPR2
EED-Inactivated RPR2 SCLC
EED-Inactivated RPR2 Pou2f3+
EED-Inactivated RPR2 LUAD
B
Tuft Cell Markers
Gene set enrichment score
C
Top 100 NE Gene List
Gene set enrichment score
D
1014 EED WT RPR2 Cells
Pou2f3 FPKM
sgControl
EEDsg2
E
EED-Inactivated LUAD Tumors with SCLC Components
H&E
H3K27me3
EED
ASCL1
NEUROD1
POU2F3
1410
1350
1341
1354
1418
1260-3
LUAD
SCLC
2X
10X
40X

Supplemental Figure 4. Analysis of SCLC Components in EED-Inactivated RPR2 LUAD Reveals Loss of EED and Heterogenous Expression of SCLC Lineage Transcription Factors. Related to Figure 4.

(**A-C**) Bar plots of normalized read counts of *Pou2f3* (**A**), gene set enrichment scores of tuft cell markers (**B**), and top 100 neuroendocrine genes from RNA-seq data from Figures 2-3 from the GEMM lung tumors indicated. Student's t-test was used to calculate two-sided p values. *=p<0.05, **=p<0.01, ***=p<0.001, ****=p<0.0001. (**D**) Dot plot of *Pou2f3* FPKM values from RNA-seq data (see Figure 3J) from 1014 RPR2 EED-WT SCLC cells transduced with an sgRNA targeting *EED* (sg*Eed*) or a non-targeting sgRNA as a control (sgControl). (**E**) Representative H&E and IHC staining for H3K27me3, EED, ASCL1, NEUROD1, and POU2F3 from EED-Inactivated RPR2 LUAD lung tumors that also contained a SCLC component shown in Figure 1C. The 2nd and 3rd columns show serial magnifications of the outlined regions in the corresponding left column. Columns 3-8 represent serial sections of the same SCLC nodules at the same magnification. Scale bar (1st column) =1 mm. Scale bar (2nd-8th columns) =100 microns. Insets are 9X magnification of the corresponding main image. Blank slots are present because SCLC tumor nodule was lost when sectioning paraffin block. Dotted line in 1st column indicates LUAD or SCLC compartment. 2X,10X, and 40X magnification of images are indicated. Of note, the residual H3K27me3 staining in sporadic single cells within the nodule in tumor 1410 likely represents immune cells; however, this could not be confirmed because the nodule was lost during serial sectioning.

Fig. S5

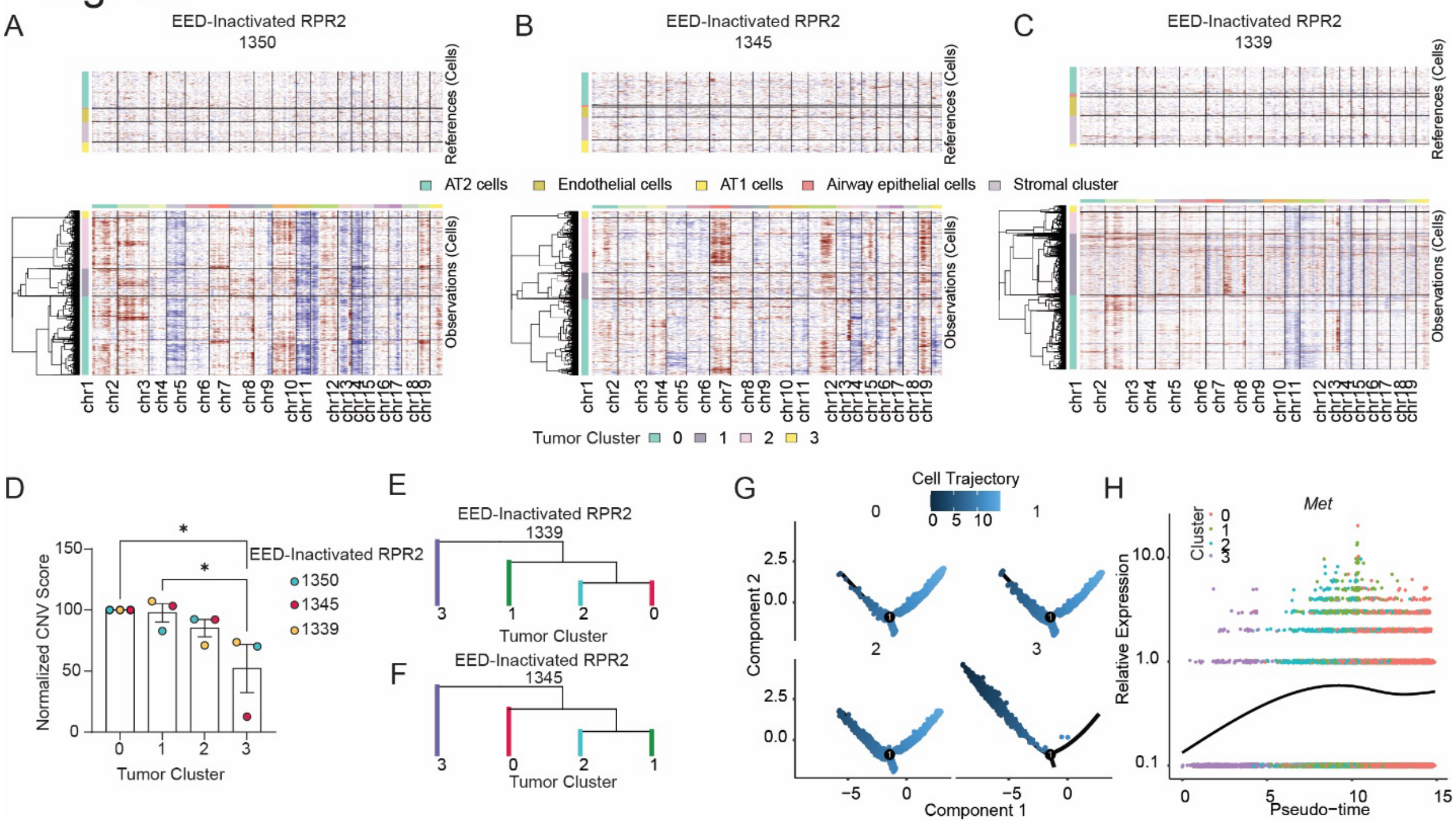

A
EED-Inactivated RPR2
1350
B
EED-Inactivated RPR2
1345
C
EED-Inactivated RPR2
1339
References (Cells)
Observations (Cells)
AT2 cells
Endothelial cells
AT1 cells
Airway epithelial cells
Stromal cluster
chr1 chr2 chr3 chr4 chr5 chr6 chr7 chr8 chr9 chr10 chr11 chr12 chr13 chr14 chr15 chr16 chr17 chr18 chr19
Tumor Cluster 0 1 2 3
D
Normalized CNV Score
Tumor Cluster
EED-Inactivated RPR2
1350
1345
1339
E
EED-Inactivated RPR2
1339
Tumor Cluster
F
EED-Inactivated RPR2
1345
Tumor Cluster
G
Cell Trajectory
Component 1
Component 2
H
Met
Cluster
Relative Expression
Pseudo-time

Supplemental Figure 5. EED Inactivation Promotes LUAD Histology Likely Through a NEUROD1-Positive Intermediate*.* Related to Figure 4.

(**A-C**) The landscape of copy number variations (CNVs) from snRNA-seq from each EED-Inactivated RPR2 LUAD tumor indicated. Cells within each tumor cluster are grouped by hierarchical clustering based on predicted CNVs. Pulmonary epithelium/stroma cells within each tumor are used as a normal reference. For reference cells, n=1037 in EED-Inactivated RPR2 LUAD (1350 tumor), n=2529 in EED-Inactivated RPR2 LUAD (1345 tumor), n=904 in EED-Inactivated RPR2 LUAD (1339 tumor). For tumor cells: n=3256 in EED-Inactivated RPR2 LUAD (1350), n=5015 in EED-Inactivated RPR2 LUAD (1345), n=8319 in EED-Inactivated RPR2 LUAD (1339). (**D**) Quantification of CNV events (normalized CNV score) from each tumor clusters in each EED-Inactivated RPR2 LUAD sample. Two-sided p-values were calculated using a student's t-test. *=$p<0.05$, **=$p<0.01$, ***=$p<0.001$, ****=$p<0.0001$. (**E** and **F**) Phylogenetic trees of tumor clusters using Maximum Parsimony method from EED-Inactivated RPR2 LUAD tumors 1339 (**E**) and 1345 (**F**). (**G**) Pseudo-time cell trajectory projection of each separate tumor cell clusters. Black=early, blue=late. (**H**) Scatter plot of *Met* relative expression in pseudo-time with each tumor cell cluster indicated. Curve indicates average expression.

Fig. S6

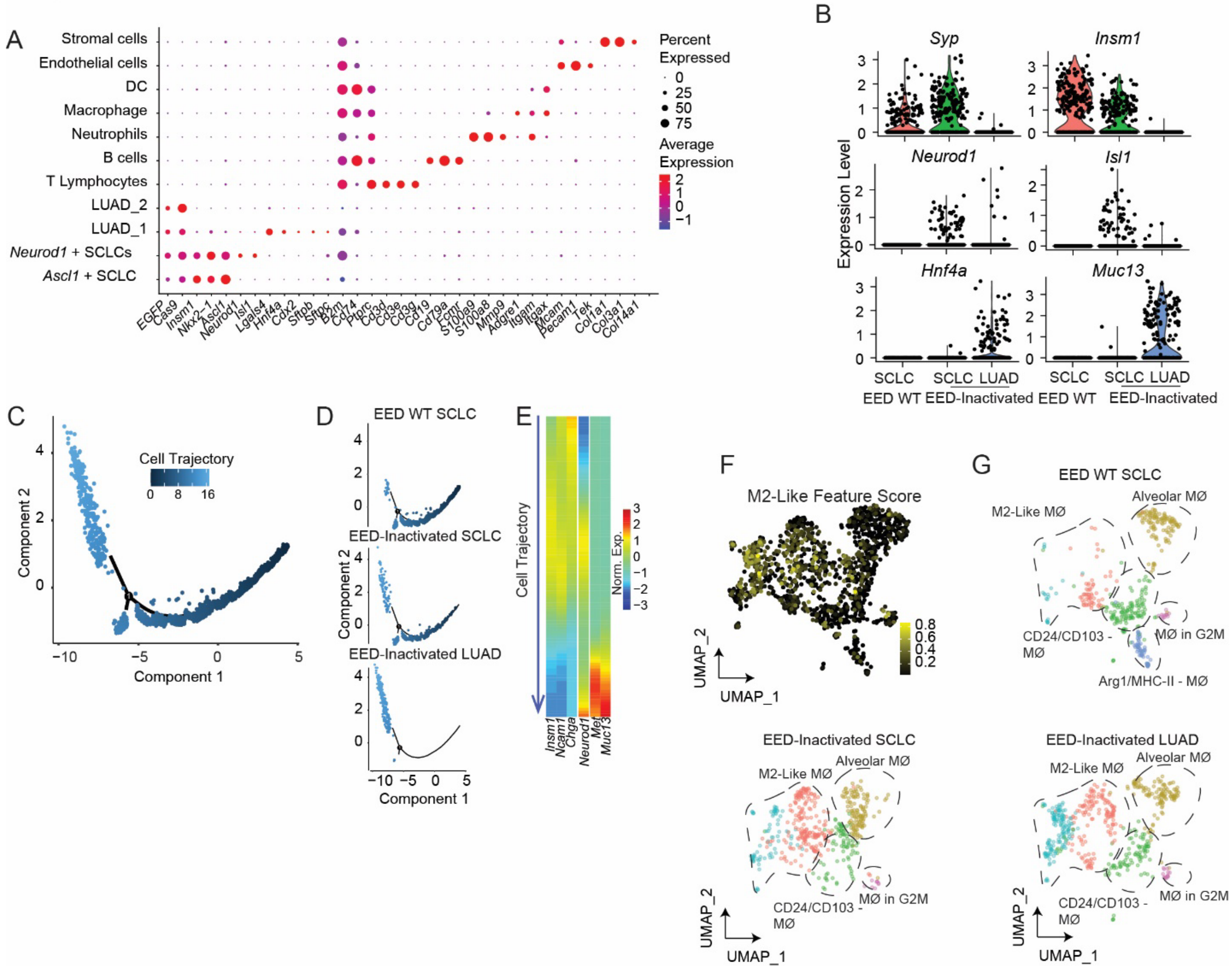
A
Stromal cells
Endothelial cells
DC
Macrophage
Neutrophils
B cells
T Lymphocytes
LUAD_2
LUAD_1
Neurod1 + SCLCs
Ascl1 + SCLC
Percent Expressed
Average Expression
B
Syp
Insm1
Neurod1
Isl1
Hnf4a
Muc13
Expression Level
SCLC
SCLC LUAD
EED WT
EED-Inactivated
C
Cell Trajectory
Component 1
Component 2
D
EED WT SCLC
EED-Inactivated SCLC
EED-Inactivated LUAD
E
Cell Trajectory
Norm. Exp.
Insm1
Ncam1
Chga
Neurod1
Met
Muc13
F
M2-Like Feature Score
UMAP_1
UMAP_2
G
EED WT SCLC
EED-Inactivated SCLC
EED-Inactivated LUAD
M2-Like MØ
Alveolar MØ
CD24/CD103 - MØ
MØ in G2M
Arg1/MHC-II - MØ

Supplemental Figure 6. LUAD Histology after EED Inactivation Requires Cues from the Tumor Immune Microenvironment. Related to Figure 5.

(**A**) Expression dot plot of genes defining cell types in the scRNA-seq analysis. Expression level is visualized as dot color. Blue is low, red is high. Percent of cells expressing the indicated marker is visualized by the size of the dot. (**B**) Violin plots of marker gene expression from scRNA-seq data in each sample group indicated. (**C** and **D**) Pseudo-time tumor cell trajectory projection of 3 independent EED-WT SCLC, 2 independent EED-Inactivated SCLC, and 4 independent EED-Inactivated LUAD tumors in all samples (**C**) and each group separated (**D**). Black=early, blue=late. (**E**) Heatmap of normalized expression level of marker genes along the tumor cell trajectory. Blue is low, red is high. (**F** and **G**) UMAP of Mϕ cells from scRNA-seq with color gradients indicating expression level of M2-like feature genes (**F**) and subcluster labeling in indicated sample groups (**G**). See also in Supplemental Table 5, Tab 4. Black is low, yellow is high. Mϕ, macrophage.

Figure S7

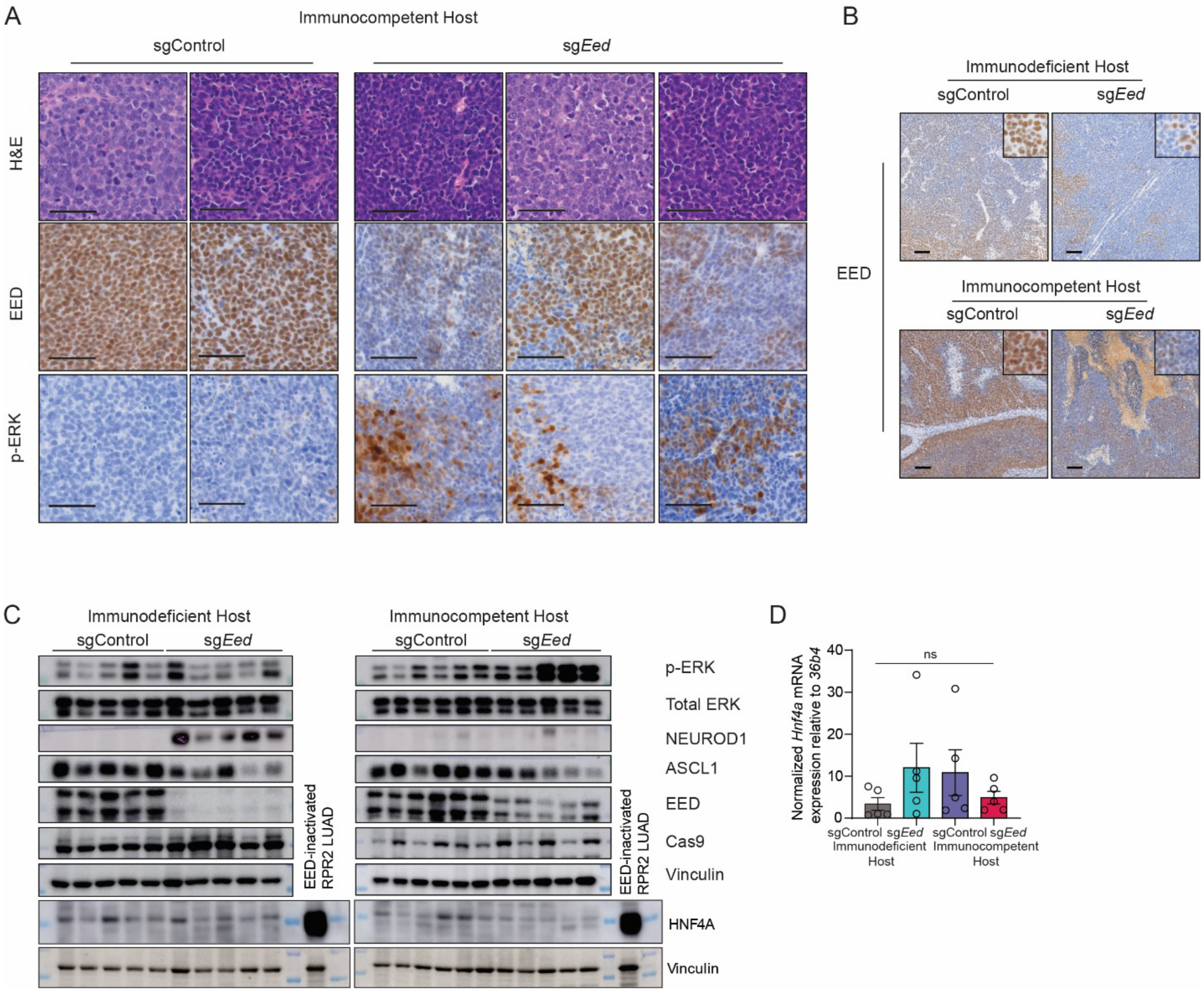
A
Immunocompetent Host
sgControl
sgEed
H&E
EED
p-ERK
B
Immunodeficient Host
sgControl
sgEed
EED
Immunocompetent Host
sgControl
sgEed
C
Immunodeficient Host
sgControl
sgEed
Immunocompetent Host
sgControl
sgEed
p-ERK
Total ERK
NEUROD1
ASCL1
EED
Cas9
Vinculin
EED-inactivated RPR2 LUAD
HNF4A
Vinculin
D
Normalized Hnf4a mRNA expression relative to 36b4
ns
40
30
20
10
0
sgControl sgEed
Immunodeficient Host
sgControl sgEed
Immunocompetent Host

Supplemental Figure 7. Phospho-ERK Activation in EED-Inactivated Transplanted SCLC Allografts in Immunocompetent BL6J Mice. Related to Figure 5.
(**A**) Representative H&E and IHC staining for EED and p-ERK from 1014 RPR2 EED-WT SCLC cells transduced with an sgRNA targeting *Eed* (sg*Eed*) (n=5,5,5, respectively) or a non-targeting sgRNA as a control (sgControl) (n=2,2,2, respectively) and then subcutaneously implanted in immunocompetent (BL6J) mice (see also Figure 5C). Scale bar is 50 microns. (**B**) Low magnification of representative IHC of EED from sgControl and sg*Eed* 1014 tumors subcutaneously implanted in immunodeficient (NCr nude) (n=3,3, respectively) or immunocompetent (BL6J) (n=2,5, respectively) mice showing stronger selection to restore EED in immunocompetent vs. immunodeficient mice. Insets are high magnification (40X) of the same tumor. Scale bar is 100 microns. (**C** and **D**) Immunoblot analysis (**C**) and RT-qPCR analysis (**D**) of 1014 RPR2 EED-WT SCLC cells transduced with an sgRNA targeting *Eed* (sg*Eed*) or a non-targeting sgRNA as a control (sgControl) and then subcutaneously implanted in immunodeficient (NCr nude) or immunocompetent (BL6J) mice. In (**C**) P-ERK, total ERK, NEUROD1, ASCL1, EED, Cas9, and Vinculin blots are the same blots from Figure 5C, but now HNF4A is included using the same lysates as above with an EED-Inactivated RPR2 LUAD GEMM tumor as a positive control. Two-sided p-values were calculated using one-way ANOVA followed by Tukey test for multiple comparison adjustment. ns=p>0.05.

Fig. S8

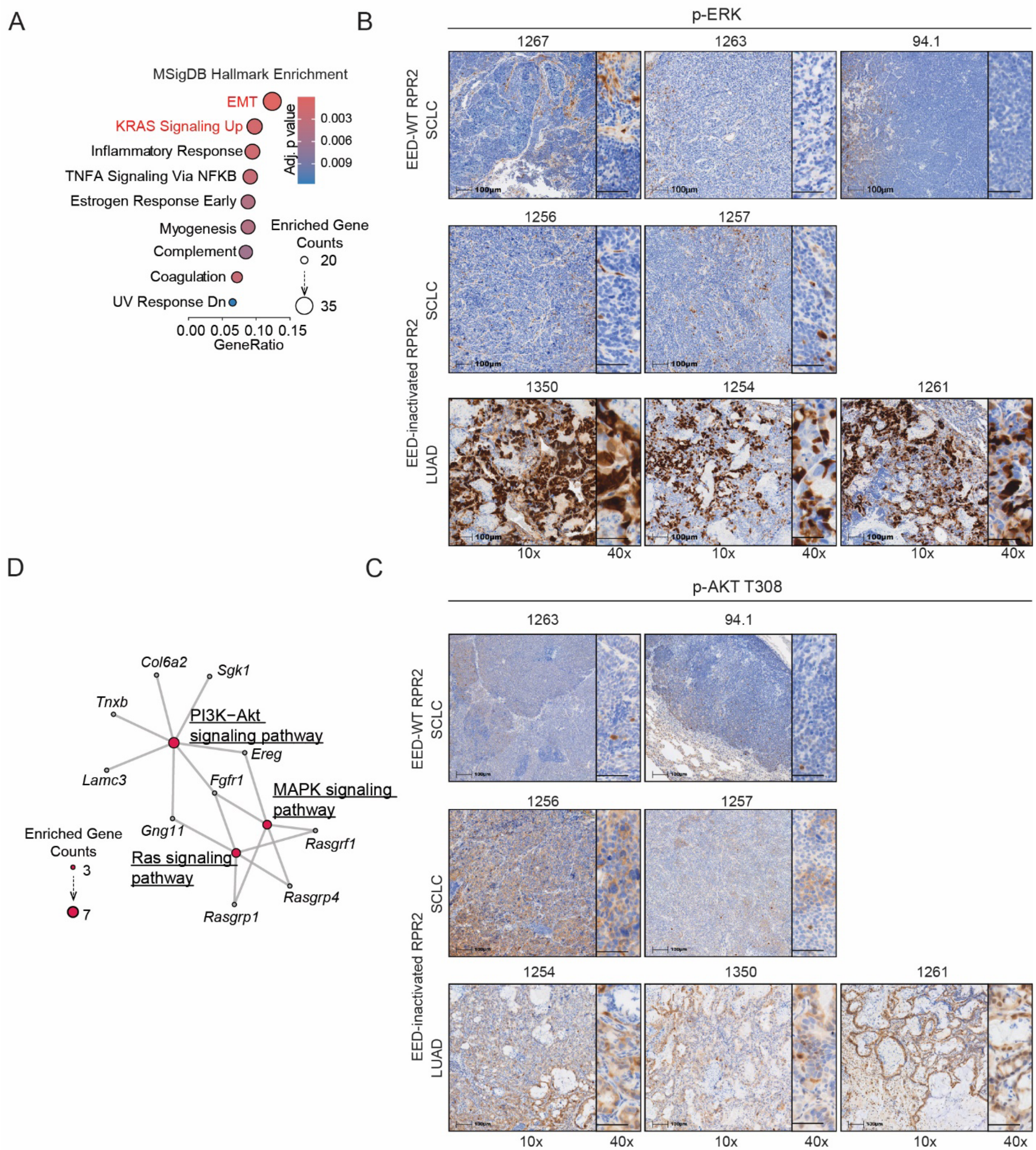
A
MSigDB Hallmark Enrichment
EMT
KRAS Signaling Up
Inflammatory Response
TNFA Signaling Via NFKB
Estrogen Response Early
Myogenesis
Complement
Coagulation
UV Response Dn
0.00 0.05 0.10 0.15
GeneRatio
Adj. p value
0.003
0.006
0.009
Enriched Gene Counts
20
35
B
p-ERK
1267
1263
94.1
EED-WT RPR2 SCLC
1256
1257
EED-inactivated RPR2
SCLC
1350
1254
1261
LUAD
10x
40x
D
Col6a2
Sgk1
Tnxb
PI3K−Akt signaling pathway
Ereg
Lamc3
Fgfr1
MAPK signaling pathway
Enriched Gene Counts
Gng11
Rasgrf1
3
Ras signaling pathway
Rasgrp4
7
Rasgrp1
C
p-AKT T308
1263
94.1
EED-WT RPR2 SCLC
1256
1257
EED-inactivated RPR2
SCLC
1254
1350
1261
LUAD
10x
40x

Supplemental Figure 8. Activation of MAPK and PI3K Pathways in EED-Inactivated LUADs. Related to Figure 6.
(**A**) Hallmark pathway enrichment analysis of direct functional EED targets in EED-Inactivated LUAD. EMT: Epithelial to mesenchymal transition. BH adjusted p values were visualized by the dot color, red=low, blue=high. Enriched gene counts were visualized by the size of the dots. (**B** and **C**) Representative IHC staining for p-ERK (**B**) and p-AKT T308 (**C**) from EED-WT RPR2 SCLC (n=3,2, respectively), EED-Inactivated RPR2 SCLC (n=2,2, respectively), and EED-Inactivated RPR2 LUAD (n=3,3, respectively) lung tumors. The bottom positioned 10X, and 40X are magnifications of the corresponding image columns. Scale bar is 100 microns. (**D**) Gene network analysis of enriched Ras, AKT, and MAPK pathways of overlapping EED direct functional targets in EED-Inactivated RPR2 SCLC and EED-Inactivated RPR2 LUAD. Enriched gene counts were visualized by the size of the dots.

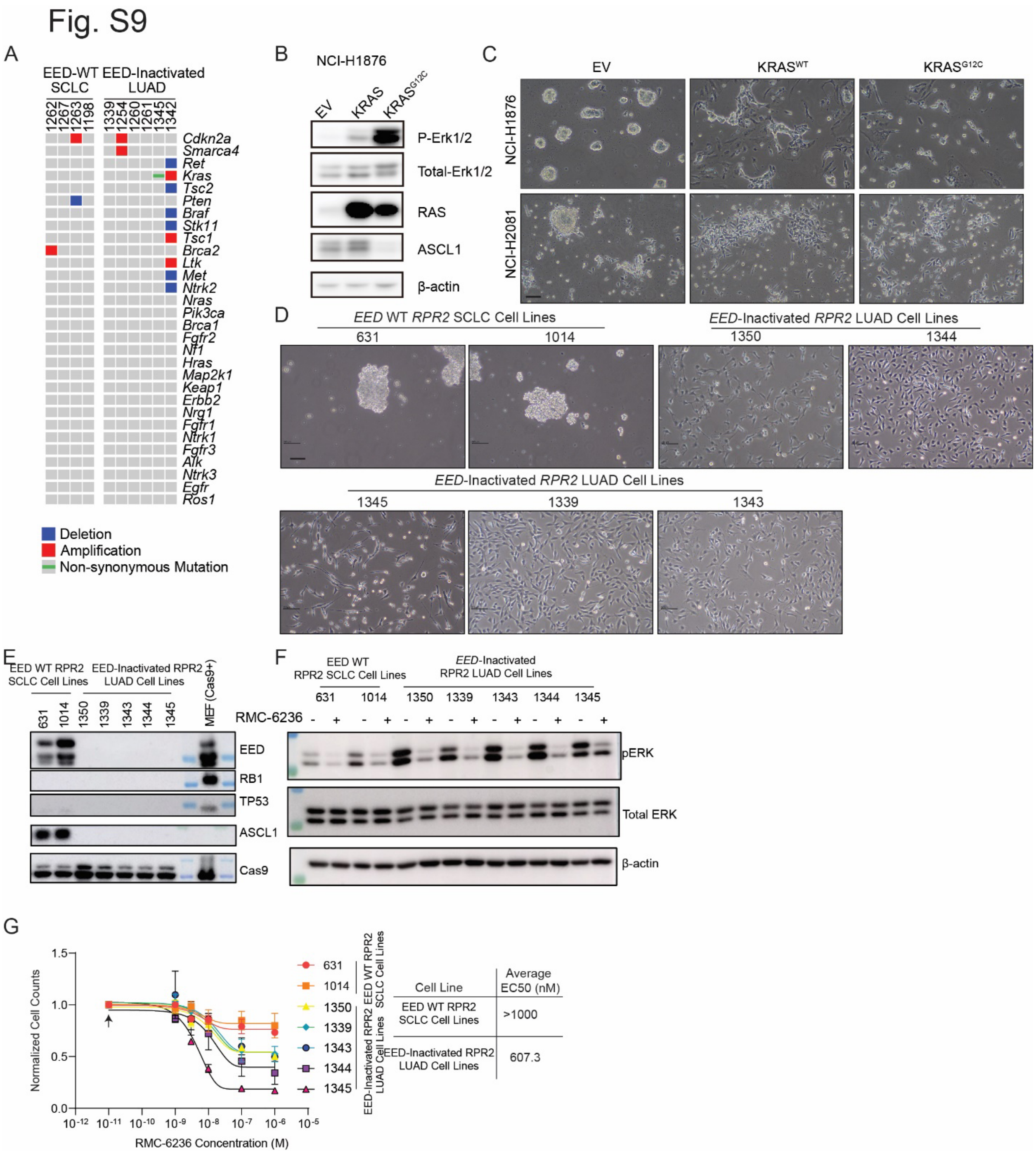
Fig. S9
A
EED-WT SCLC
EED-Inactivated LUAD
1262 1267 1263 1198
1339 1254 1260 1261 1345 1342
Cdkn2a
Smarca4
Ret
Kras
Tsc2
Pten
Braf
Stk11
Tsc1
Brca2
Ltk
Met
Ntrk2
Nras
Pik3ca
Brca1
Fgfr2
Nf1
Hras
Map2k1
Keap1
Erbb2
Nrg1
Fgfr1
Ntrk1
Fgfr3
Alk
Ntrk3
Egfr
Ros1
Deletion
Amplification
Non-synonymous Mutation
B
NCI-H1876
EV
KRAS
KRASG12C
P-Erk1/2
Total-Erk1/2
RAS
ASCL1
β-actin
C
EV
KRASWT
KRASG12C
NCI-H1876
NCI-H2081
D
EED WT RPR2 SCLC Cell Lines
631
1014
EED-Inactivated RPR2 LUAD Cell Lines
1350
1344
EED-Inactivated RPR2 LUAD Cell Lines
1345
1339
1343
E
EED WT RPR2 SCLC Cell Lines
EED-Inactivated RPR2 LUAD Cell Lines
631 1014 1350 1339 1343 1344 1345 MEF (Cas9+)
EED
RB1
TP53
ASCL1
Cas9
F
EED WT RPR2 SCLC Cell Lines
EED-Inactivated RPR2 LUAD Cell Lines
631 1014 1350 1339 1343 1344 1345
RMC-6236 - + - + - + - + - + - + - +
pERK
Total ERK
β-actin
G
Normalized Cell Counts
1.5
1.0
0.5
0.0
10-12 10-11 10-10 10-9 10-8 10-7 10-6 10-5
RMC-6236 Concentration (M)
631
1014
1350
1339
1343
1344
1345
EED WT RPR2 SCLC Cell Lines
EED-Inactivated RPR2 LUAD Cell Lines
Cell Line
Average EC50 (nM)
EED WT RPR2 SCLC Cell Lines
>1000
EED-Inactivated RPR2 LUAD Cell Lines
607.3

Supplemental Figure 9. EED-Inactivated RPR2 LUAD Cell Lines have Increased MAPK Activity and are Relatively Hypersensitive to RAS and MAPK Inhibition. Related to Figure 6.

(**A**) Oncoprint plot showing genomic alterations of LUAD oncogenic drivers in EED-WT RPR2 (n=4) and EED-Inactivated RPR2 tumors (n=6). Each tumor is from an individual mouse. Tumor 1345 has a missense mutation of $KRAS^{G12D}$ with allele frequency of 0.203. (**B**) Immunoblot analysis of the human NCI-H1876 SCLC cell line lentivirally transduced to overexpress $KRAS^{WT}$, constitutively activated $KRAS^{G12C}$, or the corresponding empty vector (EV). (**C**) Representative micrographs of NCI-H1876 and NCI-H2081 cells overexpressing the indicated constructs. Scale bar=50 microns. (**D**) Representative micrographs of cell lines made from EED-WT RPR2 lung tumors (631, 1014) and cell lines made from EED-Inactivated RPR2 LUAD tumors (1350, 1344, 1345, 1339, and 1343). Scale bar=100 microns. (**E**) Immunoblot analysis of cell lines in D confirming loss of EED and ASCL1 in EED-Inactivated RPR2 LUAD tumors and loss of RB1 and TP53 in all cell lines. Cas9 expressing mouse embryonic fibroblasts (MEFs) were used as control. (**F**) Immunoblot analysis of cell lines from E treated overnight at 1 micromolar with RAS-ON inhibitor RMC-6236. (**G**) Normalized dose response curves with EC50 indicated of each cell line from E treated with RMC-6236. If 50% killing were not reached at 1000 nM, 1000 nM was used in calculating the average EC50. Arrows **G** indicate DMSO-treated sample used for normalization. n=2 independent experiments.

Fig. S10

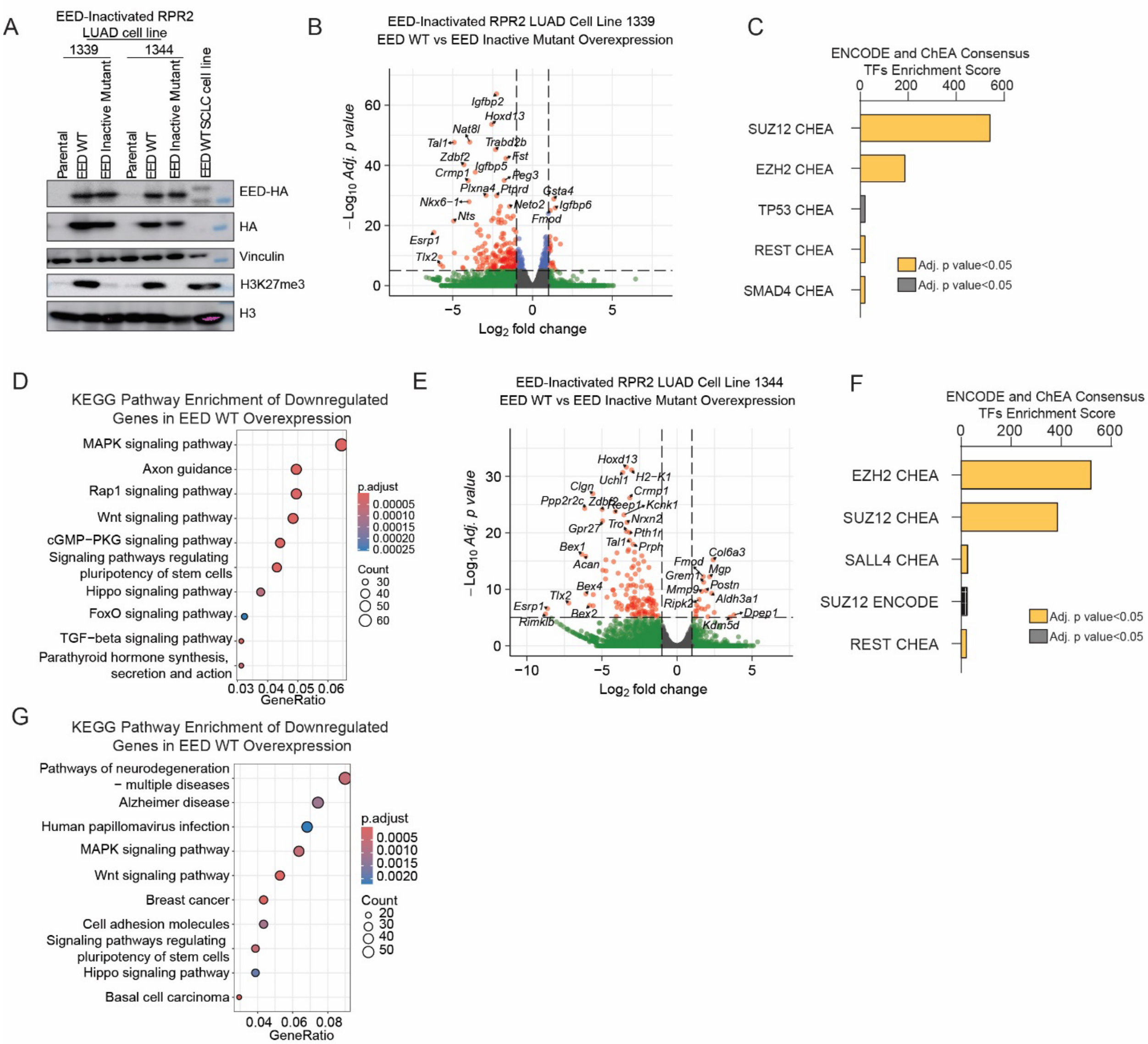
A
EED-Inactivated RPR2 LUAD cell line
1339
1344
Parental
EED WT
EED Inactive Mutant
Parental
EED WT
EED Inactive Mutant
EED WT SCLC cell line
EED-HA
HA
Vinculin
H3K27me3
H3
B
EED-Inactivated RPR2 LUAD Cell Line 1339
EED WT vs EED Inactive Mutant Overexpression
−Log10 Adj. p value
Log2 fold change
C
ENCODE and ChEA Consensus TFs Enrichment Score
SUZ12 CHEA
EZH2 CHEA
TP53 CHEA
REST CHEA
SMAD4 CHEA
Adj. p value<0.05
Adj. p value<0.05
D
KEGG Pathway Enrichment of Downregulated Genes in EED WT Overexpression
MAPK signaling pathway
Axon guidance
Rap1 signaling pathway
Wnt signaling pathway
cGMP−PKG signaling pathway
Signaling pathways regulating pluripotency of stem cells
Hippo signaling pathway
FoxO signaling pathway
TGF−beta signaling pathway
Parathyroid hormone synthesis, secretion and action
p.adjust
Count
GeneRatio
E
EED-Inactivated RPR2 LUAD Cell Line 1344
EED WT vs EED Inactive Mutant Overexpression
−Log10 Adj. p value
Log2 fold change
F
ENCODE and ChEA Consensus TFs Enrichment Score
EZH2 CHEA
SUZ12 CHEA
SALL4 CHEA
SUZ12 ENCODE
REST CHEA
Adj. p value<0.05
Adj. p value<0.05
G
KEGG Pathway Enrichment of Downregulated Genes in EED WT Overexpression
Pathways of neurodegeneration − multiple diseases
Alzheimer disease
Human papillomavirus infection
MAPK signaling pathway
Wnt signaling pathway
Breast cancer
Cell adhesion molecules
Signaling pathways regulating pluripotency of stem cells
Hippo signaling pathway
Basal cell carcinoma
p.adjust
Count
GeneRatio

Supplemental Figure 10. EED Overexpression Represses RAS/MAPK Signaling. Related to Figure 6.

(**A**) Immunoblots of EED-Inactivated RPR2 LUAD cell lines (1339 and 1344) lentivirally transduced to re-express EED-WT or an EED catalytic dead mutant (EED Inactive Mutant) which fails to restore H3K27me3 relative to EED-WT. EED-WT SCLC RPR2 cell line (1014 cells) are included as a benchmark control. (**B** and **E**) Volcano plots of RNA-seq data comparing EED-WT vs. EED inactive mutant in the EED-Inactivated RPR2 LUAD cell lines 1339 (**B**) and 1344 (**E**). n=2 biological independent experiments for each condition for each cell line. (**C** and **F**) Bar plot of ENCODE and ChEA Consensus TFs enrichment score from top enriched transcription factors whose targets were enriched in the differentially downregulated genes in RNA-seq data from (**B**), (**E**) comparing EED-WT vs EED inactive mutant in EED-Inactivated RPR2 LUAD cell lines 1339 (**C**) and 1344 (**F**). Yellow: significantly enriched with adjusted p-value<0.05, grey: adjusted p-value>0.05. (**D** and **G**) Dot plots of KEGG pathway enrichment analysis of significantly downregulated genes in EED-WT vs EED inactive mutant in EED-Inactivated RPR2 LUAD cell lines 1339 (**D**) and 1344 (**G**). BH adjusted p-values were visualized by the dot color, red=low, blue=high. Enriched gene counts were visualized by the size of the dots. Gene ratio=enriched gene number/total differentially downregulated genes.

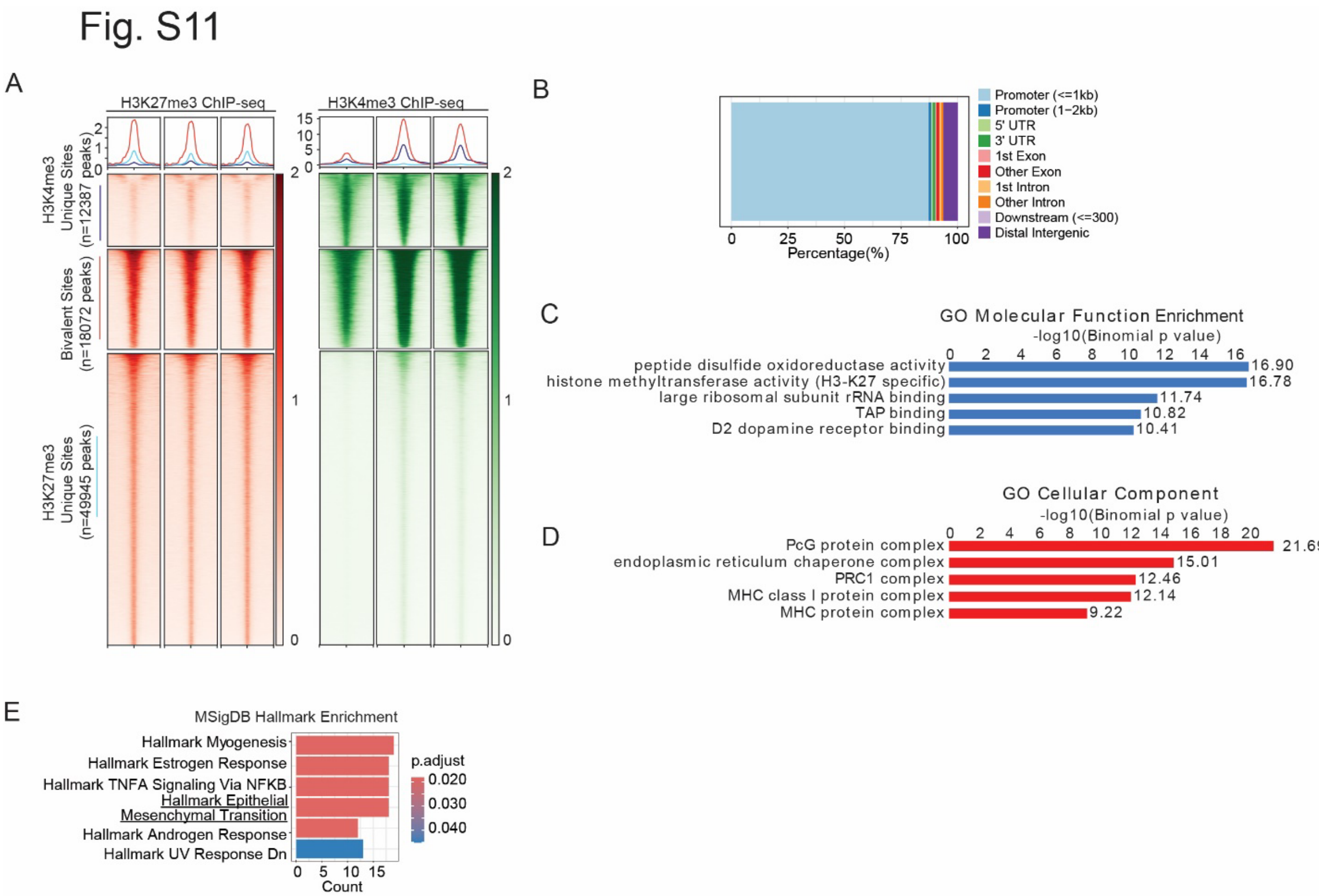

Fig. S11
A
H3K27me3 ChIP-seq
H3K4me3 ChIP-seq
H3K4me3 Unique Sites (n=12387 peaks)
Bivalent Sites (n=18072 peaks)
H3K27me3 Unique Sites (n=49945 peaks)
B
Promoter (<=1kb)
Promoter (1−2kb)
5' UTR
3' UTR
1st Exon
Other Exon
1st Intron
Other Intron
Downstream (<=300)
Distal Intergenic
Percentage(%)
C
GO Molecular Function Enrichment
-log10(Binomial p value)
peptide disulfide oxidoreductase activity 16.90
histone methyltransferase activity (H3-K27 specific) 16.78
large ribosomal subunit rRNA binding 11.74
TAP binding 10.82
D2 dopamine receptor binding 10.41
D
GO Cellular Component
-log10(Binomial p value)
PcG protein complex 21.69
endoplasmic reticulum chaperone complex 15.01
PRC1 complex 12.46
MHC class I protein complex 12.14
MHC protein complex 9.22
E
MSigDB Hallmark Enrichment
Hallmark Myogenesis
Hallmark Estrogen Response
Hallmark TNFA Signaling Via NFKB
Hallmark Epithelial Mesenchymal Transition
Hallmark Androgen Response
Hallmark UV Response Dn
p.adjust
0.020
0.030
0.040
Count

Supplemental Figure 11. LUAD Histology after PRC2 Loss Is Associated with Reactivation of Bivalently Marked RAS/PI3K/MAPK Pathway Genes and NEUROD1. Related to Figure 7. (**A**) Heatmaps and profile plots of shared binding peaks of H3K27me3 and H3K4me3 (red) and unique peaks to H3K4me3 (purple) and H3K27me3 (blue) in murine EED-WT RPR2 SCLC lung tumors. (**B**) Genomic distribution of bivalent peaks around the gene body. (**C** and **D**) Genomic Regions Enrichment of Annotations Tool (GREAT) analysis of bivalent peaks for gene ontology molecular function enrichment (**C**) and cellular component enrichment (**D**). Binomial p-values are indicated. (**E**) Bar plot of hallmark pathway enrichment of genes with bivalent peaks in its promoter region (absolute distance <2 kb from TSS) in EED-WT RPR2 SCLC. BH adjusted p-values were visualized by the bar color, red=low, blue=high. Count: Enriched gene counts.

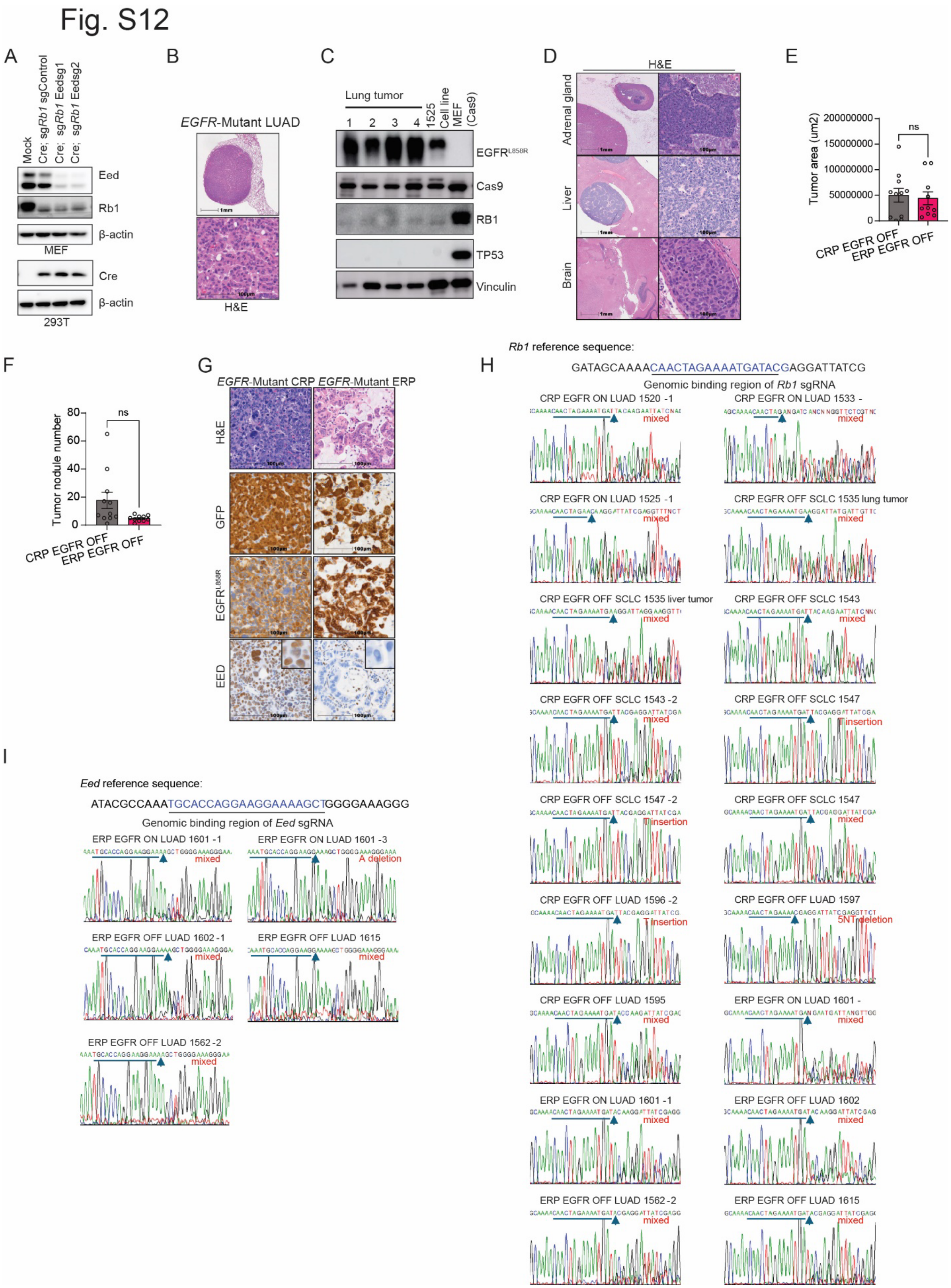
Fig. S12
A
Mock
Cre; sgRb1 sgControl
Cre; sgRb1 Eedsg1
Cre; sgRb1 Eedsg2
Eed
Rb1
β-actin
MEF
Cre
β-actin
293T
B
EGFR-Mutant LUAD
H&E
C
Lung tumor
1 2 3 4
1525 Cell line
MEF (Cas9)
EGFRL858R
Cas9
RB1
TP53
Vinculin
D
H&E
Adrenal gland
Liver
Brain
E
Tumor area (um2)
ns
CRP EGFR OFF
ERP EGFR OFF
F
Tumor nodule number
ns
CRP EGFR OFF
ERP EGFR OFF
G
EGFR-Mutant CRP
EGFR-Mutant ERP
H&E
GFP
EGFRL858R
EED
H
Rb1 reference sequence:
GATAGCAAAACAACTAGAAAATGATACGAGGATTATCG
Genomic binding region of Rb1 sgRNA
CRP EGFR ON LUAD 1520 -1
CRP EGFR ON LUAD 1533 -
CRP EGFR ON LUAD 1525 -1
CRP EGFR OFF SCLC 1535 lung tumor
CRP EGFR OFF SCLC 1535 liver tumor
CRP EGFR OFF SCLC 1543
CRP EGFR OFF SCLC 1543 -2
CRP EGFR OFF SCLC 1547
CRP EGFR OFF SCLC 1547 -2
CRP EGFR OFF SCLC 1547
CRP EGFR OFF LUAD 1596 -2
CRP EGFR OFF LUAD 1597
CRP EGFR OFF LUAD 1595
CRP EGFR ON LUAD 1601 -
ERP EGFR ON LUAD 1601 -1
ERP EGFR OFF LUAD 1602
ERP EGFR OFF LUAD 1562 -2
ERP EGFR OFF LUAD 1615
mixed
insertion
5NT deletion
I
Eed reference sequence:
ATACGCCAAATGCACCAGGAAGGAAAAGCTGGGGAAAGGG
Genomic binding region of Eed sgRNA
ERP EGFR ON LUAD 1601 -1
ERP EGFR ON LUAD 1601 -3
ERP EGFR OFF LUAD 1602 -1
ERP EGFR OFF LUAD 1615
ERP EGFR OFF LUAD 1562 -2
mixed
A deletion

Supplemental Figure 12. EED Inactivation at Tumor Initiation Prevents Emergence of SCLC Histology in an *EGFR*-Mutant GEMM with *Rb1* and *Trp53* Inactivation after EGFR Withdrawal. Related to Figure 8.
(**A**) Immunoblot analysis of Cas9 expressing mouse embryonic fibroblast (MEF) cells and 293T cells transduced with indicated adenoviruses. (**B**) Representative H&E of a representative *EGFR*-Mutant lung tumor (n=12) transduced with CMV-cre;sgControl;sg*Rb1* adenovirus and treated with Dox (Doxycycline) until the mouse became symptomatic from tumor burden. Scale bar (top =1 mm, scale bar (bottom)=100 microns. (**C**) Immunoblot analysis of lung tumors and a derived cell line from *EGFR*-Mutant sgControl sgRb1 Trp53-/- (CRP) mice treated with Dox until mice became clinically symptomatic from lung tumor burden in Dox ON mouse. Cas9 expressing MEFs were used as control. (**D**) Representative H&E images of distant metastasis in adrenal gland, liver, and brain from *EGFR*-Mutant sgControl, sg*Rb1, Trp53*-/- (CRP) with tumor recurrence after Dox withdrawal according to the treatment strategy in Figure 8B. Scale bar (left) = 1 mm, scale bar (right)=100 microns. (**E** and **F**) Quantification of tumor area (**E**) and tumor nodule number (**F**) of endpoint tumors in EGFR OFF CRP and ERP mice from Figure 8C. (**G**) Representative H&E and IHC staining for GFP, $EGFR^{L858R}$, and EED from lung tumors from *EGFR*-Mutant CRP (n=14,2,2,2, respectively) and ERP mice (n=14,2,2,2, respectively) treated with Dox. Scale bar=100 microns. Insets are 9X magnification of the main image. (**H** and **I**) Chromatograms from Sanger sequencing after PCR amplification of the DNA sequence around *Rb1* (**H**) and *Eed* (**I**) guide RNA binding regions. Blue arrows indicate sgRNA cut site. Red text indicates type of CRISPR-mediated edits. Student's t-test was used to calculate two-sided p-values. *=p<0.05, **=p<0.01, ***=p<0.001, ****=p<0.0001. CRP: sgControl; sg*Rb1*; *Trp53*-/-, ERP: sg*Eed*; sg*Rb1*; *Trp53*-/-. EGFR ON=Dox ON, EGFR OFF=Dox withdrawal.

Fig. S13

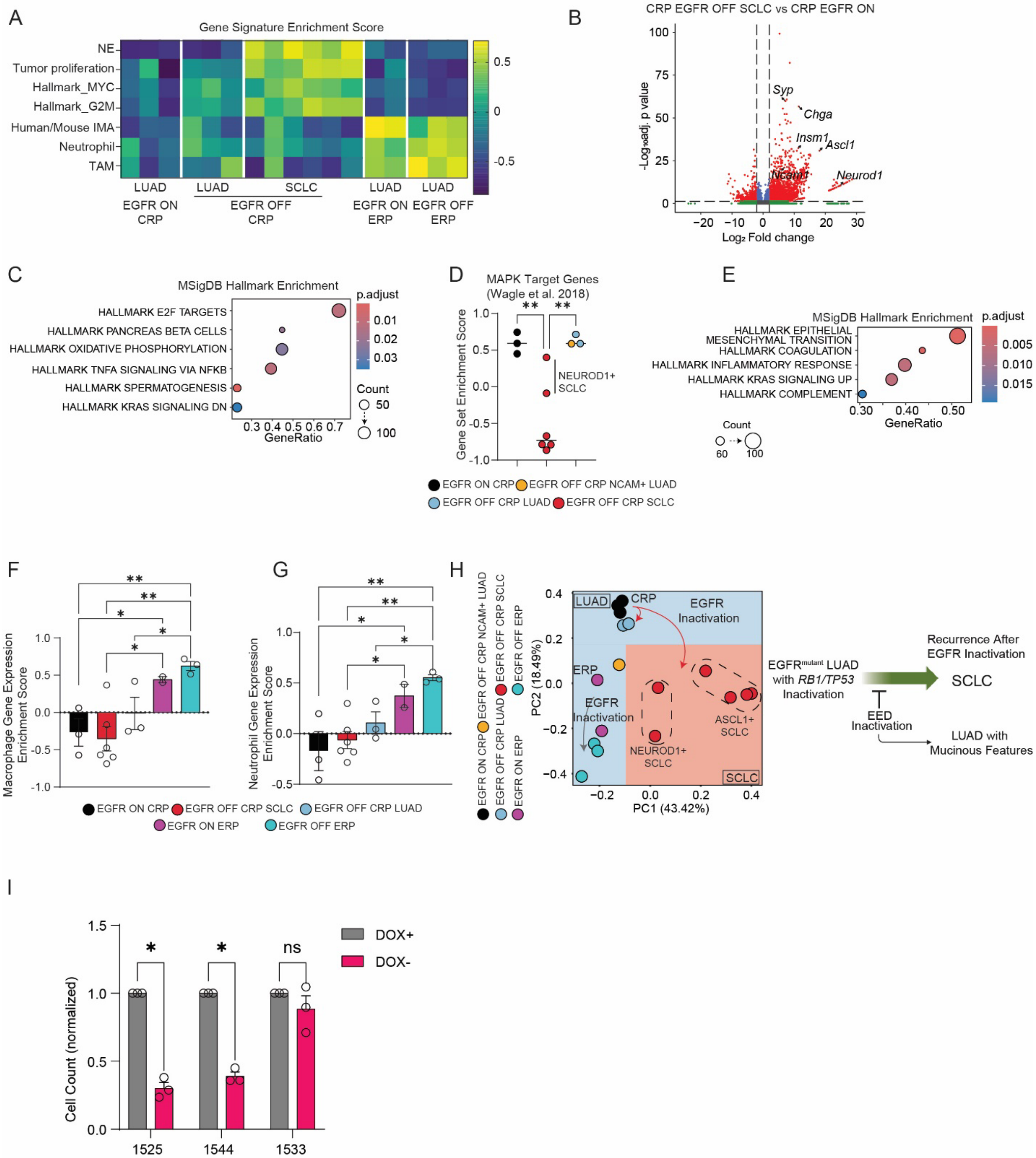
A
Gene Signature Enrichment Score
NE
Tumor proliferation
Hallmark_MYC
Hallmark_G2M
Human/Mouse IMA
Neutrophil
TAM
LUAD
EGFR ON CRP
LUAD SCLC
EGFR OFF CRP
LUAD
EGFR ON ERP
LUAD
EGFR OFF ERP
B
CRP EGFR OFF SCLC vs CRP EGFR ON
Syp
Chga
Insm1
Ascl1
Ncam1
Neurod1
Log2 Fold change
C
MSigDB Hallmark Enrichment
HALLMARK E2F TARGETS
HALLMARK PANCREAS BETA CELLS
HALLMARK OXIDATIVE PHOSPHORYLATION
HALLMARK TNFA SIGNALING VIA NFKB
HALLMARK SPERMATOGENESIS
HALLMARK KRAS SIGNALING DN
GeneRatio
p.adjust
Count
D
MAPK Target Genes
(Wagle et al. 2018)
Gene Set Enrichment Score
NEUROD1+ SCLC
EGFR ON CRP
EGFR OFF CRP NCAM+ LUAD
EGFR OFF CRP LUAD
EGFR OFF CRP SCLC
E
MSigDB Hallmark Enrichment
HALLMARK EPITHELIAL MESENCHYMAL TRANSITION
HALLMARK COAGULATION
HALLMARK INFLAMMATORY RESPONSE
HALLMARK KRAS SIGNALING UP
HALLMARK COMPLEMENT
GeneRatio
p.adjust
Count
F
Macrophage Gene Expression Enrichment Score
G
Neutrophil Gene Expression Enrichment Score
EGFR ON CRP
EGFR OFF CRP SCLC
EGFR OFF CRP LUAD
EGFR ON ERP
EGFR OFF ERP
H
LUAD
CRP
EGFR Inactivation
ERP
EGFR Inactivation
NEUROD1+ SCLC
ASCL1+ SCLC
SCLC
PC1 (43.42%)
PC2 (18.49%)
EGFR ON CRP
EGFR OFF CRP LUAD
EGFR OFF CRP NCAM+ LUAD
EGFR OFF CRP SCLC
EGFR OFF ERP
EGFR ON ERP
EGFRmutant LUAD with RB1/TP53 Inactivation
Recurrence After EGFR Inactivation
SCLC
EED Inactivation
LUAD with Mucinous Features
I
Cell Count (normalized)
DOX+
DOX-
1525
1544
1533
ns

Supplemental Figure 13. EED Inactivation at Tumor Initiation Prevents SCLC Transformation but Induces Mucinous Differentiation and Immune Infiltration in an *EGFR*-Mutant GEMM after EGFR Oncogene Withdrawal. Related to Figure 8.
(**A**) Heatmaps of gene set enrichment scores of the indicated gene lists in *EGFR*-Mutant lung tumor genotypes indicated. Blue is low, yellow is high. IMA=invasive mucinous adenocarcinoma. TAM=tumor associated macrophages. (**B**) Volcano plot of RNA-seq data from Figure 8G of EGFR Dox-OFF CRP tumors with SCLC histological transformation (HT) vs. EGFR Dox-ON CRP tumors. Vertical lines, fold change=2. Horizontal line, adjusted p value=0.05. (**C**) Hallmark pathway enrichment analysis of differentially expressed genes in EGFR Dox-OFF CRP tumors with SCLC transformation vs. EGFR Dox-ON CRP tumors. (**D**) Gene set enrichment score of MAPK target genes (Wagle, et al., 2018(4)) in the *EGFR*-Mutant lung tumor genotypes indicated. Student's t test was used to calculate two-sided p-values. (**E**) Hallmark pathway enrichment analysis of differentially expressed genes in EGFR Dox-OFF CRP tumors that recurred as LUAD vs. EGFR Dox-ON CRP tumors. BH adjusted p-values were visualized by the bar color, red=low, blue=high. Count: Enriched gene counts. (**F** and **G**) Bar plots of gene set enrichment score of tumor associated macrophage gene signature (**F**) and neutrophil gene signature (**G**). (**H**) Left-PCA plot of RNA-seq data from the *EGFR*-Mutant lung tumor genotypes indicated. Right-schematic of histologic recurrence phenotypes after EGFR inactivation showing EED inactivation inhibits SCLC and promotes LUAD with mucinous features. (**I**) Bar plot of normalized cell counts of EGFR Dox-ON CRP cells treated with or without Dox (1 μg/mL) for 6 days. Student's t-test was used to calculate two-sided p-values. *=$p<0.05$, **=$p<0.01$, ***=$p<0.001$, ****=$p<0.0001$. CRP: sgControl; sg*Rb1*; *Trp53*-/-, ERP: sg*Eed*; sg*Rb1*; *Trp53*-/-. EGFR ON=Dox ON, EGFR OFF=Dox withdrawal.

Fig. S14

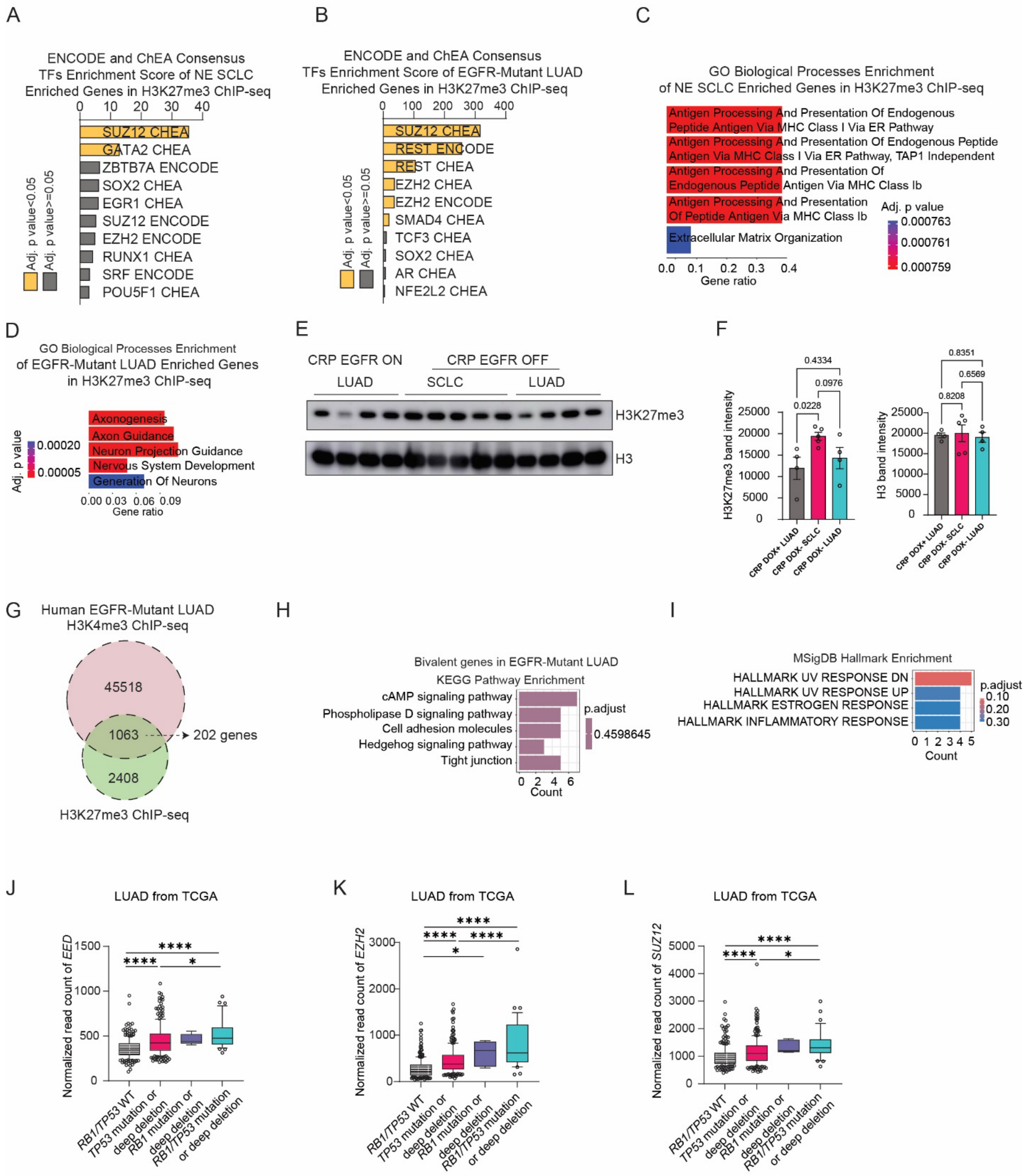
A
ENCODE and ChEA Consensus
TFs Enrichment Score of NE SCLC
Enriched Genes in H3K27me3 ChIP-seq
0 10 20 30 40
SUZ12 CHEA
GATA2 CHEA
ZBTB7A ENCODE
SOX2 CHEA
EGR1 CHEA
SUZ12 ENCODE
EZH2 ENCODE
RUNX1 CHEA
SRF ENCODE
POU5F1 CHEA
Adj. p value<0.05
Adj. p value>=0.05
B
ENCODE and ChEA Consensus
TFs Enrichment Score of EGFR-Mutant LUAD
Enriched Genes in H3K27me3 ChIP-seq
0 100 200 300 400
SUZ12 CHEA
REST ENCODE
REST CHEA
EZH2 CHEA
EZH2 ENCODE
SMAD4 CHEA
TCF3 CHEA
SOX2 CHEA
AR CHEA
NFE2L2 CHEA
Adj. p value<0.05
Adj. p value>=0.05
C
GO Biological Processes Enrichment
of NE SCLC Enriched Genes in H3K27me3 ChIP-seq
Antigen Processing And Presentation Of Endogenous Peptide Antigen Via MHC Class I Via ER Pathway
Antigen Processing And Presentation Of Endogenous Peptide Antigen Via MHC Class I Via ER Pathway, TAP1 Independent
Antigen Processing And Presentation Of Endogenous Peptide Antigen Via MHC Class Ib
Antigen Processing And Presentation Of Peptide Antigen Via MHC Class Ib
Extracellular Matrix Organization
0.0 0.1 0.2 0.3 0.4
Gene ratio
Adj. p value
0.000763
0.000761
0.000759
D
GO Biological Processes Enrichment
of EGFR-Mutant LUAD Enriched Genes
in H3K27me3 ChIP-seq
Axonogenesis
Axon Guidance
Neuron Projection Guidance
Nervous System Development
Generation Of Neurons
Adj. p value
0.00020
0.00005
0.00 0.03 0.06 0.09
Gene ratio
E
CRP EGFR ON
CRP EGFR OFF
LUAD
SCLC
LUAD
H3K27me3
H3
F
H3K27me3 band intensity
0.4334
0.0976
0.0228
H3 band intensity
0.8351
0.6569
0.8208
25000
20000
15000
10000
5000
0
CRP DOX+ LUAD
CRP DOX- SCLC
CRP DOX- LUAD
G
Human EGFR-Mutant LUAD
H3K4me3 ChIP-seq
45518
1063
2408
202 genes
H3K27me3 ChIP-seq
H
Bivalent genes in EGFR-Mutant LUAD
KEGG Pathway Enrichment
cAMP signaling pathway
Phospholipase D signaling pathway
Cell adhesion molecules
Hedgehog signaling pathway
Tight junction
0 2 4 6
Count
p.adjust
0.4598645
I
MSigDB Hallmark Enrichment
HALLMARK UV RESPONSE DN
HALLMARK UV RESPONSE UP
HALLMARK ESTROGEN RESPONSE
HALLMARK INFLAMMATORY RESPONSE
0 1 2 3 4 5
Count
p.adjust
0.10
0.20
0.30
J
LUAD from TCGA
Normalized read count of EED
1500
1000
500
0
RB1/TP53 WT
TP53 mutation or deep deletion
RB1 mutation or deep deletion
RB1/TP53 mutation or deep deletion
K
LUAD from TCGA
Normalized read count of EZH2
3000
2000
1000
0
RB1/TP53 WT
TP53 mutation or deep deletion
RB1 mutation or deep deletion
RB1/TP53 mutation or deep deletion
L
LUAD from TCGA
Normalized read count of SUZ12
5000
4000
3000
2000
1000
0
RB1/TP53 WT
TP53 mutation or deep deletion
RB1 mutation or deep deletion
RB1/TP53 mutation or deep deletion

Supplemental Figure 14. H3K27me3 Represses LUAD Oncogenic Programs in Human SCLC Through Bivalent Silencing of RAS, PI3K, and MAPK Pathway Genes. Related to Figure 9.

(**A** and **B**) Bar plot of ENCODE and ChEA Consensus TFs enrichment score from top enriched transcription factors in human NE SCLC (**A**) and *EGFR*-Mutant LUAD (**B**) PDX tumors whose target genes have significantly gained H3K27me3 peaks. Yellow: significantly enriched with adjusted p-value<0.05, grey: adjusted p value>0.05. (**C** and **D**) Bar plot of gene ontology biological processes enrichment of genes that have significantly gained H3K27me3 peaks in human NE SCLC (**C**) and *EGFR*-Mutant LUAD (**D**). BH adjusted p-values were visualized by the bar color, red=low, blue=high. Gene ratio=enriched gene number/total number of genes that have significantly gained H3K27me3 peaks in human NE SCLC (**C**) and *EGFR*-Mutant LUAD (**D**). (**E** and **F**) Immunoblot analysis using histone lysates of H3K27me3 and Histone 3 (**E**) with band intensity quantifications of H3K27me3 and Histone 3 (**F**) from CRP EGFR ON LUAD, EGFR OFF SCLC, and EGFR OFF LUAD tumors. (**G-I**) Schematic of identifying bivalent genes in human *EGFR*-Mutant LUAD by overlapping H3K27me3 and H3K4me3 ChIP-seq (**G**), Bar plots of enriched KEGG pathways (**H**) and hallmark pathways (**I**) of bivalent genes in human *EGFR*-Mutant LUAD PDX tumors. BH adjusted p-values are visualized by the bar color, red=low, blue=high. Count: Enriched gene counts. (**J-L)** Box plots of RNA-seq batch normalized expression of *EED* (**J**), *EZH2* (**K**), *SUZ12* (**L**) from human LUAD tumors with *RB1* and *TP53* genetic alterations indicated in the Cancer Genome Atlas (TCGA)(5). *RB1/TP53* WT: n=241 tumors, *TP53* mutation or deep deletion: n=231 tumors, *RB1* mutation or deep deletion: n=5 tumors, *RB1/TP53* mutation or deep deletion: n=33 tumors. One-way ANOVA with Tukey test was used to calculate two-sided p values adjusted for multiple comparisons. *=p<0.05, **=p<0.01, ***=p<0.001, ****=p<0.0001.

Fig. S15

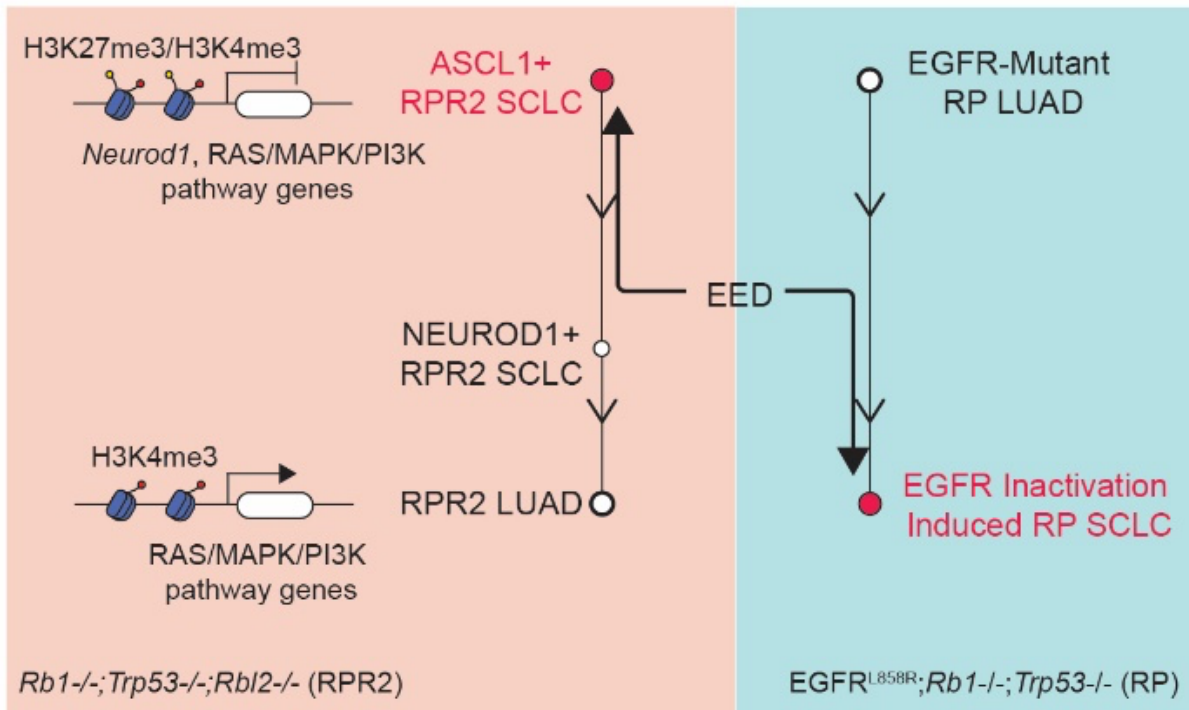

Supplemental Figure 15. Schematic Showing How EED Supports the SCLC Neuroendocrine Phenotype and LUAD-to-SCLC Histological Transformation.
When SCLC tumorigenesis is initiated in the RPR2 GEMM model, inactivation of EED delays tumor formation, and tumors eventually escape EED loss by switching their histology to lung adenocarcinoma (LUAD). This occurs through a transient NEUROD1-positive EMT-like intermediate cell state that requires cues from the tumor microenvironment. When LUAD tumors are initiated by the EGFR L858R oncogene and recur after EGFR oncogene withdrawal, only tumors with EED-intact undergo histological transformation to SCLC as a mechanism of resistance to EGFR withdrawal. Together both models show that intact EED supports the SCLC neuroendocrine phenotype during lung cancer pathogenesis. Mechanistically, EED maintains the SCLC neuroendocrine identity by directly repressing bivalent chromatin-marked RAS, PI3K, and MAPK pathway genes to oppose LUAD oncogenic signaling. RTK: receptor tyrosine kinase. EMT: epithelial to mesenchymal transition. Figure created with BioRender.

Li et al. Supplemental Table Legends

Supplemental Table 1. EED-Inactivated and EED-WT RPR2 GEMM Histology and Pathology, related to Figure 1.
Tab 1. Pathology and metastasis of EED-Inactivated and EED-WT RPR2 GEMM. Tab 2. IHC staining for EED, H3K27me3, GFP, and Synaptophysin (SYP) of EED-Inactivated and EED-WT RPR2 GEMM. Tab 3. Gene list of 120 epigenetic regulators related to Figure 1G.
Supplemental Table 2. Differential Analysis of EED-Inactivated RPR2 LUAD vs. EED-WT SCLC and Gene Lists, related to Figure 2.
Tab 1. Differential expression of RNA-sequencing of EED-Inactivated RPR2 LUAD vs. EED-WT SCLC. Tab 2. Gene lists used in enrichment analysis. Tab 3. Differential binding table of H3K27ac ChIP-seq data. Tab 4. Differential peak table of ATAC-seq data. Tab 5. Signature genes from Quintanal-Villalonga et al., 2021 (2) used for correlation analysis.
Supplemental Table 3. Differential Analysis of EED-Inactivated RPR2 SCLC vs. EED-WT SCLC, 1014 EED-Isogenic RNA-seq Data, and Gene Lists, related to Figure 3.
Tab 1. Differential expression in RNA-sequencing data of EED-Inactivated RPR2 SCLC and EED-WT SCLC. Tab 2. Gene lists used in enrichment analysis. Tab3. FPKM values of NE, non-NE, and gastrointestinal makers from EED-Inactivated RPR2 SCLC, LUAD, and EED-WT RPR2 tumor RNA-seq,Tab 4. Differential binding table of H3K27ac ChIP-seq of EED-Inactivated RPR2 SCLC and EED-WT SCLC. Tab 5. Differential peak table of ATAC-seq of EED-Inactivated RPR2 SCLC and EED-WT SCLC. Tab 6. Differential expression in RNA-sequencing of 1014 EED-WT RPR2 SCLC cells CRISPR inactivated for EED (sgEed) vs. transduced with a non-targeting sgRNA (sgControl).
Supplemental Table 4. Functional Direct EED Targets and Transcriptomic Analysis of EED Re-expression in EED-Inactivated RPR2 LUAD Cell Lines, related to Figure 3.
Tab 1. Peaks with overlap between EED and H3K27me3 ChIP-seq in EED-WT RPR2 SCLC tumors and their closest genes. Tab 2. Functional direct EED targets in EED-Inactivated RPR2 SCLC. Tab 3. Functional direct EED targets in EED-Inactivated RPR2 LUAD lung tumors. Tab 4. Differential expression of RNA-seq data comparing re-expression of EED-WT vs EED inactive mutant as a negative control in the EED-Inactivated RPR2 LUAD cell line 1339. Tab 5. Differential expression of RNA-seq data comparing re-expression of EED-WT vs EED inactive mutant as a negative control in the EED-Inactivated RPR2 LUAD cell line 1344.
Supplemental Table 5. NEUROD1, EED, and p-ERK IHC Quantification in EED-isogenic Tumors, and Table of Enriched Cell-Cell Interactions in EED-Inactivated RPR2 LUADs, related to Figures 4 and 5.
Tab 1. Quantification of NEUROD1 IHC staining. Tab 2. IHC staining for ASCL1, NEUROD1, and POU2F3 on SCLC components in EED-Inactivated RPR2 LUAD. Tab 3. IHC staining for EED and p-ERK on 1014 EED-isogenic tumor xenografts. Tab 4. Gene lists related to Figure 4 and 5. Tab 5. Tumor-tumor microenvironment interaction enriched in EED-Inactivated RPR2 LUAD tumors.
Supplemental Table 6. H3K4me3 and H3K27me3 Bivalency Analysis in Mouse and Human Lung Tumors, related to Figures 7 and 9.
Tab 1. Bivalent peaks (shared peaks between H3K27me3 and H3k4me3) in EED-WT RPR2 SCLC tumors. Tab 2. Gene lists of bivalent or non-bivalent direct EED targets in EED-Inactivated RPR2 SCLC or EED-Inactivated RPR2 LUAD. Tab 3. H3K27me3 ChIP-seq differential binding table with annotations comparing human *EGFR*-Mutant LUAD to human NE SCLC PDX tumors. Tab 4. Gene lists of bivalent genes in human *EGFR*-Mutant LUAD and NE SCLC PDX tumors.
Supplemental Table 7. Histology and Differential Expression Analysis of *EGFR*-Mutant CRP and ERP GEMMs, related to Figure 8.
Tab 1. Pathology, metastasis, and endpoint status of *EGFR*-Mutant CRP and ERP GEMMs. Tab 2. IHC staining of primary tumor and metastases *EGFR*-Mutant CRP and ERP GEMMs. Tab 3. Differential expression table of EGFR Dox-OFF CRP SCLC tumors vs. *EGFR* Dox-ON CRP

tumors. Tab 4. Table of FPKM values of all genes from RNA-seq data from *EGFR*-Mutant CRP and ERP lung tumors. Tab 5. Gene lists related to Figure 8 and Supplemental Figure 12.

Li et al. Supplemental Methods

sgRNA Oligonucleotides for the Adenoviral sgRNA Expression Vector to Create RPR2 Genetically Engineered Mouse Model

The sgRNA oligos used (including BsmBI sites) for the adenoviral vectors are the following: *Rb1* mouse #11 sense (5'- CACCGCAACTAGAAAATGATACG-3'), *Rb1* mouse #11 anti-sense (5'-AAACCGTATCATTTTCTAGTTGC-3'), *Trp53* mouse #8 sense (5'-CACCGGTGTAATAGCTCCTGCATGG-3'), *Trp53* mouse #8 anti-sense (5'-AAACCCATGCAGGAGCTATTACACC-3'), *Rbl2* mouse #6 sense (5'-CACCGAGGAGGATGGCGACGCCG-3'), *Rbl2* mouse #6 anti-sense (5'-AAACCGGCGTCGCCATCCTCCTC-3'), *Eed* mouse #1 sense (5'-CACCGACAAATACGCCAAATGCACC-3'), *Eed* mouse #1 anti-sense (5'-AAACGGTGCATTTGGCGTATTTGTC-3'), *Eed* mouse #2 sense (5'-CACCGTGCACCAGGAAGGAAAAGCT-3'), *Eed* mouse #2 anti-sense (5'-AAACAGCTTTTCCTTCCTGGTGCAC-3'), *C0111 (*Non-targeting sgRNA, sgControl) sense (5'-CACCGGGAGGCTAAGCGTCGCAA-3'), *C0111* (Non-targeting sgRNA, sgControl) anti-sense (5'- AAACTTGCGACGCTTAGCCTCCC-3').

Adenoviral sgRNA Expression Vector Cloning to Create RPR2 Genetically Engineered Mouse Model

Effective sgRNAs targeting mouse *Rb1*, *Trp53*, and *Rbl2* were first validated using lentiviral vectors as described previously (6). Effective sgRNAs targeting mouse *Eed* were validated in mouse embryonic fibroblasts expressing Cas9. The cloning method for generation of adenoviral sgRNA expression vectors encoding CMV-Cre recombinase and sgRNAs targeting *Rb1, Trp53, and Rbl2* and "T" sgRNA (sg*Eed*#1, sg*Eed*#2, or a non-targeting sgRNA control, *C0111*) was described previously(7, 8). Briefly, a pENTR223-CMV-Cre-U6-sgX-U6-sgRb1-U6-sgTrp53-U6-sgRbl2 where X is sg*Eed*#1, sg*Eed*#2, or sgC0111, was used in an LR recombination reaction to clone the 4 pENTR223-CMV-Cre-U6-sgX-U6-sgRb1-U6-sgTrp53-U6-sgRbl2 vectors described

above into pAd-PL DEST (Invitrogen) according to the manufacturer's instructions. The recombinants were transformed into HB101 cells and ampicillin-resistant colonies were screened by restriction digestion of miniprep DNA and subsequently validated by sequencing. The sgRNA oligos used (including BsmBI sites) are included in the supplemental data.

Adenoviral sgRNA Expression Vector Cloning to Create *EGFR*-Mutant Genetically Engineered Mouse Model with Concurrent *Rb1* and *Trp53* Mutation

The cloning method for generation of adenoviral sgRNA expression vectors encoding CMV-Cre recombinase and sgRNAs targeting *Rb1* and "T" sgRNA (sgEed#1, sgEed#2, or a non-targeting sgRNA control) is as follows: The DNA fragment containing CMV-Cre-U6-sgX-U6-sgRb1 was generated by inverse polymerase chain reaction (PCR) using KOD Xtreme™ Hot Start DNA Polymerase (Sigma-Aldrich, Cat# 71975) with primers (sense: 5'-CACCCAGCTTTCTTGTACAAAGTTGGC-3', and anti-sense: 5'-TTGTACAAGAAAGCTGGGTGTTGTATA-3') from the pENTR223-CMV-Cre-U6-sgX-U6-sgRb1-U6-sgTrp53-U6-sgRbl2 template. Linear DNA fragment was annealed into a circular plasmid by Gibson Assembly (NEB, cat# E5510S) according to the manufacturer's instructions. The plasmid was then transformed in HB101 cells, and isolated using the QIAprep Spin Miniprep Kit – Plasmid Purification Kit (Qiagen, 27106). Whole plasmid sequencing was performed to confirm the correct sequences (pENTR223-CMV-Cre-U6-sgX-U6-sgRb1 where sgX is sgEed#1, sgEed#2, or a non-targeting sgRNA control). Subsequently LR reactions were performed with pAd-PL DEST as described previously(7, 8) to make pAd-PL CMV-Cre-U6-sgX-U6-sgRb1 where sgX is sgEed#1, sgEed#2, or a non-targeting sgRNA control.

Adenovirus Production and Purification

Adenoviral production and purification were performed as described previously(7). 5 μg of the adenovirus vector (pAd/PL Invitrogen #V494-20) containing the desired sgRNA sequences and Cre recombinase expression cassette (see above) was digested with PacI (New England Biolabs)

for 2 hours at 37°C according to the manufacturer's instructions and column purified using Qiagen's gel extraction kit. 1 μg of PacI-digested pAd/PL was transfected into 1.5 X 10^6 293AD cells plated on a 6 cm tissue-culture dish using Lipofectamine 2000. The following day, the media was exchanged, and subsequently exchanged every 48 hours thereafter. Once 293AD cells showed evidence of adenovirus production (determined by comet formation with lysis), the cells and supernatant were harvested, which were then subjected to 4 freeze-thaw cycles by alternating between an ethanol dry ice bath and 37°C. Cell debris was removed by centrifugation and the supernatant was collected, passed through a 0.45 μm filter, aliquoted, and frozen at -80°C until use.

To generate high titer adenovirus for *in vivo* experiments, adenovirus was generated as described above. 50 μl of the adenovirus stock was added to each 10 cm tissue-culture dish of 293FT cells plated at 3 X 10^6 cells per dish (4 10 cm dishes in total for each purification). When 293FT cells showed evidence of adenovirus production, as determined by cell rounding and partial detachment (~48-72 hours after addition of adenoviral stock), the cells were collected, and adenovirus was purified using Virabind Adenovirus Purification Kit (Cell Biolabs #VPK-5112). The purified adenovirus was titered using QuickTiter Adenovirus Quantitation Kit (Cell Biolabs #VPK-106) according to the manufacturer's instructions.

Intratracheal Injections

Intratracheal injections were performed as described previously(9). Briefly, mice were anesthetized with ketamine and xylazine and pedal reflexes were monitored to ensure adequate anesthesia. Mice were maintained on a heated stage at 37° C while anesthetized. Mice were hung on stage with their top incisors and intubated with a 22-gauge 1 inch catheter (ThermoFisher Scientific #1484120). Once intubated, adenovirus (4 X 10^8 VP/mouse) in a total volume of 75 μl (diluted in PBS) was added to the catheter and subsequently inhaled by the mice.

Establishing Genetically-Engineered Mouse Models using CRISPR/Cas9

For the RPR2 GEMM, pure congenic *Lox-stop-lox (LSL) Cas9* BL6J mice were purchased from Jackson Labs (Jackson No. 026175) and maintained as homozygous BL6J mice. Genotyping of Cas9 and GFP at the ROSA26 were confirmed for all mice on the study (Transnetyx). Above-mentioned RPR2 adenovirus were intratracheally injected into mice to generate EED-isogenic RPR2 tumors.

For the *EGFR*-Mutant GEMM with *Rb1* and *Trp53* inactivation, *TetO-EGFR*^{L858R}; *Trp53*$^{flox/flox}$; *Rosa26*$^{CAGs\text{-}LSL\text{-}rtTA3\text{-}IRES\text{-}mKate}$ and *TetO-EGFR*^{L858R}; *Trp53*$^{flox/flox}$; *Rosa26*$^{CAGs\text{-}LSL\text{-}Cas9\text{-}GFP}$ were a kind gift from the laboratory of Dr. Katerina Politi (Yale University, New Haven, CT) (10). These mice are mixed BL6/129/FVB background and maintained as homozygous strains. These two strains were bred together to generate *TetO-EGFR*^{L858R}; *Trp53*$^{flox/flox}$; *Rosa26*$^{CAGs\text{-}LSL\text{-}rtTA3\text{-}IRES\text{-}mKate}$; *Rosa26*$^{CAGs\text{-}LSL\text{-}Cas9\text{-}GFP}$ mice (referred to hereafter as *EGFR*-Mutant GEMM experimental mice). Genotyping of *EGFR*^{L858R}, *Trp53* $^{flox/flox}$, and *Cas9, GFP*, and *rtTA3* at the *ROSA26* locus were confirmed for all mice (Transnetyx). For experiments, pAd-PL CMV-Cre-U6-sgEed#2-U6-sgRb1 or pAd-PL CMV-Cre-U6-sgControl-U6-sgRb1 adenoviruses were intratracheally injected as above into the *EGFR*-Mutant GEMM experimental mice and then all mice were fed doxycycline-containing chow (625 ppm, cat# 5AW9, ScottPharma Solutions Inc.). For the control de novo LUAD tumors in Figure 4A, RPR2 adenovirus were intratracheally injected into the *EGFR*-Mutant GEMM experimental mice followed by administration of doxycycline-containing chow (625 ppm) after 1 week.

Housing conditions for mice at the DFCI Vivarium include a 12 hour/12 hour day-night cycle where temperature is maintained at 72 F. Roughly equal numbers of male and female 3-4 month-old mice were intratracheally injected with 4 X 10^8 VP/mouse adenovirus. All mice were monitored at least twice a week and euthanized when they became symptomatic (primarily respiratory distress), moribund, or lost 15% of their total body weight. The maximal tumor size allowed by the DFCI Animal Care and Use Committee is 2 cm and the maximal tumor size was not exceeded in

any of our studies. Upon euthanization, half of the lung tumor specimen was immediately flash frozen on dry ice for subsequent DNA, RNA, and protein analysis, while the other half and the rest of lung was fixed in 10% formalin for 24 hours and then stored in 70% ethanol prior to paraffin embedding. For some tumors, cell lines were generated (see method). Livers, kidneys, and brains were harvested and fixed and embedded as above. Slides were made for H&E and IHC. H&E slides were analyzed by a specialized rodent pathologist Dr. Roderick Bronson, or pulmonary pathologist Dr. Marina Vivero for diagnosis.

Generation of Cell Lines from Mouse Tumors and Cell Culture

Cell line generation and culture were performed as described previously(6). sgControl (EED-WT) RPR2 (631, 1014) cell lines were generated from CRISPR-based SCLC GEMMs (see above) as described previously(7). Once tumors developed, mice were euthanized with CO2 and their tumors were quickly extracted, washed in ice cold PBS, and minced several times using an ethanol sterilized razor blade. 3mL of collagenase/hyaluronidase (Stem cell biology #07912) diluted 1:10 in complete RPMI media containing [10% FBS, P/S, and HITES (10 nM hydrocortisone (Sigma Aldrich # H0135), Insulin-Transferrin-Selenium (Gemini #400-145), and 10 nM beta-estradiol (Sigma Aldrich# E2257), 100 U/mL of penicillin (P), and 100 µg/mL of streptomycin (S)], and 1mL dispase (Corning # 354235) was added to the tumor, and incubated at 37°C for 20-40 minutes with periodic pipetting ~10 every minutes (until most of the tumor cells were in suspension). The cells were then collected, centrifuged at 200 x g for 5 minutes, resuspended in complete RPMI media (see above), filtered through a 70 µm cell strainer (BD #352350), centrifuged again at 1000 rpm for 5 minutes, resuspended in fresh RPMI HITES media and placed in ultra-low adherence tissue culture dishes (Corning #3471). Media was subsequently replaced every 3 days. Histopathology on the tumors confirmed SCLC for all cell lines generated. All SCLC cell lines were grown in Ultra-Low Attachment flasks (Corning™ 3814CONV) or plates

(Corning™ 3471) at 37°C in the presence of 5% CO_2. Once established, all cell lines were validated using immunoblot analysis for Cas9 and the SCLC neuroendocrine markers ASCL1.

sg*Eed* RPR2 (1339, 1343, 1344, 1345, 1350) cell lines were generated from EED-Inactivated RPR2 LUAD tumors. The EGFR Dox-ON CRP cell lines (1525, 1533, 1544) were generated from EGFR Dox-ON CRP mice at their endpoint on Dox and then continuously maintained on Dox (1 μg/mL) unless Dox was withdrawn as indicated. Both sg*Eed* RPR2 and EGFR Dox-ON CRP cell lines were made using Mouse Tumor Dissociation Kit (Miltenyi, cat# 130-096-730) following manufacturer's instructions and then grown in RPMI media containing [10% FBS, 100 U/mL of penicillin (P), and 100 μg/mL of streptomycin (S) (P/S)] on tissue culture dishes (Falcon™, cat# 353003) at 37°C in the presence of 5% CO_2. Once established, all cell lines were validated by immunoblot analysis for expression of Cas9, loss of TP53 and RB1 expression, and by FACS for GFP expression. Early passage cell lines were tested for Mycoplasma (Lonza #LT07-218) and were negative, and then were frozen using Bambanker's freezing media (Bulldog Bio). Mouse embryonic fibroblasts expressing Cas9 used to validate the adenoviruses were described previously(6) and cultured in DMEM media with 10% FBS and P/S.

Generation of SCLC Xenograft Model in BL6J and NCr Nude Mice

The syngeneic RPR2 1014 BL6J model was described previously(8). 1014 cells were first transduced with plentiguide-puro lentiviruses containing an sgRNA targeting EED (*Eed* mouse #2 sense (5'-CACCGTGCACCAGGAAGGAAAAGCT-3'), *Eed* mouse #2 anti-sense (5'-AAACAGCTTTTCCTTCCTGGTGCAC-3') (EED-Inactivated) or a non-targeting sgRNA as a control (EED-WT). Immunoblot analysis validated loss of EED protein with increased NEUROD1 expression (see Figure 3I). Then $8x10^6$ RPR2 1014 with EED-WT or EED-Inactivated cells were washed X3 in DPBS and then resuspended in DPBS with 33% Matrigel (Corning cat# 354234) and implanted subcutaneously into 7-9 week-old female albino BL6J mice (C57BL/6J-Tyrc-2J,

stock no. 000058; RRID:IMSR_JAX:000058, Jackson Laboratory) or immunodeficient female NCr nude mice (Taconic #NCRNU). Once established, mice were euthanized with CO2, and half of each tumor was immediately flash frozen on dry ice for subsequent immunoblot analysis.

Human Cell Lines

NCI-H1876, NCI-H2081 and 293FT cells were originally obtained from American Type Culture Collection (ATCC). 293AD cells (AD-100) were obtained from Cell Biolabs. NCI-H1876 and NCI-H2081 cells were maintained in DMEM/F12 media 5% FBS, P/S, and HITES. 293T and 293AD were maintained in DMEM media with 10% FBS and P/S. Early passage cell lines were tested negative for Mycoplasma (Lonza #LT07-218), and then were frozen using Bambanker's freezing media (Bulldog Bio). All experiments were performed with cell lines that were maintained in culture for <3 months at which time an early passage cell lines were thawed. No commonly misidentified cell lines were used in this study.

Pharmacological Inhibitors

The following chemicals (stored at -20˚C or -80˚C) were added to cell culture where indicated: MAK683 (Selleckchem #S8983, stock 10 mM in DMSO), Tazemetostat (Selleckchem #S7128, stock 10 mM in DMSO), Tulmimetostat (Selleckchem #E1497, stock 10 mM in DMSO), Valemetostat (Selleckchem #S8926, stock 10 mM in DMSO), Trametinib (Selleckchem #S2673, stock in 10mM in DMSO) or RMC-6236 (Selleckchem #E1597, stock 10 mM in DMSO).

sgRNA Cloning to Make Lentiviruses

sgRNA cloning to make lentivirus was performed as described previously(11). sgRNA sequences were designed using the Broad Institute sgRNA designer tool (http://portals.broadinstitute.org/gpp/public/analysis-tools/sgrna-design) and synthesized by IDT technologies. The sense and antisense oligonucleotides were mixed at equimolar ratios (0.25

nanomoles of each sense and antisense oligonucleotide) and annealed by heating to 100°C in annealing buffer (1X annealing buffer 100 mM NaCl, 10 mM Tris-HCl, pH 7.4) followed by slow cooling to 30°C for 3 hours. The annealed oligonucleotides were then diluted at 1:400 in 0.5X annealing buffer.

For CRISPR/Cas9 knockout experiments in cells, the annealed oligos were ligated into LentiGuide Puro (Addgene #52963) for experiments in mouse SCLC cell lines. Ligations were done with T4 DNA ligase for 2 hours at 25°C. The ligation mixture was transformed into HB101 competent cells. Ampicillin-resistant colonies were screened by restriction digestion of miniprep DNAs and subsequently validated by DNA sequencing.

The following sgRNA oligos were used for LentiGuide Puro vector for CRISPR knockout experiments: *Eed* mouse #1 sense (5'- CACCGACAAATACGCCAAATGCACC-3'), *Eed* mouse #1 anti-sense (5'- AAACGGTGCATTTGGCGTATTTGTC-3'), *Eed* mouse #2 sense (5'- CACCGTGCACCAGGAAGGAAAAGCT-3'), *Eed* mouse #2 anti-sense (5'- AAACAGCTTTTCCTTCCTGGTGCAC-3'), *C0111* (Non-targeting sgRNA, sgControl) sense (5'- CACCGGGAGGCTAAGCGTCGCAA-3'), *C0111* (Non-targeting sgRNA, sgControl) anti-sense (5'- AAACTTGCGACGCTTAGCCTCCC-3').

Lentivirus Production

Lentivirus production was performed as described previously(11). Lentiviruses were made by Lipofectamine 2000-based co-transfection of 293FT cells with the respective lentiviral expression vectors and the packaging plasmids psPAX2 (Addgene #12260) and pMD2.G (Addgene #12259) at a ratio of 4:3:1. Virus-containing supernatant was collected at 48 and 72h after transfection, pooled together (15 mL total per 10-cm tissue culture dish), passed through a 0.45-μm filter, aliquoted, and frozen at -80°C until use.

Lentiviral Infection

Lentivirus infection was performed as described previously(11). The cells were counted using a Vi-Cell XR Cell Counter (Beckman Coulter) and 2 $X10^6$ cells were resuspended in 1mL lentivirus with 8 µg/mL polybrene in individual wells of a 12 well plate. The plates were then centrifuged at 448 x g for 2h at 30° C. 16 hours later the virus was removed, and cells were grown for 72 hours before being placed under drug selection. Cells were selected in puromycin (0.5 µg/mL).

Immunoblotting

Immunoblotting was performed as described previously(11). Cell pellets were lysed in a modified EBC lysis buffer (50mM Tris-Cl pH 8.0, 250 mM NaCl, 0.5% NP-40, 5 mM EDTA) supplemented with a protease inhibitor cocktail (Complete, Roche Applied Science, #11836153001) and phosphatase inhibitors (PhosSTOP Sigma #04906837001). Soluble cell extracts were quantified using the Bradford Protein Assay. 20 µg of protein per sample was boiled after adding 3X sample buffer (6.7% SDS, 33% Glycerol, 300 mM DTT, and Bromophenol Blue) to a final concentration of 1X, resolved by SDS-PAGE using either 10% or 8% SDS-PAGE, semi-dry transferred onto nitrocellulose membranes, blocked in 5% milk in Tris-Buffered Saline with 0.1%Tween 20 (TBS-T) for 1h, and probed with the indicated primary antibodies overnight at 4°C. Membranes were then washed three times in TBS-T, probed with the indicated horseradish peroxidase conjugated (HRP) secondary antibodies for 1h at room temperature, and washed three times in TBS-T. Bound antibodies were detected with enhanced chemiluminescence (ECL) western blotting detection reagents (Immobilon, Thermo Fisher Scientific, #WBKLS0500) or Supersignal West Pico (Thermo Fisher Scientific, #PI34078). The primary antibodies and dilutions used were: Rabbit anti-EED (Cell Signaling #85322S, 1:1000), rabbit anti-ASCL1 (Abcam #Ab211327, 1:1000), rabbit anti-NEUROD1 [EPR4008] (Abcam #Ab109224, 1:1000), rabbit anti-RAS (E8N8L) (Cell Signaling #67648, 1:1000), rabbit anti-Rb1 (Abcam #181616, 1:2000), rabbit rodent specific anti-p53 (D2H9O) (Cell Signaling Technology #32532, 1:1000), rabbit anti-p130 (Abcam #Ab76234, 1:1000), rabbit anti-Cre Recombinase (D7L7L) (Cell Signaling Technology

#15036, 1:1000), Mouse Anti-Cas9 (Cell Signaling Technology #14697, 1:1000), rabbit anti-HNF4A (Cell Signaling Technology #3113, 1:1000), rabbit anti-p-ERK (Cell Signaling Technology #4370, 1:1000), rabbit anti-total ERK (Cell Signaling Technology #9102, 1:1000), rabbit anti-p-AKT S473 (Cell Signaling Technology #4060, 1:1000), rabbit anti-p-AKT T308 (Cell Signaling Technology #9275, 1:1000), rabbit anti-total AKT (Cell Signaling Technology #9272, 1:1000), rabbit anti-p-S6 (Cell Signaling Technology #4858, 1:1000), rabbit anti-total-S6 (Cell Signaling Technology #2217, 1:1000), mouse anti-β-actin (clone AC-15) (Sigma, #A3854, 1:25,000), mouse α-Vinculin (hVIN-1) (Sigma, # V9131, 1:10000). Histone extractions were performed as described previously(6) with the following primary antibodies: Rabbit anti-Histone H3 (D1H2) (Cell Signaling Technology #4499S, 1:1000), rabbit anti-Di-Methyl-Histone H3 (Lys36) (C75H12) (Cell Signaling Technology #2901, 1:1000), and rabbit anti-Tri-Methyl-Histone H3 (Lys27) (Cell Signaling Technology #9733S, 1:1000). The secondary antibodies and dilutions used were: Goat Anti-Mouse (Jackson ImmunoResearch #115-035-003) and Goat anti-Rabbit (Jackson ImmunoResearch #111-035-003) and used at 1:5000. Immunoblots were quantified using ImageJ (1.54g).

Bulk RNA-Sequencing and Analysis

Tumors were harvested at necropsy and were flash-frozen. RNA was extracted using Quick-RNA™ Miniprep kit (Zymo Research, Cat # R1055) including a DNase digestion step according to the manufacturer's instructions and RNA sequencing was performed as described below. For all RNA-seq except for data in Figure 8J, total mRNA samples in each experiment were submitted to Novogene Inc. The libraries for RNA-seq are prepared using NEBNext Ultra II non-stranded kit. Paired end 150bp sequencing was performed on Novaseq6000 sequencer using S4 flow cell. For RNA-seq in Figure 8J, total mRNA samples were submitted to Plasmidsaurus. Sequencing was performed using 3' end counting on an Illumina NovaSeq X Plus platform. Sequencing reads were mapped to the mm10 genome by STAR. Statistics for differentially expressed genes were

calculated by DESeq2 (1.36.0). Bulk RNA-sequencing heatmaps were generated by calculating z score from log transformed FPKM values. To calculate z score, a data point was subtracted the mean and then divided the result by the standard deviation.

Gene Set Enrichment Analysis

GSEA software was downloaded from the Gene Set Enrichment Analysis website [http://www.broad.mit.edu/gsea/downloads.jsp](12). Pre-ranked GSEA was performed using hallmark gene sets from the human MSigDB Collections(13) in Figures 2D and 3C, top 100 neuroendocrine genes, ASCL1 target genes(14) (Supplemental Table 2, Tab 2) in Figure 2D, human and mouse mucinous lung tumor signature(3) (Supplemental Table 2, Tab 2) in Supplemental Figure 2H, and ASCL1 correlated genes(14), NEUROD1 correlated genes(14), IMPOWER133-SCLC-N (NMF1) and SCLC-A (NMF2) gene signatures(15) (Supplemental Table 3, Tab 2) in Figure 3E.

The Gene Set Variation Analysis (GSVA) method from the GSVA Bioconductor package (version 1.50.5), was used to calculate enrichment scores from tuft cell marker gene lists (Supplemental Table 5, Tab 4) (Supplemental Figure 4B) and top 100 NE gene list (Supplemental Table 2, Tab 2) (Supplemental Figure 4C) using normalized read counts from bulk EED-isogenic RPR2 tumor RNA-seq data and from gene signatures of MAPK targets(4), neuroendocrine markers(15), tumor proliferation rate(15), hallmark_MYC, hallmark_G2M, and neutrophil markers(15) (Supplemental Table 7, Tab 5) and human and mouse mucinous lung tumor signature (Supplemental Table 2, Tab 2) (Supplemental Figure 13, A and D) using normalized read counts from bulk RNA-seq data from *EGFR*-Mutant CRP and ERP GEMM lung tumors.

For gene set over-representation analysis, candidate gene sets were tested if over-represented against the human hallmark pathways from the MSigDB in Supplemental Figures 3L, 11E, 13, C and E, and 14I, mouse KEGG pathway collection(16) in Figures 6, C, F, 7G, 9, D and G, and Supplemental Figures 10, D and G, and 14H using R package Clusterprofiler (4.8.3). Mouse gene

atlas(17) (Supplemental Figure 2G), Gene Ontology Biological Processes(18) (Supplemental Figure 14, C and D), and ENCODE and ChEA consensus TFs(19) (Figures 2B, and Supplemental Figures 10, C and F, and 14, A and B) using R package enrichR (3.4).

Reverse-Transcriptase Quantitative PCR (RT-qPCR)

RT-qPCR was performed as described previously(20). RNA was extracted using Quick-RNA™ Miniprep kit (Cat#R1055, Zymo Research, CA, USA) according to the manufacturer's instructions. RNA concentration was determined using the Nanodrop 8000 (Thermofisher Scientific). A cDNA library was synthesized using iScript Reverse Transcription Supermix for RT-qPCR (Biorad #1708841) according to the manufacturer's instructions. qPCR were performed using the SsoAdvanced Universal SYBR Green Supermix (Biorad #1725271) according to the manufacturer's instructions. The $\Delta\Delta C_T$ Method was used to analyze data. The C_T values for each primer set were then normalized to the C_T value of *36b4*. The data from Figure 4C was then normalized to the control to determine the relative fold change in mRNA expression. The following probes were used for qPCR with SYBR Green: Mouse *Neurod1* Forward (5'-AGGCTCCAGGGTTATGAGATCG-3')*,* Mouse *Neurod1* Reverse (5'-TGAGAACTGAGACACTCATCTG-3'), Mouse *Met* Forward (5'-GACCTTAAGCGAGAGCACGA-3'), Mouse *Met* Reverse (5'-ATGCACTGTATTGCGTCGTC-3'), Mouse *Hnf4a* Forward (5'-AGAAGATTGCCAACATCAC-3'), Mouse *Hnf4a* Reverse (5'-GGTCATCCAGAAGGAGTT-3'), Mouse *36b4* Forward (5'- CTGTTGGCCAATAAGGTGCC-3'), Mouse *36b4* Reverse (5'-GTTCTGAGCTGGCACAGTGA-3').

Transcriptomic Profile Correlation Analysis

Transcriptomic profiles of never-transformed and pre-transform LUAD and transformed and *de novo* SCLC [Transcript Per Million (TPM) values from RNA-seq data] were obtained from published work by Quintanal-Villalonga et al. 2021(2) and top 100 signature genes from each

group vs. all others (Supplemental Table 2, Tab 5) were determined using log-transformed TPM values with the eBays function in the limma package (3.56.2) in R (4.3.1). Log transformed TPM of signature genes were merged with EED-isogenic RPR2 bulk tumor RNA-seq data, Pearson correlations were calculated with the cor function in base stats package in R (4.3.1). Correlation coefficients were further normalized by calculating z score as described above.

Transcriptomic Data Analysis of the Cancer Genome Atlas (TCGA)

Batch normalized mRNA expression, mutation, and copy number alteration data from Lung adenocarcinoma (LUAD) patient tumors in the TCGA dataset were downloaded from cBioPortal (cbioportal.org)(5). Samples with either mutation or deep deletion (copy number alteration value=-2) in *RB1* and/or *TP53* were categorized as a group and data was analyzed for all LUADs or only LUADs harboring EGFR mutations. Statistical testing between groups were performed using Tukey test to adjust for multiple comparisons.

Immunohistochemistry

Immunohistochemistry was performed on the Leica Bond III automated staining platform using the Leica Biosystems Refine Detection Kit (Leica, DS9800). FFPE tissue sections were baked for 30 minutes at 60°C and deparaffinized (Leica AR9222) prior to staining. Primary antibodies were incubated for 30 minutes, visualized via DAB, and counterstained with hematoxylin (Leica, DS9800). The slides were rehydrated in graded alcohol and coverslipped using the HistoreCore Spectra CV mounting medium (Leica, 3801733). 4 µm-thick sections were cut from formalin-fixed paraffin-embedded mouse tumor samples to perform single IHC studies using antibodies recognizing the following antigens: ASCL1 (rabbit monoclonal antibody clone EPR19840, Abcam; 1:100 concentration, antigen retrieval with citrate for 30 min.); NEUROD1 (rabbit monoclonal antibody clone EPR20766, Abcam; 1:100 concentration, antigen retrieval with citrate for 30 min.); POU2F3 (rabbit polyclonal antibody, Atlas Antibodies, #HPA019652, 1:400 concentration,

antigen retrieval with citrate for 30 min.); EED (rabbit monoclonal antibody clone E4L6E, Cell Signaling Technology; 1:200 concentration, antigen retrieval with EDTA for 30 min.); EGFR mutant-specific L858R antibody (rabbit monoclonal antibody clone 43B2, Cell Signaling Technology; 1:100 concentration, antigen retrieval with citrate for 30 min.); TTF1 (rabbit monoclonal antibody clone EP1584Y, Abcam; 1:200 concentration, antigen retrieval with EDTA for 20 min.); tri-methyl-histone H3 (Lys27) (rabbit monoclonal antibody clone C36B11, Cell Signaling Technology; 1:100 concentration, antigen retrieval with citrate for 30 min.); Synaptophysin (rabbit monoclonal antibody clone SP11, Invitrogen; 1:50 concentration, antigen retrieval with citrate for 30 min.); Phospho-Akt (Thr308) (rabbit polyclonal antibody catalog number 07-1398, MilliporeSigma, 1:400 concentration) with a 20M EDTA antigen retrieval (Leica ER2 AR9640); and Phospho-p44/42 MAPK (Erk1/2) (Thr202/Tyr204) (D13.14.4E, catalog number 4370, Cell Signaling Technology, 1:150 concentration) with a 30M citrate antigen retrieval (Leica, ER1 AR9961).

The immunostained slides were scanned at 40x magnification using the PhenoImager HT slide imager (Akoya Biosciences). For all markers, visual assessment of positive and negative cells was done by two independent pathologists (YNL, GRO). To quantify NEUROD1-positive tumor cells, tumor areas on each slide were identified and manually annotated by two pathologists (YNL, GRO) using the HALO image analysis platform (Indica Labs). The HALO multiplex-IHC v3.2.5 algorithm (Indica Labs) was then used to quantify the number of positive and negative tumor cells, and the percentage of NEUROD1-positive tumor cells was subsequently calculated.

ATAC-sequencing

Nuclei isolation for ATAC-sequencing from mouse tumor tissue was performed using ATAC-Seq Kit (ActiveMotif, cat# 53150) following manufacturer's instruction. Briefly, 30mg frozen mouse RPR2 lung tumor tissues were minced with a razor blade and homogenized in ATAC-seq Lysis

Buffer in a dounce homogenizer. Lysates were then filtered through a 40 micron cell strainer and Trypan blue at 0.4% was used to count 100,000 nuclei which were subsequently rinsed with ice-cold PBS and resuspended in ice-cold ATAC-seq Lysis Buffer. After mixing with Tagmentation Master Mix, tagmentation reactions were incubated at 37 ºC and shaken at 800 RPM for 30 minutes on a thermomixer. Transposed DNA was purified using columns and libraries were amplified following manufacturer's manual. 50 million paired-end reads, where each read is 150 base pairs long, were sequenced on a NextSeq instrument (Illumina).

ATAC-seq Data Analysis

All samples were processed through the computational pipeline developed at the Dana-Farber Cancer Institute Center for Functional Cancer Epigenetics (CFCE) using primarily open-source program CHIPS(21, 22) . Sequence tags were aligned with Burrows-Wheeler Aligner (BWA)(23) to build mm10 and uniquely mapped, non-redundant reads were retained. These reads were used to generate binding sites with Model-Based Analysis of ChIP-Seq 2 (MACS v2.1.1.20160309), with narrow peak option and a q-value (FDR) threshold of 0.01(24). Average peak tracks were calculated with bigwigAverage function in deeptools (3.5.1) and visualized by IGV (v2.14.1)(25). Peaks from all samples were processed using the DiffBind pipeline (v3.18)(26) for differential binding analysis. Briefly, peaks were merged to create a union set of sites. Sequencing depth normalization was performed on each sample and DEseq2 was used to determine differential peaks. Log-transformed fold changes were subsequently shrunk with lfcshrunk for more accurate foldchange estimation. Differential binding peaks from each group were used for motif analysis by the motif search findMotifsGenome.pl in HOMER (v3.0.0)(27), with cutoff q-value≤ 1e-10. The signals of each sample on differential binding sites were visualized by deeptools(28). For principal component analysis, all peaks were used in the analysis and graphic visualization.

Chromatin Immuno-Precipitation Sequencing (ChIP-seq) Analysis of *De Novo* SCLC and *EGFR*-Mutant LUAD Patient Derived Xenograft (PDX) Tumors

ChIP-seq for H3K27me3 and H3K4me3 from 5 NE *de novo* SCLC and 4 *EGFR*-Mutant LUAD PDX samples were previously published(29) and the fastq files were obtained from the Gene Expression Omnibus (GEO accession#: GSE269746). High quality reads passing quality control using fastp were aligned to HumanG1Kv37 reference with BWA(30). Uniquely mapped, non-redundant reads were generated by picard tools (2.19.0) and samtools (1.9) and subsequently used to generate binding sites with MACS2 (MACS v2.1.1.20160309) with q-value (FDR) threshold of 0.01 and narrow peak option for H3K4me3 and broad peak option for H3K27me3(24). Peaks from all samples were processed using the DiffBind pipeline (v3.18)(26) for differential binding analysis as described above.

Mouse EED-Isogenic RPR2 GEMM Tumors ChIP-seq Sample Preparation and Sequencing

Frozen mouse tumor tissue was dry pulverized in liquid nitrogen until it was finely powdered. Pulverized tissue was resuspended for crosslinking in 2 mM of disuccinimidyl glutarate (DSG, Pierce) for 45 minutes at room temperature. The crosslinked material was pelleted by centrifugation at 2500 RCF for 5 min and resuspended in 1 ml of 1% formaldehyde for 10 minutes. Formaldehyde was quenched with 0.125 M glycine for 5 minutes at room temperature and then the crosslinked material washed with PBS. Washed cells were now pelleted and resuspended in 500 µl of 1% SDS (50 mM Tris-HCl pH 8, 10 mM EDTA) and sonicated for 10 minutes using Covaris E220 sonicator at the following settings: 140 peak incident power, 5% duty factor and 200 cycles per burst) in 1 ml AFA fiber millitubes. Chromatin was immunoprecipitated with 5 µg of antibody (EED: Cell Signaling, E4L6E; H3K27ac: Diagenode, C15410196; H3K27me3: Active Motif, 61018; H3K4me3: Abcam, ab8580). 5 µg of chromatin was used for histone mark ChIPs, and 40 µg of chromatin was used for EED ChIPs. Imunoprecipitated Chromatin was pulled down using Protein A/G beads (Invitrogen Dynabeads, 1000-2D and 1000-4D). Chromatin antibody

complex immobilized on beads was washed 6X with RIPA buffer and eluted in 1% SDS and 0.1M Sodium bicarbonate. ChIP-ed DNA was crosslinked with Proteinase K and column purified. ChIP-seq libraries were made using Swift DNA Library Prep Kit (Swift Biosciences, 10024). 75bp paired end reads were sequenced on a NextSeq instrument (Illumina).

ChIP-seq Analysis and Peak Calling of Mouse EED-Isogenic RPR2 GEMM Tumors

Peak calling and data analysis: All samples were processed through the computational pipeline developed at the Dana-Farber Cancer Institute Center for Functional Cancer Epigenetics (CFCE) using primarily open-source programs(21, 22). Sequence tags were aligned with Burrows-Wheeler Aligner (BWA)(30) to build mm9 and uniquely mapped, non-redundant reads were retained. These reads were used to generate binding sites with Model-Based Analysis of ChIP-Seq 2 (MACS v2.1.1.20160309), with a q-value (FDR) threshold of 0.01(24). We evaluated multiple quality control criteria based on alignment information and peak quality: (i) sequence quality score; (ii) uniquely mappable reads (reads that can only map to one location in the genome); (iii) uniquely mappable locations (locations that can only be mapped by at least one read); (iv) peak overlap with Velcro regions, a comprehensive set of locations – also called consensus signal artifact regions – in the genome that have anomalous, unstructured high signal or read counts in next-generation sequencing experiments independent of cell line and of type of experiment; (v) number of total peaks (the minimum required was 1,000); (vi) high-confidence peaks (the number of peaks that are tenfold enriched over background); (vii) percentage overlap with known DHS sites derived from the ENCODE Project (the minimum required to meet the threshold was 80%); and (viii) peak conservation (a measure of sequence similarity across species based on the hypothesis that conserved sequences are more likely to be functional).

Differential binding analyses: Peaks from all samples were merged to create a union set of sites for each transcription factor and histone mark using bedops(31). Sample-sample correlation and differential peaks analysis were performed by the CoBRA pipeline(22). Read densities were

calculated for each peak for each sample and used for the comparison of cistromes across samples. Sample similarity was determined by hierarchical clustering using the Spearman correlation between samples. differential peaks were identified by DEseq2 with adjusted $P \leq 0.05$. A total number of reads in each sample was applied to the size factor in DEseq2, which can normalize the sequencing depth between samples. Peaks from each group were used for motif analysis by the motif search findMotifsGenome.pl in HOMER (v3.0.0)(27), with cutoff q-value ≤ 1e-10.

DNA Sequencing of GEMM Tumors

Genomic DNA from the tumors indicated was isolated using the QIAamp DNA Blood Mini Kit (Qiagen #51106) according to the manufacturer's instructions. PCR was done by using KOD Xtreme™ Hot Start DNA Polymerase (Sigma-Aldrich, Cat# 71975) and following set of primers to amplify the genomic region of *Eed* targeted by sg*Eed* #2: forward *Eed #2* (5'- TACTGCTCACAGGACGAT -3'), reverse *Eed #2* (5'- GCTTCCAGCACATACCTT -3'). Two consecutive PCRs were used for *Rb1* where a 731 bp product was generated from the first PCR and subsequently used as template for the second PCR with the following sets of primers: 1st: forward *Rb1* (5'- CTATCATCTTCATGCTACAA -3'), reverse *Rb1* (5'- GCAGAATAAAATTCTACCAGG -3'), 2nd: forward *Rb1* (5'- GCATATATATCTACTTCAGCTG -3'), reverse *Rb1* (5'- GGTCAATGTGGAATACACAATTG -3').The final PCR product was then column purified using Qiagen's gel extraction kit and sequenced using next-generation Amplicon sequencing by the MGH DNA Core Facility or Sanger Sequencing (Azenta Life Sciences).

Single Cell RNA-Sequencing Sample Preparation

Single cell RNA-sequencing sample preparation was performed as described previously(8). The single-cell RNA sequencing (scRNA-seq) experiments in autochthonous CRISPR-based

EED-WT or EED-Inactivated RPR2 GEMM models were performed as previously described(32). Briefly, 3-4 month old male and female homozygous BL6J LSL-Cas9 mice (Jackson No. 026175) were intratracheally injected with EED-Inactivated or EED-WT adenoviruses (see adenovirus method above). Once mice became symptomatic from their tumors (see method above), 6 independent EED-Inactivated mice (4 EED-Inactivated RPR2 LUADs and 2 EED-Inactivated RPR2 SCLCs) and 3 independent EED WT mice were euthanized and lung tumors dissected and finely minced mechanically using a razor blade and then enzymatically digested with Mouse Tumor Dissociation Kit (Miltenyi Biotec, #130-096-730) following the manufacturer's instruction. Briefly, minced tumor tissue was transferred to a gentleMACS C Tube containing enzyme mix prepared with 20% of Enzyme R option to preserve cell surface epitopes. Dissociation using the gentleMACS Octo Dissociator with Heaters (Miltenyi Biotec, #130-096-427) was performed using the 37C_m_TDK_2 gentleMACS Program. The single cell suspensions were resuspended in RPMI containing 10% FBS and subsequently passed through a 70 µm Cell Strainer (Greiner, #542070) and centrifuged at 300 x g for 3 min followed by 2 washes with 0.04% UltraPure Bovine Serum Albumin (Invitrogen, AM2616) in DPBS. Finally, dissociated cells were resuspended in DPBS with 0.04% UltraPure BSA and cell counts were measured with a Vi-CELL XR Cell Viability Analyzer (Beckman Coulter). Cells were then diluted in 0.04% BSA/DPBS at a cell concentration of 1000 cells/µL. About 16,000 cells were loaded onto a 10x Genomics Chromium™ instrument (10x Genomics) according to the manufacturer's instructions. The scRNA-seq libraries were processed using Chromium Next GEM Single Cell 5' Kit v2 kit (10x Genomics). Quality controls for amplified cDNA libraries and final sequencing libraries were performed using Bioanalyzer High Sensitivity DNA Kit (Agilent). The sequencing libraries for scRNA-seq were normalized to 4 nM concentration and pooled. The pooled sequencing libraries were sequenced on Illumina NovaSeq S4 300 cycle platform. The sequencing parameters used were: Read 1 of 26bp, Read 2 of 90bp, Index 1 of 10bp and Index 2 of 10bp. EED WT RPR2 scRNA-seq data from 3 independent mice were previously published(32).

Single Cell RNA-Sequencing Data Analysis

Single cell RNA-Sequencing data analyses was performed as described previously(8). Custom reference genome was established by adding *EGFP* and *Cas9* sequence(33) to the mouse genome (mm10-2020-A). Cell ranger version 6.0.2 pipeline (10x Genomics) was used to align sequencing data to the custom mouse reference genome and generate the gene-level counts matrix. Unfiltered raw counts data was imported into Seurat v4 R package (version 4.1.0)(34) for downstream processing and analysis. Low quality cells were filtered out using following thresholds: total UMI counts < 500, number of transcripts < 350, $\log_{10}$TranscriptsPerUMI <= 0.8, and cells with more than 15% transcripts mapping to mitochondrial genes. In addition, genes expressed in less than ten cells were removed. The UMI counts matrices were then natural-log normalized and scaled with Seurat's 'NormalizeData' and 'ScaleData' functions.

Single Nuclei RNA-Sequencing Sample Preparation

The single-nuclei RNA sequencing (snRNA-seq) sample preparation from EED-Inactivated RPR2 LUAD tumors were performed using Chromium Nuclei Isolation Kit with RNase Inhibitor (10x Genomics, PN-1000494) following manufacturer's instruction (10x Genomics, CG000124). Briefly, 30 mg snap-frozen tumor tissue from 3 independent EED-Inactivated RPR2 mice was dissociated in Lysis Buffer with pestle and centrifuged for 20s, in 16,000 rcf under 4 ºC to a column to collect lysed nuclei in the flowthrough. Debris in the flowthrough containing nuclei was removed by Debris Removal Buffer before nuclei were washed 2 times with 5 min, 500 rcf, 4 ºC spin. Nuclei was then counted using ViaStain AOPI Staining Solution (Revvity, CS2-0106-5ML) on a Nexcelom Cellometer K2 Fluorescent Cell Counter (Revvity, CMT-K2-MX-150) and resuspended in 1,000 nuclei/ul DPBS with 0.04% ultra-pure Bovine Serum Albumin (Invitrogen, AM2616). About 20,000 nuclei were loaded onto a ChromiumTM X instrument (10x Genomics) according to the manufacturer's instructions. The snRNAseq libraries were processed using Chromium GEM-

X Single Cell 5' Kit v3 (10x Genomics). Quality controls for amplified cDNA libraries and final sequencing libraries were performed using Bioanalyzer High Sensitivity DNA Kit (Agilent). Libraries were subsequently quantified by Qubit fluorometer and Agilent 4200 TapeStation system. Library pooling and indexing was evaluated with shallow sequencing on an Illumina MiSeq. Subsequently, libraries were sequenced by the Molecular Biology Core Facilities at Dana-Farber Cancer Institute on an Illumina NovaSeq X Plus with the following sequencing parameters: 28bp, 10bp, 10bp, 90bp.

Single-Nuclei RNA Sequencing Data Analysis

Custom reference genome was established by adding *EGFP* and *Cas9* sequence(33) to the mouse genome (mm10-2020-A). Cell ranger version 9.0.0 pipeline (10x Genomics) was used to align sequencing data to the custom mouse reference genome and generate the gene-level counts matrix. Unfiltered raw counts data was imported into Seurat v4 R package (version 4.1.0)(34) for downstream processing and analysis. Low quality cells were filtered out using following thresholds: total UMI counts < 500, number of transcripts < 375, $\log_{10}$TranscriptsPerUMI <= 0.8, mitochondrial gene ratio >= 0.2 for scRNA-seq and >= 0.1 for snRNA-seq. In addition, genes expressed in less than five cells were removed. The UMI counts matrices were then natural-log normalized and scaled with Seurat's 'NormalizeData' and 'ScaleData' functions.

Dimension Reduction, Cluster Analysis and Visualization of scRNA-Seq and snRNA-Seq Data

The Seurat v4 R package was used for dimension reduction and clustering. Top 2,000 genes with the highest variance were selected using the 'FindVariableFeatures' function with 'vst' method to perform linear dimensional reduction (principal component analysis) using the 'RunPCA' function, and top 40 principal components for scRNA-seq and top 25 principal components for snRNA-seq were used to perform graph-based unsupervised clustering with the

‘FindNeighbors’ and ‘FindClusters’ functions and Uniform Manifold Approximation and Projection for Dimension Reduction (UMAP) (arXiv:1802.03426) for data visualization in two-dimensional space. Automatic cluster annotation using the R package SingleR (version 1.8.1)(35) with ImmGenData(36, 37) and manual tumor cluster annotation with markers of *EGFP* and *Cas9* were performed to select the tumor population. Subsequently, *Ptprc* positive tumor cells, *Cas9* and *EGFP* positive tumor microenvironment were filtered out. Then, the tumor subpopulations were reanalyzed. Seurat’s ‘CellCycleScoring’ function and S phase and G2/M phase specific gene lists (Supplemental Table 4, Tab 4) were used to calculate the G2M and S phase gene expression scores and to be regressed out using the ‘var.to.regress’ option in the ‘SCTransform’ function with default parameters. Top 3,000 genes excluding mitochondrial and ribosomal genes with the highest variance were selected using the ‘FindVariableFeatures’ function with ‘vst’ method to perform linear dimensional reduction (principal component analysis) using the ‘RunPCA’ function, and top 40 principal components were used to plot UMAP for data visualization in two-dimensional space in scRNA-seq and top 9 principal components were used in snRNA-seq. Differential expression profile were obtained with Seurat’s ‘FindMarkers’ function using test.use = “wilcox” to compare differentially expressed genes.

Cell trajectory analysis was performed on tumor populations with the Monocle2 package (V2.24.0). For snRNA-seq, top 100 of each of differentially upregulated and downregulated genes between the *Neurod1*+ cluster and *Muc13*+ cluster were used as the ordering genes. For scRNA-seq, top 300 differentially expressed genes between EED-Inactivated RPR2 LUAD tumor cells and EED-WT RPR2 SCLC tumor cells were used as ordering genes. To predict cell biological trajectories, Monocle2 estimated size factor and dispersion, followed by reduceDimension function with the “DDRTree” method to reduce the high-dimensional space as a way to infer cells progression through a given biological process. For snRNA-seq, top 300 non-mitochondrial genes that significantly correlates to the trajectory (based on descending order of q value from “differentialGeneTest” function) were plotted in the heatmap in Figure 4H.

Tumor and its Microenvironment Communication Analysis using Differential NicheNet

Tumor and its microenvironment communication analysis using Differential NicheNet was performed as described previously(8). To study communication between the tumor and cells of the microenvironment in EED-WT SCLC, EED-Inactivated SCLC, and EED-Inactivated LUAD tumors, we used the nichenetr package V 1.1.0.39. NicheNet predicts which differentially expressed ligands from one tumor microenvironment population are most likely to affect target gene expression of tumor population and which specific target genes are affected by which of these predicted ligands. It uses curated ligand-receptor and ligand-target databases(38) as references to evaluate the cellular communication networks between two cell types. Human gene names were converted to their one-to-one orthologs using 'convert_human_to_mouse_symbols' function. Ligands, receptors, and targets that expressed in at least 10% (default cutoff) of the single cells in a specific cluster were included. Differential genesets of interest were calculated using a log transformed fold change threshold of 0.15 (recommended cutoff for 10x datasets), and 2889 genes were included in EED-WT SCLC and 1668 genes of interest were included in EED-Inactivated LUAD. Next, ligand-receptor-target links were prioritized using the weights of scaled properties of interest as follows: "scaled_ligand_score" = 5, "scaled_ligand_expression_scaled" = 1, "ligand_fraction" = 1, "scaled_ligand_score_spatial" = 0, "scaled_receptor_score" = 0.5, "scaled_receptor_expression_scaled" = 0.5, "receptor_fraction" = 1, "ligand_scaled_receptor_expression_fraction" = 1, "scaled_receptor_score_spatial" = 0, "scaled_activity" = 0, "scaled_activity_normalized" = 1, "bona_fide" = 1. Confident Tumor-TME interactions that are significantly enriched in EED-Inactivated LUAD were further identified by significantly different expression of ligand in TME and receptor in tumors with Bonafide interaction. Tumor-expressing receptors in significantly enriched interaction pairs were further overlapped with significantly upregulated genes (p value<0.05) in mouse SCLC RPR2 cell line 1014 after EED genetic inactivation. Scaled receptor score (Log2FoldChange comparing EED-Inactivated

LUAD and EED-WT SCLC) of unique receptors that are 1. Directly activated by loss of EED, 2, predicted activated by ligand from TME only in EED-Inactivated LUAD, were plotted in Figure 5I.

CNV Inference and Phylogenetic Tree Construction

R package inferCNV (1.16.0) was used to detect copy number variations from snRNA-seq data. Normal pulmonary epithelium and stroma cells were used as reference for the tumor population from the corresponding sample. The following parameters in the "run" function of infercnv were used: cutoff = 0.1, denoise = TRUE, cluster_by_groups = T, HMM = T. All genes from HMM copy number inferences (from file 17_HMM_predHMMi6.hmm_mode-samples.pred_cnv_genes.dat) were used to construct the phylogenetic tree; copy number was set to diploid if a cluster did not have an inferred CNV event predicted. Phylogenetic trees were inferred using the Maximum Parsimony (MP) method with the Subtree-Pruning-Regrafting (SPR) algorithm(39) with search level 1 in MEGA11(40).

Flow Cytometry

Flow cytometry was performed as described previously(8). Fresh single cell suspensions from EED WT or EED-Inactivated RPR2 mice tumors were prepared, washed twice in PBS, resuspended in FACS buffer (D-PBS containing 2% FBS) and transferred in 1.5 mL Eppendorf tubes. After washing, cells were incubated with fluorophore conjugated anti-H-2Ld/H-2Db (Biolegend #114507, clone 28-14-8) or the isotype control (Biolegend #400212, IgG2a, κ isotype Ctrl) at 1:100 dilution in the dark for 30 minutes at room temperature. After washing, cells were resuspended in FACS buffer, transferred to flow cytometry tubes containing a 40 μm filter and analyzed on a LSR Fortessa flow cytometer (Becton Dickinson). Data analyses were performed with FlowJo software (V10).

Whole Exome Sequencing of GEMM Tumors

Genomic DNA of the indicated tumors and normal lung tissue were extracted using the QIAamp DNA Blood Mini Kit (Qiagen #51106) according to the manufacturer's instructions and randomly sheared into short fragments with the size of 180-280 bp. The obtained fragments were end repaired, A-tailed, and further ligated with Illumina adapters and sequenced on Illumina NovaSeq 6000 with PE150 strategy. FASTQ raw sequences passing quality control with fastp (v0.23.1) were aligned to mouse mm10 reference genome with Burrows-Wheeler Aligner (BWA, v0.7.17), and Sambamba(41) (v1.0.0) was used to sort the aligned BAM files, and Picard tools (v2.18.9) (https://broadinstitute.github.io/picard/) were then utilized with ASSUME_SORTED=true,CREATE_INDEX=true to merge BAM files and mark duplicate reads. Somatic mutations of tumors comparing to normal lung tissue were detected by MuTect (v2.2-25-g2a68eab)(42). Control-FREEC (v11.4)(43) was used to detect somatic CNV. The parameter with window = 0 was set as officially recommended in the config file for somatic CNV detection. Germline Indel was detected with GATK (v4.3.0)(44) and VariantFiltration module was used to filter germline InDel. The filter parameters are: QD < 2.0 || FS > 200.0 || ReadPosRankSum < -20.0. ANNOVAR(45) was used to perform variant annotation. Somatic copy number variations and non-synonymous mutations of LUAD oncogenic drivers(46, 47) were plotted using oncoprint() function in ComplexHeatmaps package (v2.18.0)(48) in R.

EED cDNA for Exogenous EED Re-Expression

pTwist Lenti SFFV Puro WPRE lentiviral vectors encoding: 1) EED WT-HA or 2) EED Triple Mutant-HA (F97A/Y148A/Y365A) which encodes a functionally inactive EED Mutant-HA were codon optimized and synthesized by Twist Biosciences. All plasmids were sequence verified by sanger sequencing. The functionally inactive EED Mutant was confirmed as was unable to restore H3K27me3 upon re-expression relative to EED-WT which restored H3K27me3 upon re-expression.

References

1. Oh S, Koh J, Kim TM, Kim S, Youk J, Kim M, et al. Transcriptomic Heterogeneity of EGFR-Mutant Non-Small Cell Lung Cancer Evolution Toward Small-Cell Lung Cancer. *Clin Cancer Res.* 2024;30(20):4729-42.
2. Quintanal-Villalonga A, Taniguchi H, Zhan YA, Hasan MM, Chavan SS, Meng F, et al. Multiomic Analysis of Lung Tumors Defines Pathways Activated in Neuroendocrine Transformation. *Cancer Discov.* 2021;11(12):3028-47.
3. Guo M, Tomoshige K, Meister M, Muley T, Fukazawa T, Tsuchiya T, et al. Gene signature driving invasive mucinous adenocarcinoma of the lung. *EMBO Mol Med.* 2017;9(4):462-81.
4. Wagle MC, Kirouac D, Klijn C, Liu B, Mahajan S, Junttila M, et al. A transcriptional MAPK Pathway Activity Score (MPAS) is a clinically relevant biomarker in multiple cancer types. *NPJ Precis Oncol.* 2018;2(1):7.
5. Cerami E, Gao J, Dogrusoz U, Gross BE, Sumer SO, Aksoy BA, et al. The cBio cancer genomics portal: an open platform for exploring multidimensional cancer genomics data. *Cancer Discov.* 2012;2(5):401-4.
6. Oser MG, Sabet AH, Gao W, Chakraborty AA, Schinzel AC, Jennings RB, et al. The KDM5A/RBP2 histone demethylase represses NOTCH signaling to sustain neuroendocrine differentiation and promote small cell lung cancer tumorigenesis. *Genes Dev.* 2019;33(23-24):1718-38.
7. Hong D, Knelson EH, Li Y, Durmaz YT, Gao W, Walton E, et al. Plasticity in the Absence of NOTCH Uncovers a RUNX2-Dependent Pathway in Small Cell Lung Cancer. *Cancer Res.* 2021.
8. Li Y, Mahadevan NR, Duplaquet L, Hong D, Durmaz YT, Jones KL, et al. Aurora A kinase inhibition induces accumulation of SCLC tumor cells in mitosis with restored interferon signaling to increase response to PD-L1. *Cell Rep Med.* 2023;4(11):101282.
9. DuPage M, Dooley AL, and Jacks T. Conditional mouse lung cancer models using adenoviral or lentiviral delivery of Cre recombinase. *Nat Protoc.* 2009;4(7):1064-72.
10. Foggetti G, Li C, Cai H, Hellyer JA, Lin WY, Ayeni D, et al. Genetic Determinants of EGFR-Driven Lung Cancer Growth and Therapeutic Response In Vivo. *Cancer Discov.* 2021;11(7):1736-53.
11. Oser MG, Fonseca R, Chakraborty AA, Brough R, Spektor A, Jennings RB, et al. Cells Lacking the RB1 Tumor Suppressor Gene Are Hyperdependent on Aurora B Kinase for Survival. *Cancer Discov.* 2019;9(2):230-47.
12. Subramanian A, Tamayo P, Mootha VK, Mukherjee S, Ebert BL, Gillette MA, et al. Gene set enrichment analysis: a knowledge-based approach for interpreting genome-wide expression profiles. *Proc Natl Acad Sci U S A.* 2005;102(43):15545-50.
13. Liberzon A, Birger C, Thorvaldsdottir H, Ghandi M, Mesirov JP, and Tamayo P. The Molecular Signatures Database (MSigDB) hallmark gene set collection. *Cell Syst.* 2015;1(6):417-25.
14. Borromeo MD, Savage TK, Kollipara RK, He M, Augustyn A, Osborne JK, et al. ASCL1 and NEUROD1 Reveal Heterogeneity in Pulmonary Neuroendocrine Tumors and Regulate Distinct Genetic Programs. *Cell Rep.* 2016;16(5):1259-72.
15. Nabet BY, Hamidi H, Lee MC, Banchereau R, Morris S, Adler L, et al. Immune heterogeneity in small-cell lung cancer and vulnerability to immune checkpoint blockade. *Cancer Cell.* 2024;42(3):429-43 e4.
16. Kanehisa M, Furumichi M, Sato Y, Matsuura Y, and Ishiguro-Watanabe M. KEGG: biological systems database as a model of the real world. *Nucleic Acids Res.* 2025;53(D1):D672-D7.

17. Su AI, Wiltshire T, Batalov S, Lapp H, Ching KA, Block D, et al. A gene atlas of the mouse and human protein-encoding transcriptomes. *Proc Natl Acad Sci U S A.* 2004;101(16):6062-7.
18. Gene Ontology C, Aleksander SA, Balhoff J, Carbon S, Cherry JM, Drabkin HJ, et al. The Gene Ontology knowledgebase in 2023. *Genetics.* 2023;224(1).
19. Chen EY, Tan CM, Kou Y, Duan Q, Wang Z, Meirelles GV, et al. Enrichr: interactive and collaborative HTML5 gene list enrichment analysis tool. *BMC Bioinformatics.* 2013;14:128.
20. Hong D, Lyu Y, Nayak R, Becker JS, Booker MA, Masuzawa K, et al. Loss of NOTCH2 creates a TRIM28-dependent vulnerability in small cell lung cancer. *Dev Cell.* 2025;60(24):3462-79 e13.
21. Taing L, Bai G, Cousins C, Cejas P, Qiu X, Herbert Z, et al. CHIPS: A Snakemake pipeline for quality control and reproducible processing of chromatin profiling data [version 1; peer review: 1 not approved]. *F1000Research.* 2021;10(517).
22. Qiu X, Feit AS, Feiglin A, Xie Y, Kesten N, Taing L, et al. CoBRA: Containerized Bioinformatics Workflow for Reproducible ChIP/ATAC-seq Analysis. *Genomics, proteomics & bioinformatics.* 2021;19(4):652-61.
23. Li H, and Durbin R. Fast and accurate short read alignment with Burrows-Wheeler transform. *Bioinformatics.* 2009;25(14):1754-60.
24. Zhang Y, Liu T, Meyer CA, Eeckhoute J, Johnson DS, Bernstein BE, et al. Model-based analysis of ChIP-Seq (MACS). *Genome biology.* 2008;9(9):R137-R.
25. Robinson JT, Thorvaldsdottir H, Winckler W, Guttman M, Lander ES, Getz G, et al. Integrative genomics viewer. *Nat Biotechnol.* 2011;29(1):24-6.
26. Ross-Innes CS, Stark R, Teschendorff AE, Holmes KA, Ali HR, Dunning MJ, et al. Differential oestrogen receptor binding is associated with clinical outcome in breast cancer. *Nature.* 2012;481(7381):389-93.
27. Heinz S, Benner C, Spann N, Bertolino E, Lin YC, Laslo P, et al. Simple combinations of lineage-determining transcription factors prime cis-regulatory elements required for macrophage and B cell identities. *Molecular cell.* 2010;38(4):576-89.
28. Ramirez F, Ryan DP, Gruning B, Bhardwaj V, Kilpert F, Richter AS, et al. deepTools2: a next generation web server for deep-sequencing data analysis. *Nucleic Acids Res.* 2016;44(W1):W160-5.
29. El Zarif T, Meador CB, Qiu X, Seo JH, Davidsohn MP, Savignano H, et al. Detecting Small Cell Transformation in Patients with Advanced EGFR Mutant Lung Adenocarcinoma through Epigenomic cfDNA Profiling. *Clin Cancer Res.* 2024;30(17):3798-811.
30. Li H, and Durbin R. Fast and accurate long-read alignment with Burrows-Wheeler transform. *Bioinformatics.* 2010;26(5):589-95.
31. Neph S, Kuehn MS, Reynolds AP, Haugen E, Thurman RE, Johnson AK, et al. BEDOPS: high-performance genomic feature operations. *Bioinformatics.* 2012;28(14):1919-20.
32. Duplaquet L, Li Y, Booker MA, Xie Y, Olsen SN, Patel RA, et al. KDM6A epigenetically regulates subtype plasticity in small cell lung cancer. *Nat Cell Biol.* 2023;25(9):1346-58.
33. Platt RJ, Chen S, Zhou Y, Yim MJ, Swiech L, Kempton HR, et al. CRISPR-Cas9 knockin mice for genome editing and cancer modeling. *Cell.* 2014;159(2):440-55.
34. Hao Y, Hao S, Andersen-Nissen E, Mauck WM, 3rd, Zheng S, Butler A, et al. Integrated analysis of multimodal single-cell data. *Cell.* 2021;184(13):3573-87 e29.
35. Aran D, Looney AP, Liu L, Wu E, Fong V, Hsu A, et al. Reference-based analysis of lung single-cell sequencing reveals a transitional profibrotic macrophage. *Nat Immunol.* 2019;20(2):163-72.

36. Heng TS, Painter MW, and Immunological Genome Project C. The Immunological Genome Project: networks of gene expression in immune cells. *Nat Immunol.* 2008;9(10):1091-4.
37. Benayoun BA, Pollina EA, Singh PP, Mahmoudi S, Harel I, Casey KM, et al. Remodeling of epigenome and transcriptome landscapes with aging in mice reveals widespread induction of inflammatory responses. *Genome Res.* 2019;29(4):697-709.
38. Browaeys R, Saelens W, and Saeys Y. NicheNet: modeling intercellular communication by linking ligands to target genes. *Nat Methods.* 2020;17(2):159-62.
39. Nei M, and Kumar S. *Molecular evolution and phylogenetics.* Oxford ; New York: Oxford University Press; 2000.
40. Tamura K, Stecher G, and Kumar S. MEGA11: Molecular Evolutionary Genetics Analysis Version 11. *Mol Biol Evol.* 2021;38(7):3022-7.
41. Tarasov A, Vilella AJ, Cuppen E, Nijman IJ, and Prins P. Sambamba: fast processing of NGS alignment formats. *Bioinformatics.* 2015;31(12):2032-4.
42. Cibulskis K, Lawrence MS, Carter SL, Sivachenko A, Jaffe D, Sougnez C, et al. Sensitive detection of somatic point mutations in impure and heterogeneous cancer samples. *Nat Biotechnol.* 2013;31(3):213-9.
43. Boeva V, Popova T, Bleakley K, Chiche P, Cappo J, Schleiermacher G, et al. Control-FREEC: a tool for assessing copy number and allelic content using next-generation sequencing data. *Bioinformatics.* 2012;28(3):423-5.
44. DePristo MA, Banks E, Poplin R, Garimella KV, Maguire JR, Hartl C, et al. A framework for variation discovery and genotyping using next-generation DNA sequencing data. *Nat Genet.* 2011;43(5):491-8.
45. Wang K, Li M, and Hakonarson H. ANNOVAR: functional annotation of genetic variants from high-throughput sequencing data. *Nucleic Acids Res.* 2010;38(16):e164.
46. Jordan EJ, Kim HR, Arcila ME, Barron D, Chakravarty D, Gao J, et al. Prospective Comprehensive Molecular Characterization of Lung Adenocarcinomas for Efficient Patient Matching to Approved and Emerging Therapies. *Cancer Discov.* 2017;7(6):596-609.
47. Skoulidis F, and Heymach JV. Co-occurring genomic alterations in non-small-cell lung cancer biology and therapy. *Nat Rev Cancer.* 2019;19(9):495-509.
48. Gu Z, Eils R, and Schlesner M. Complex heatmaps reveal patterns and correlations in multidimensional genomic data. *Bioinformatics.* 2016;32(18):2847-9.
