# Supplementary material for: Polycomb scaffolding subunit EED supports the SCLC neuroendocrine identity and EGFR-mutant LUAD-to-SCLC transformation
Source: J Clin Invest. 2026 Aug 18;136(20):e199454. doi: 10.1172/JCI199454 (PMC13668632; doi:10.1172/JCI199454)

Unedited blots of “EED Maintains the Small Cell Lung Cancer Neuroendocrine Phenotype and Is Necessary for Lung Cancer Histological Transformation”

199454-JCI-RG-DN-3

Li et al.

Full unedited immunoblots for Figure 3I

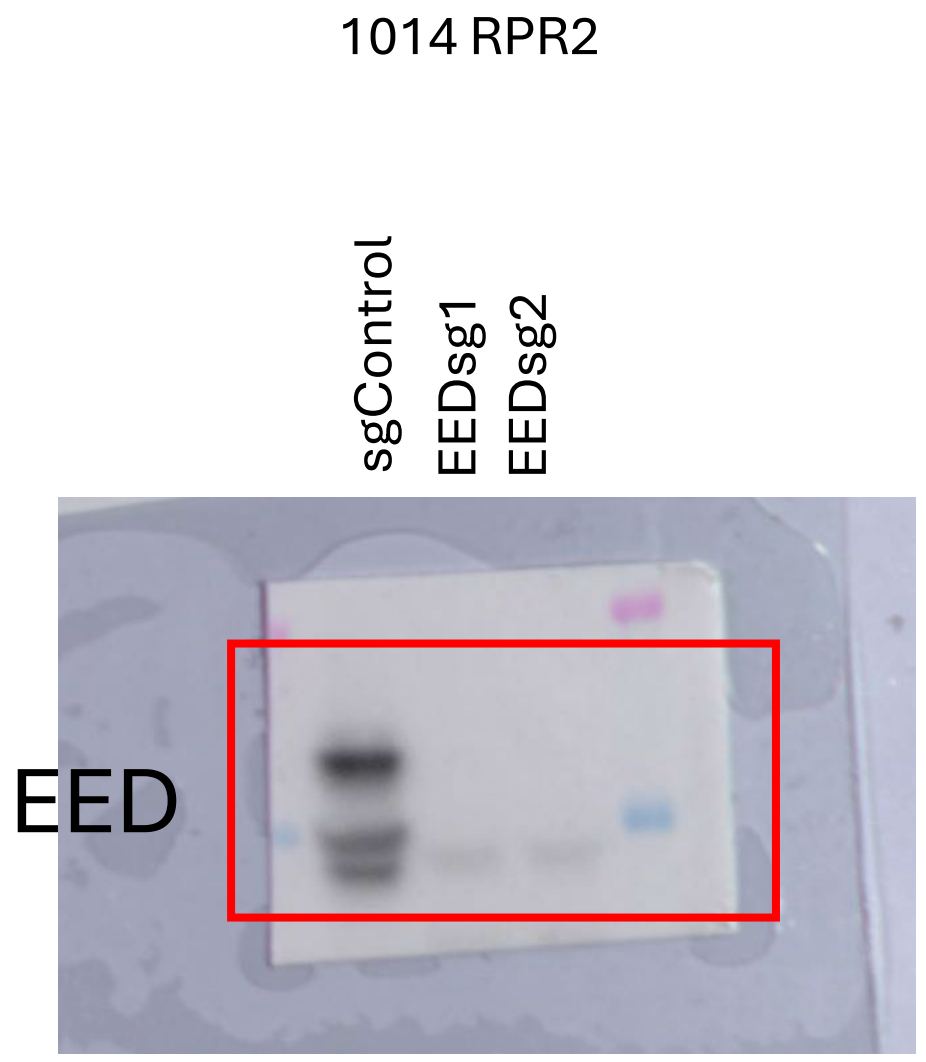

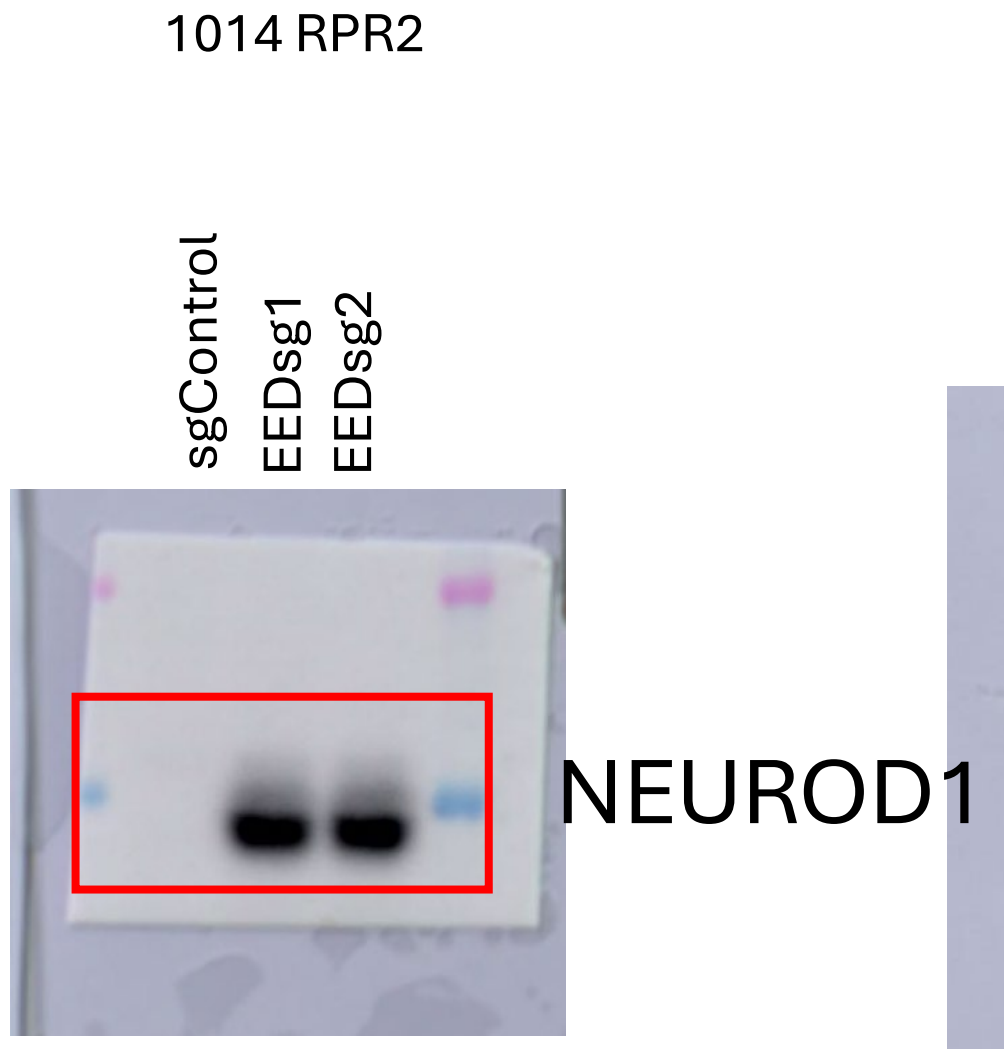

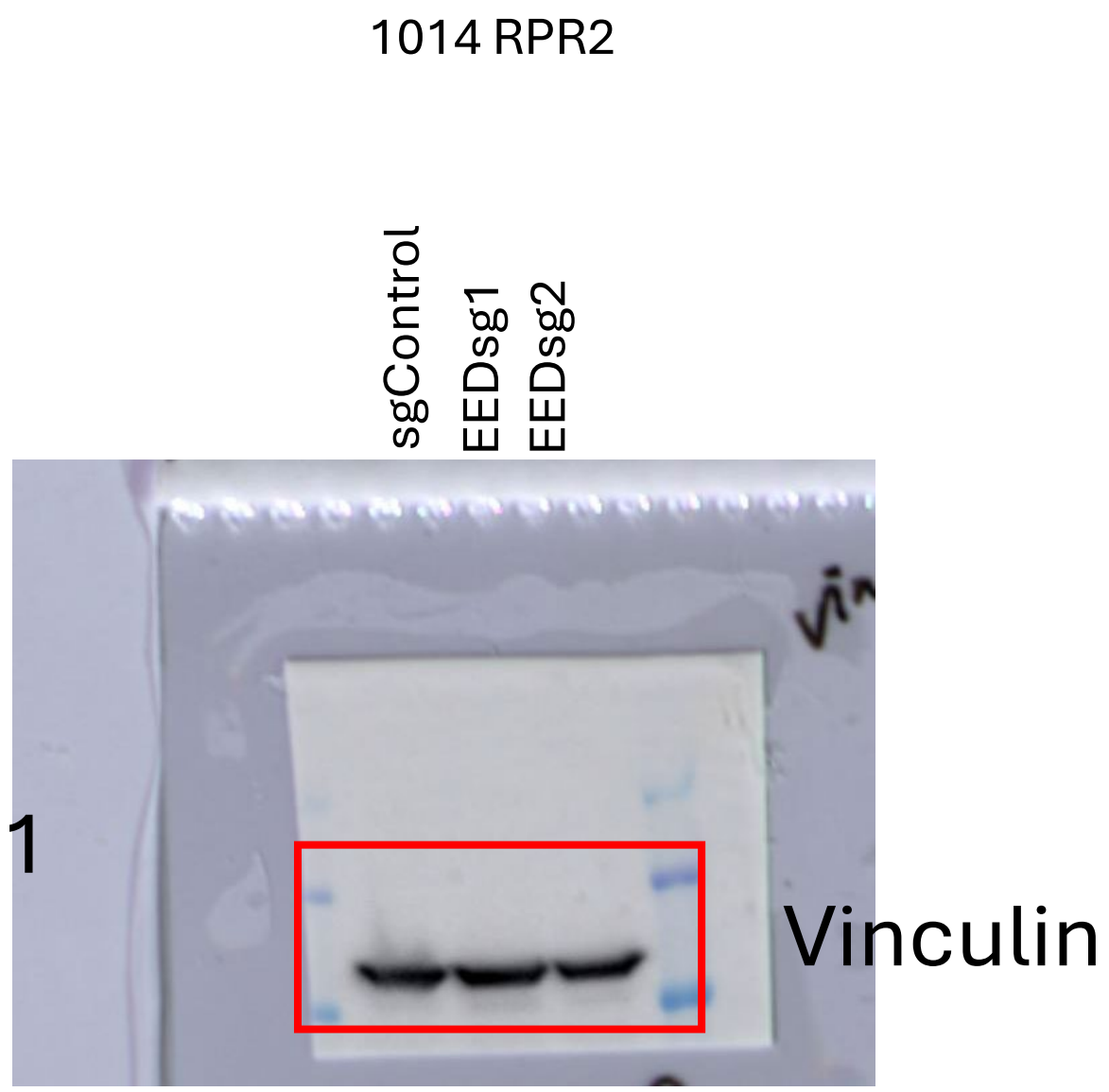

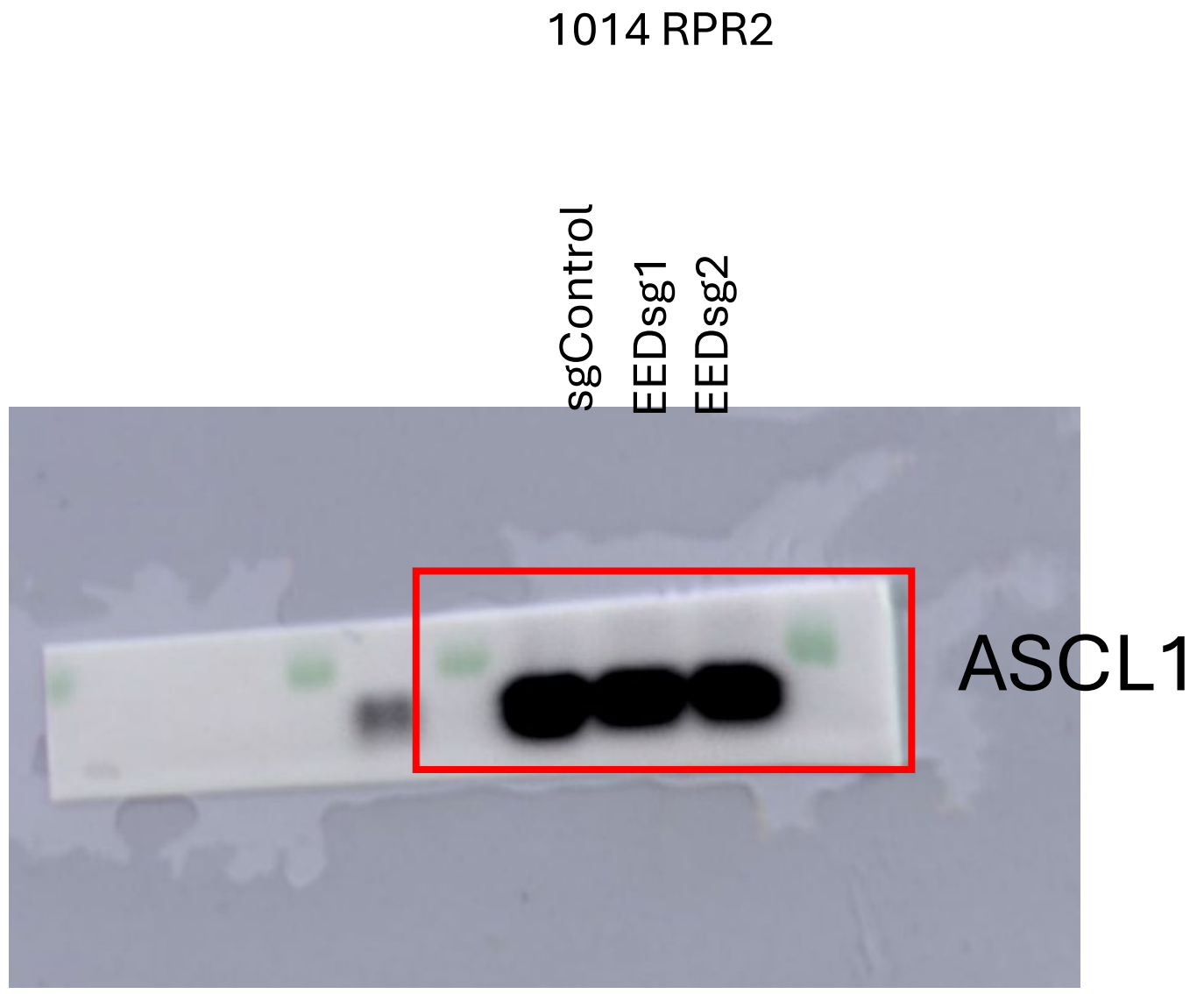

Full unedited immunoblots for Figure 5C

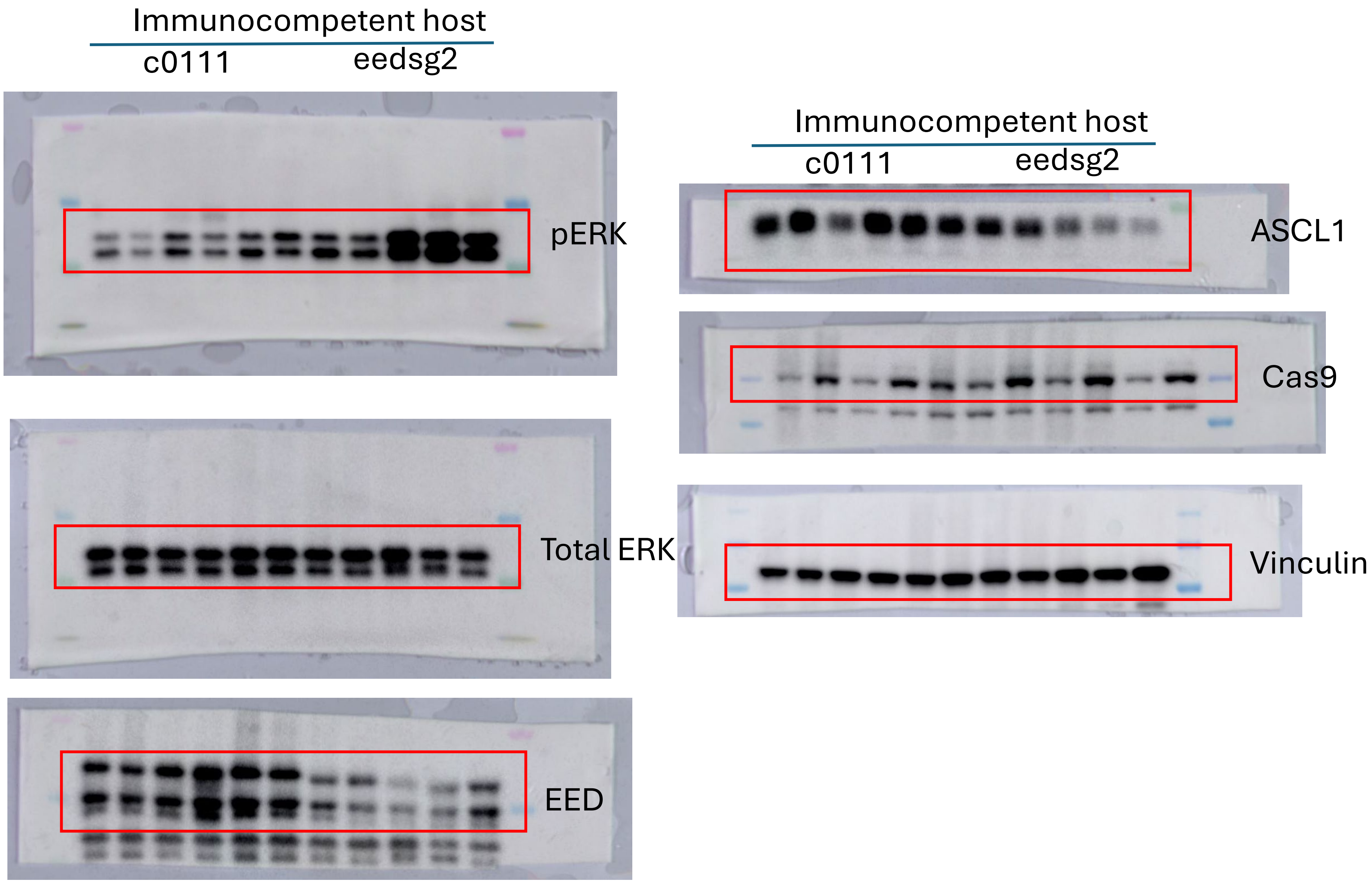

Full unedited immunoblots for Figure 5C continued

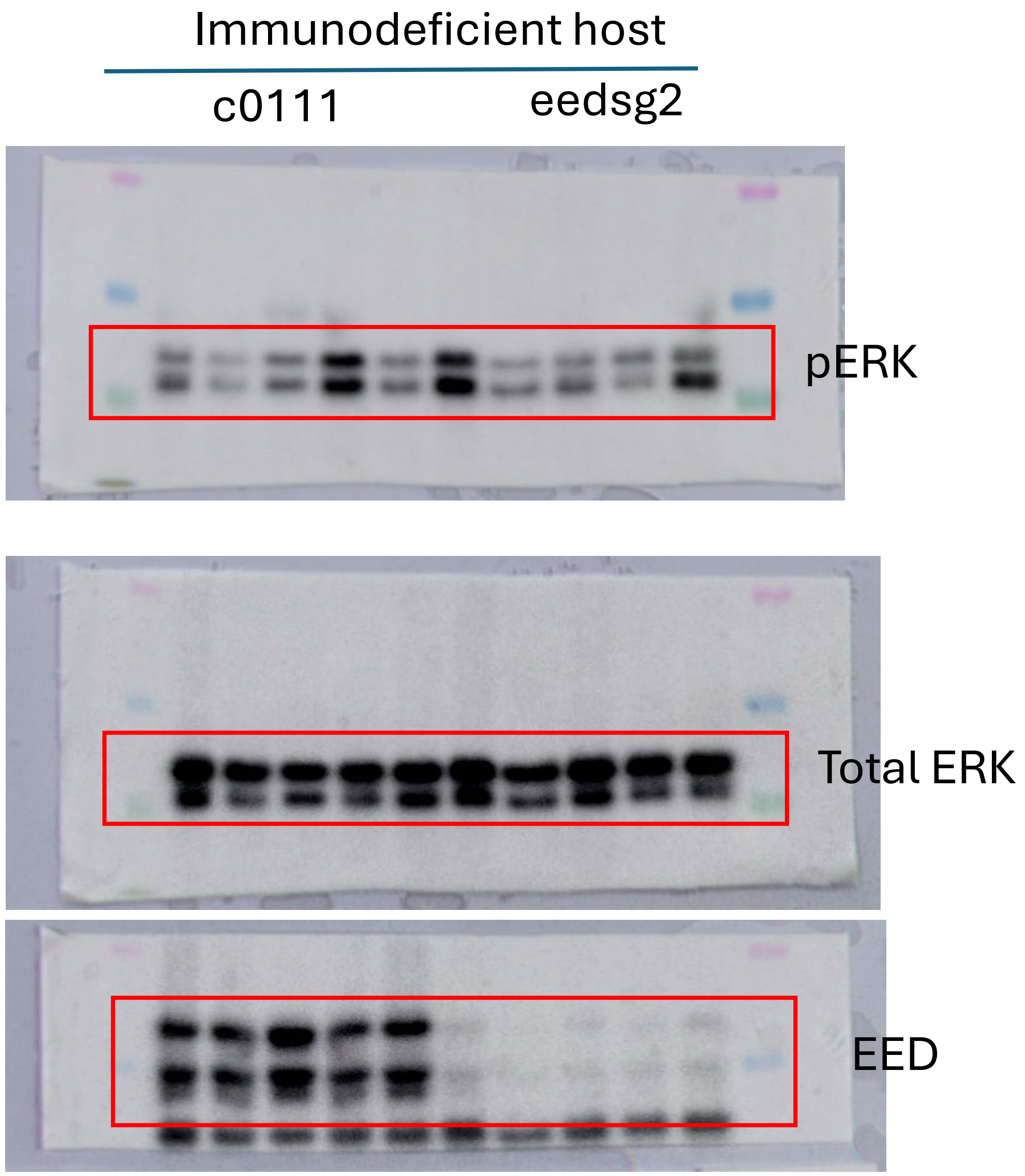

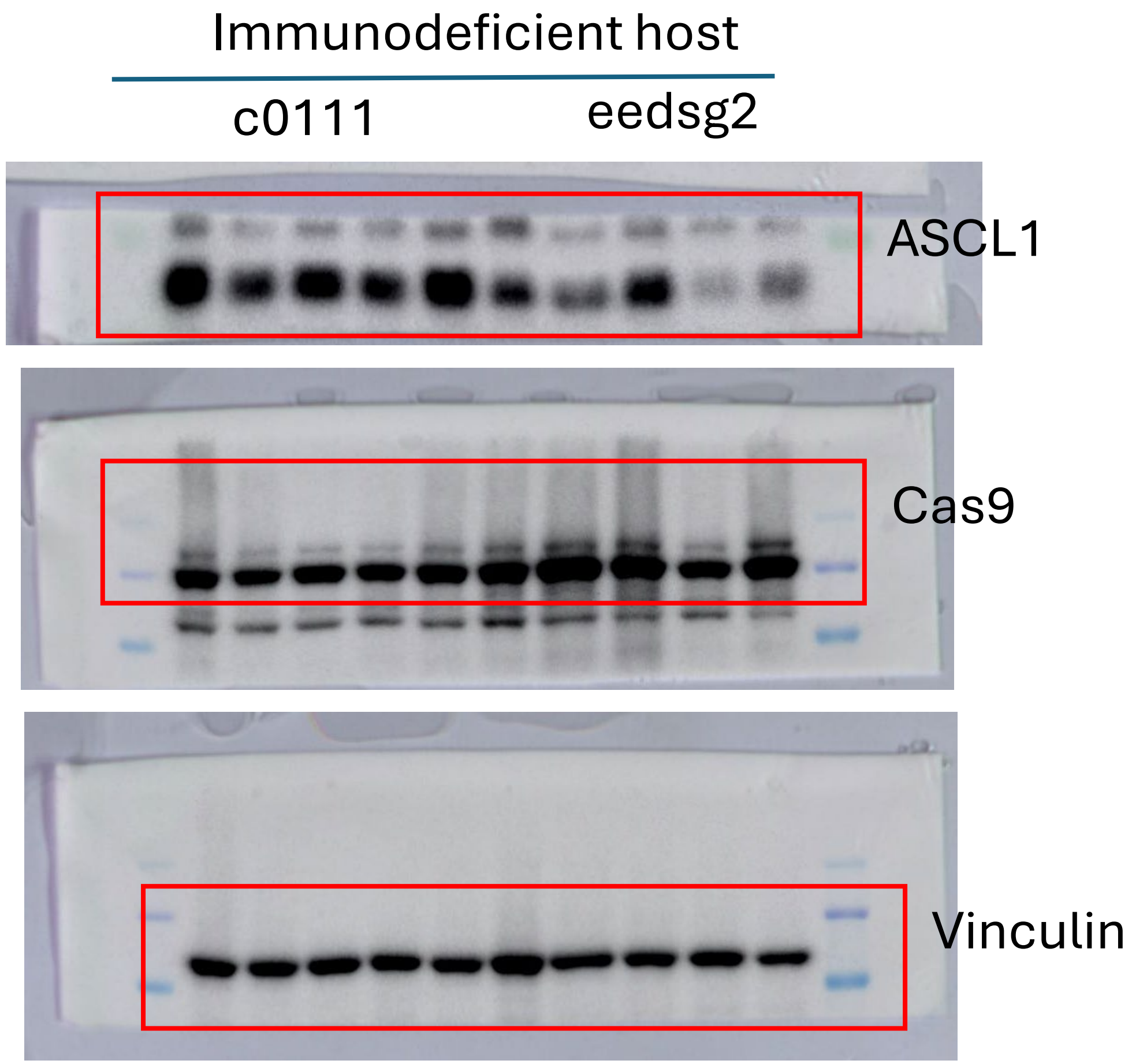

Full unedited immunoblots for Figure 6D

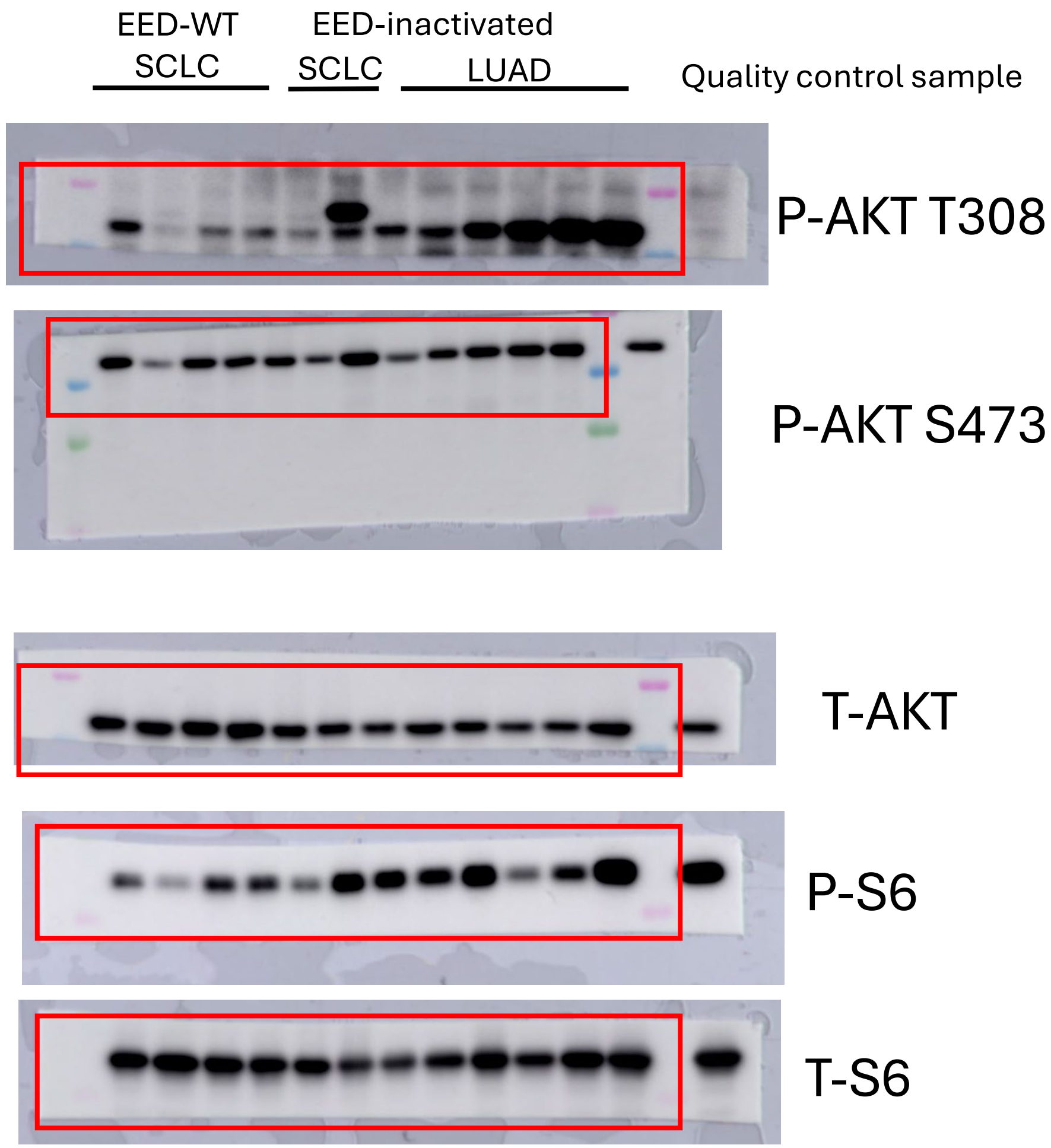

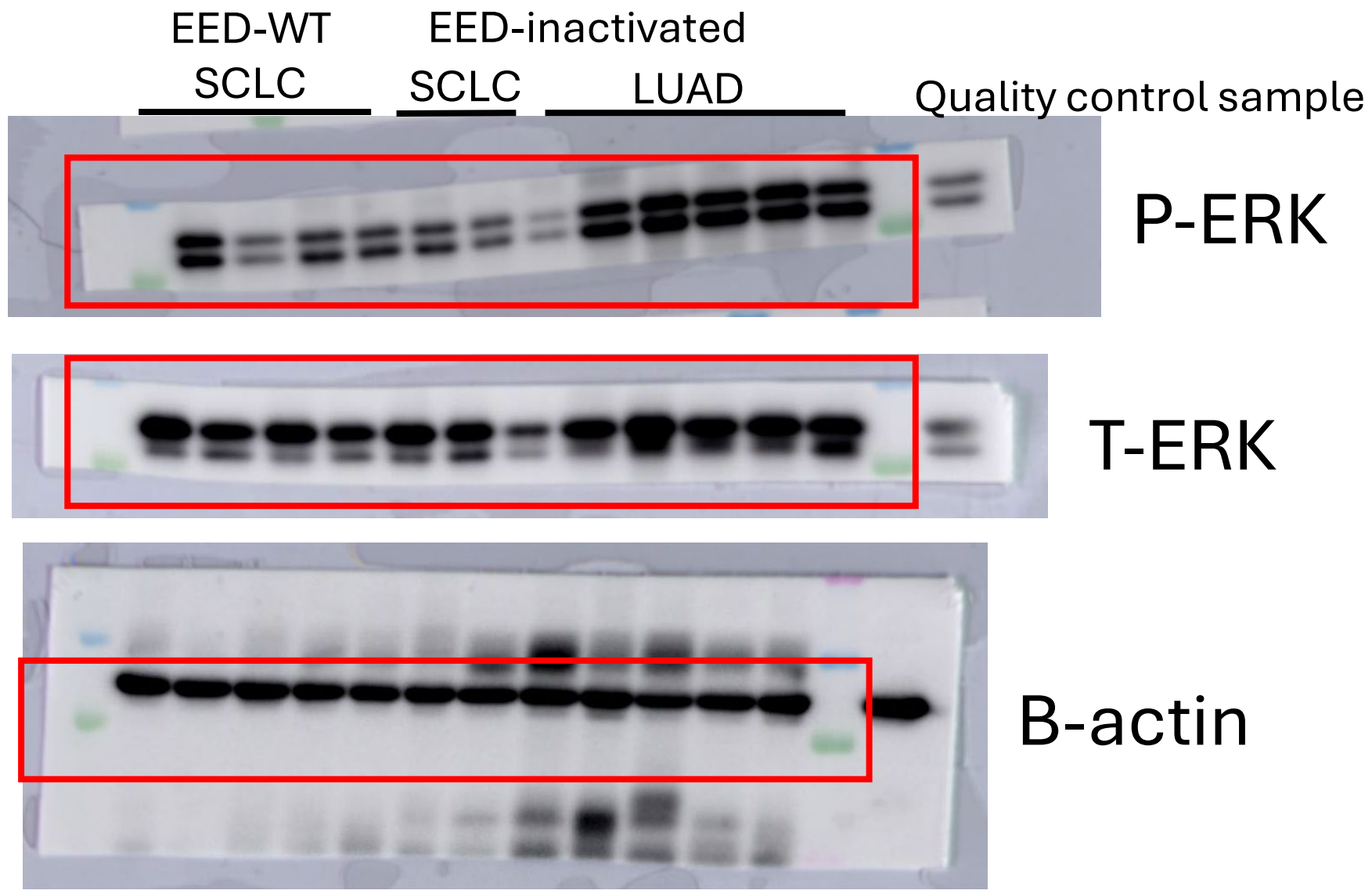

Full unedited immunoblots for Figure 6H

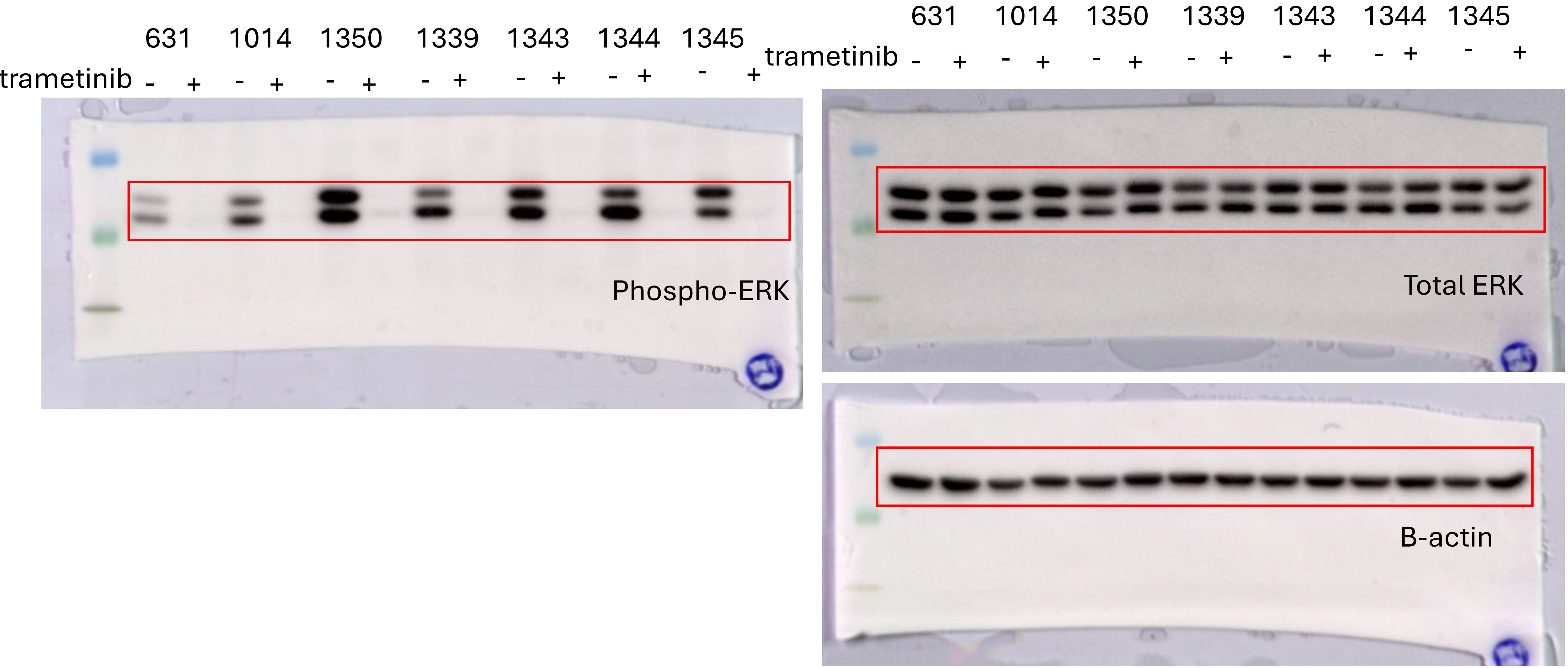

Full unedited immunoblots for Figure 8H

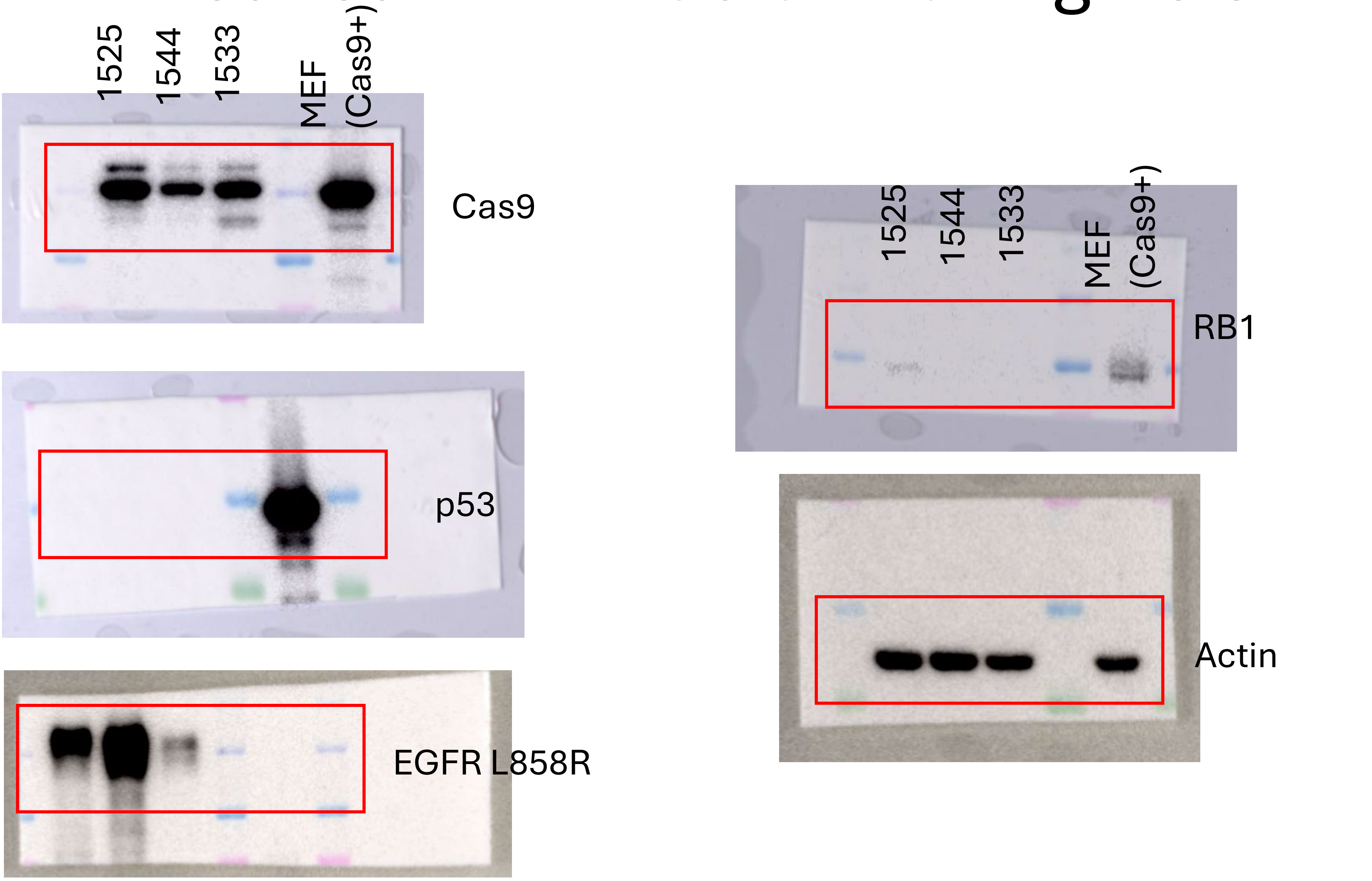

Full unedited immunoblots for Figure 8I

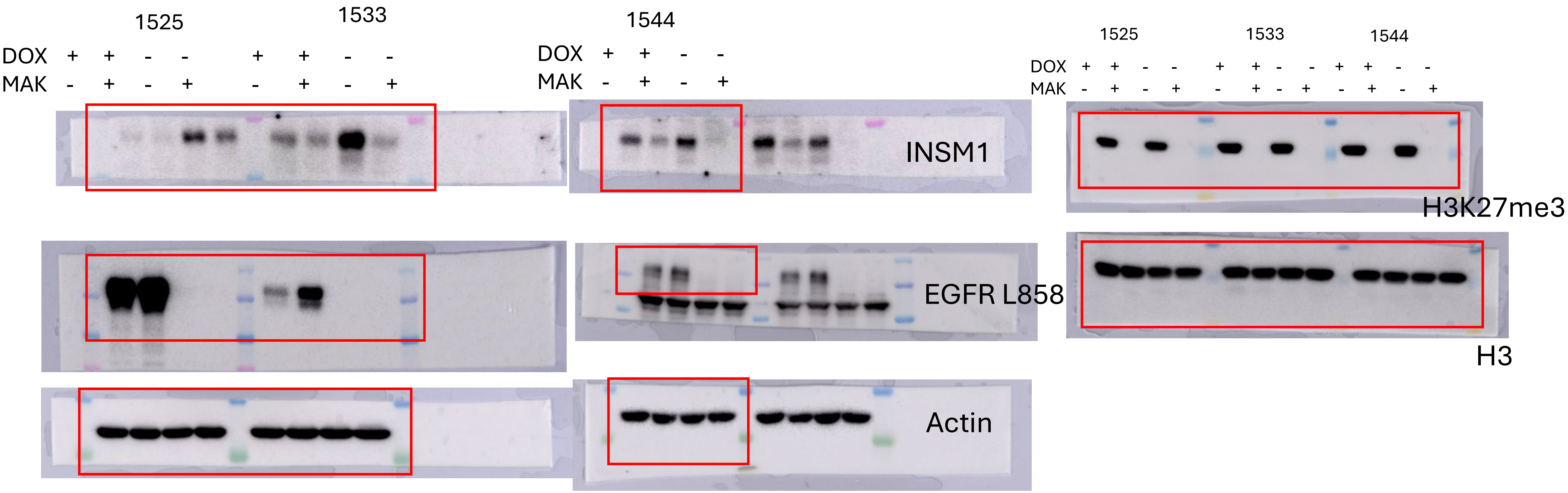

Full unedited immunoblots for Supplemental Figure 1A

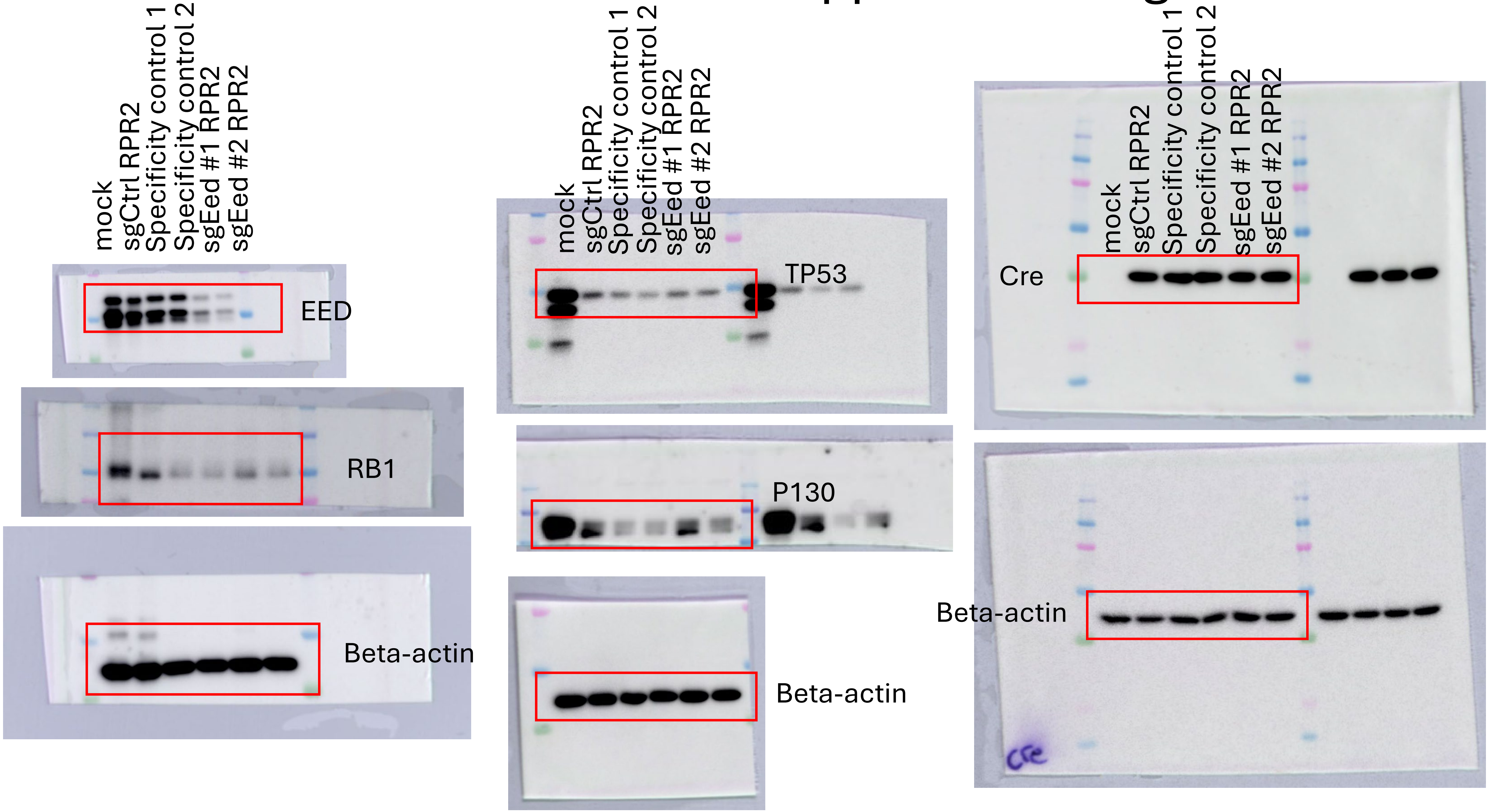

Full unedited immunoblots for Supplemental Figure 3J

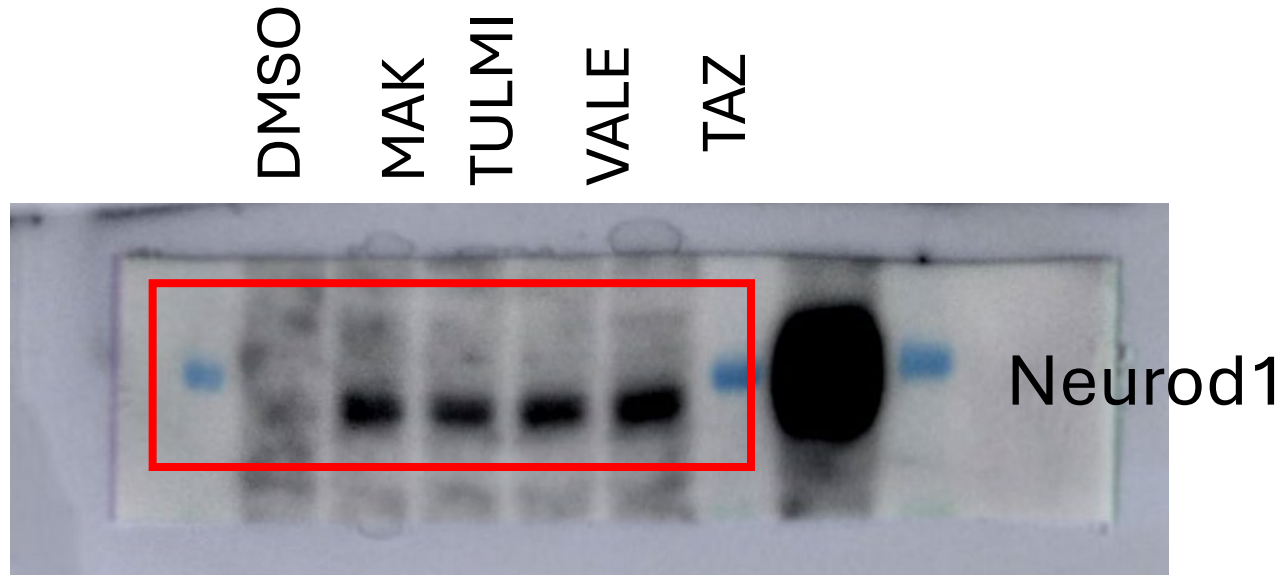

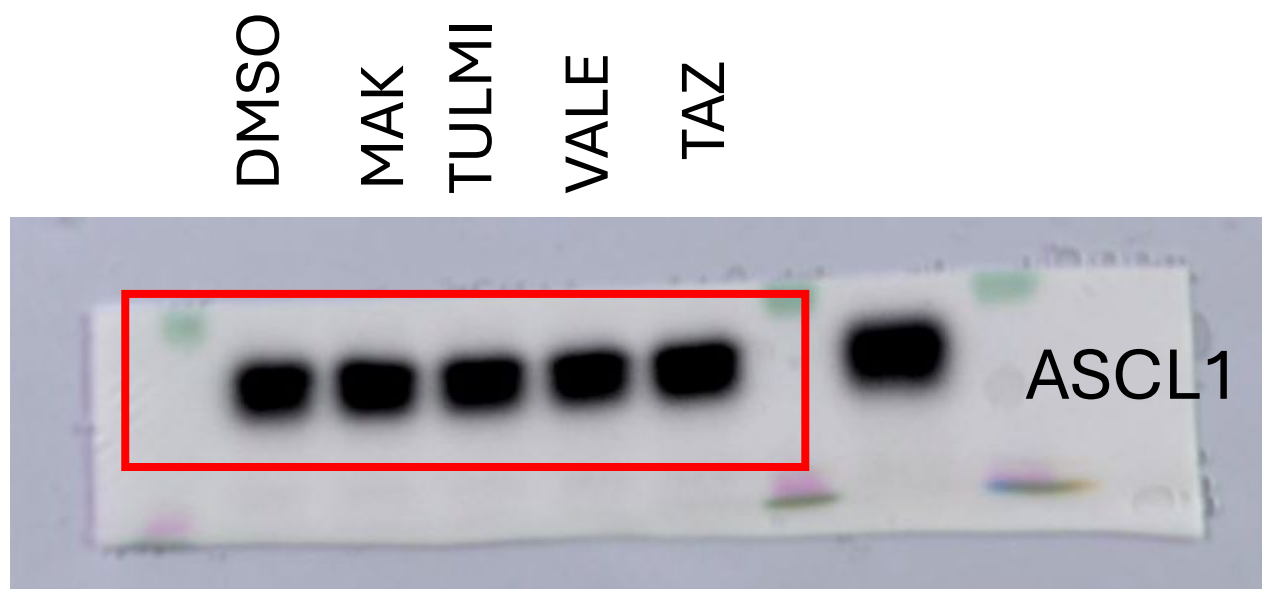

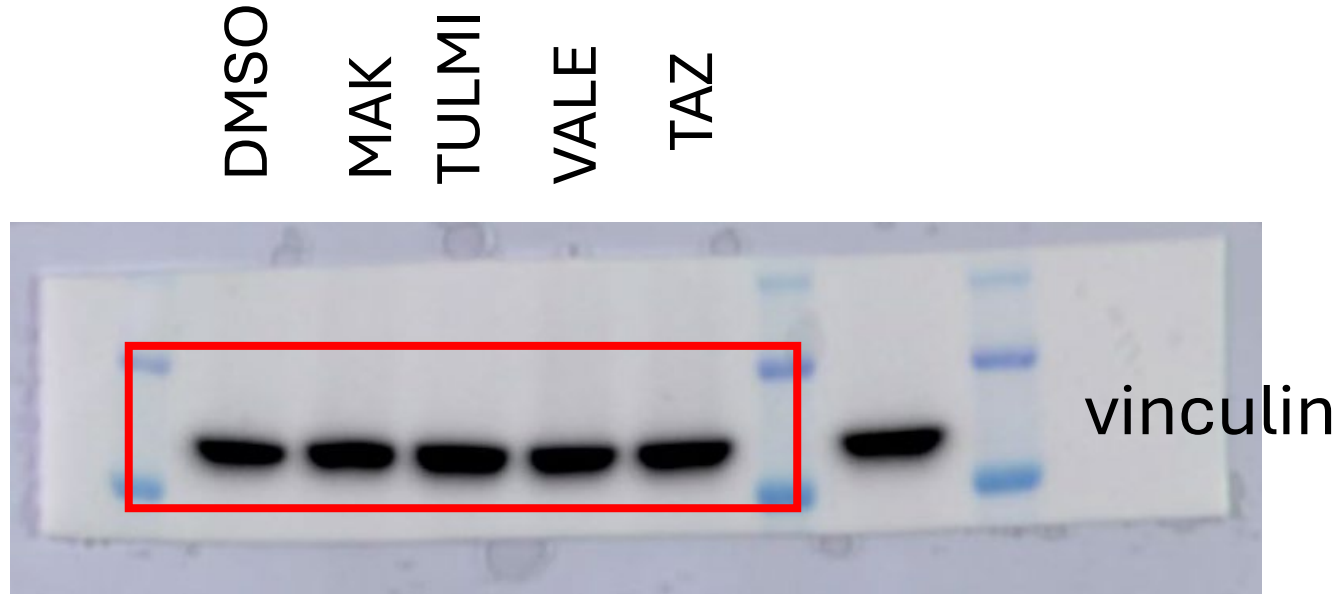

Full unedited immunoblots for Supplemental Figure 7C

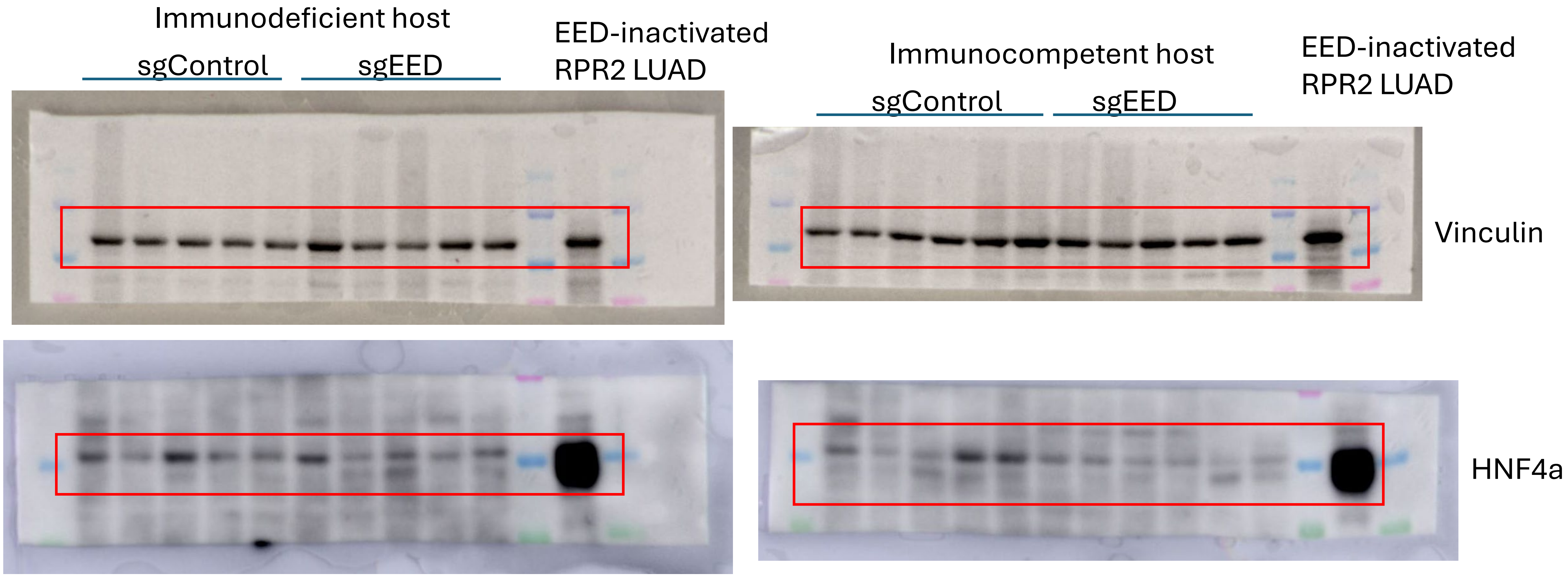

Full unedited immunoblots for Supplemental Figure 9B

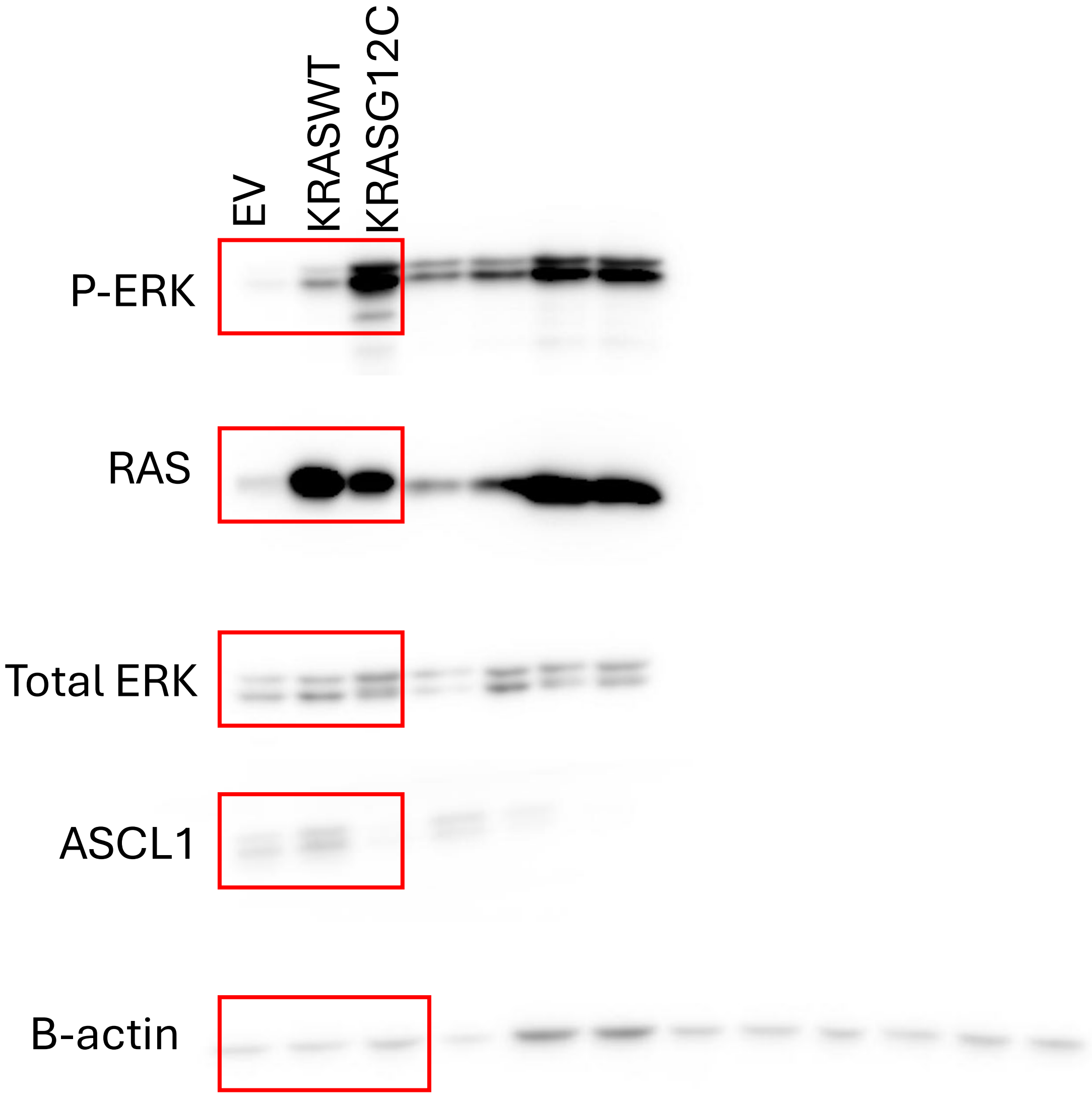

Full unedited immunoblots for Supplemental Figure 9E

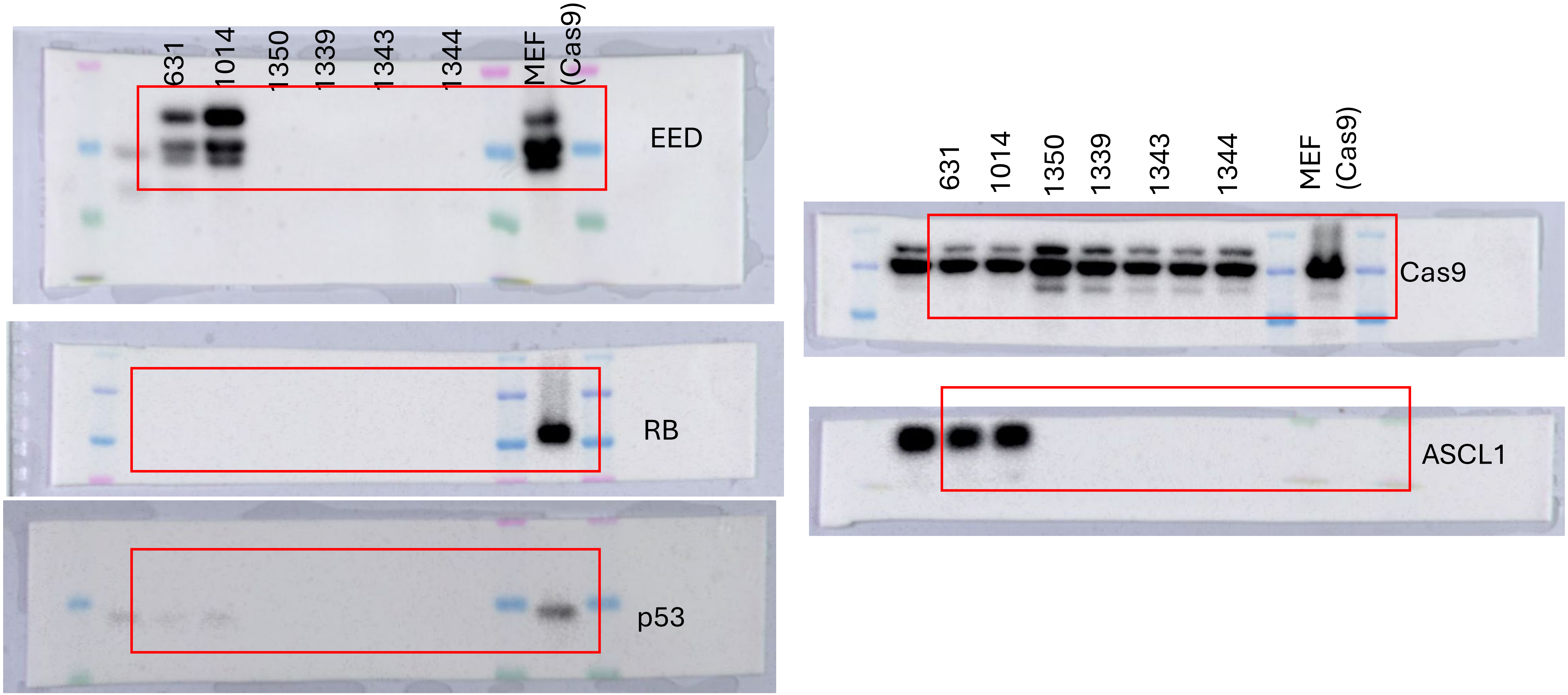

Full unedited immunoblots for Supplemental Figure 9F

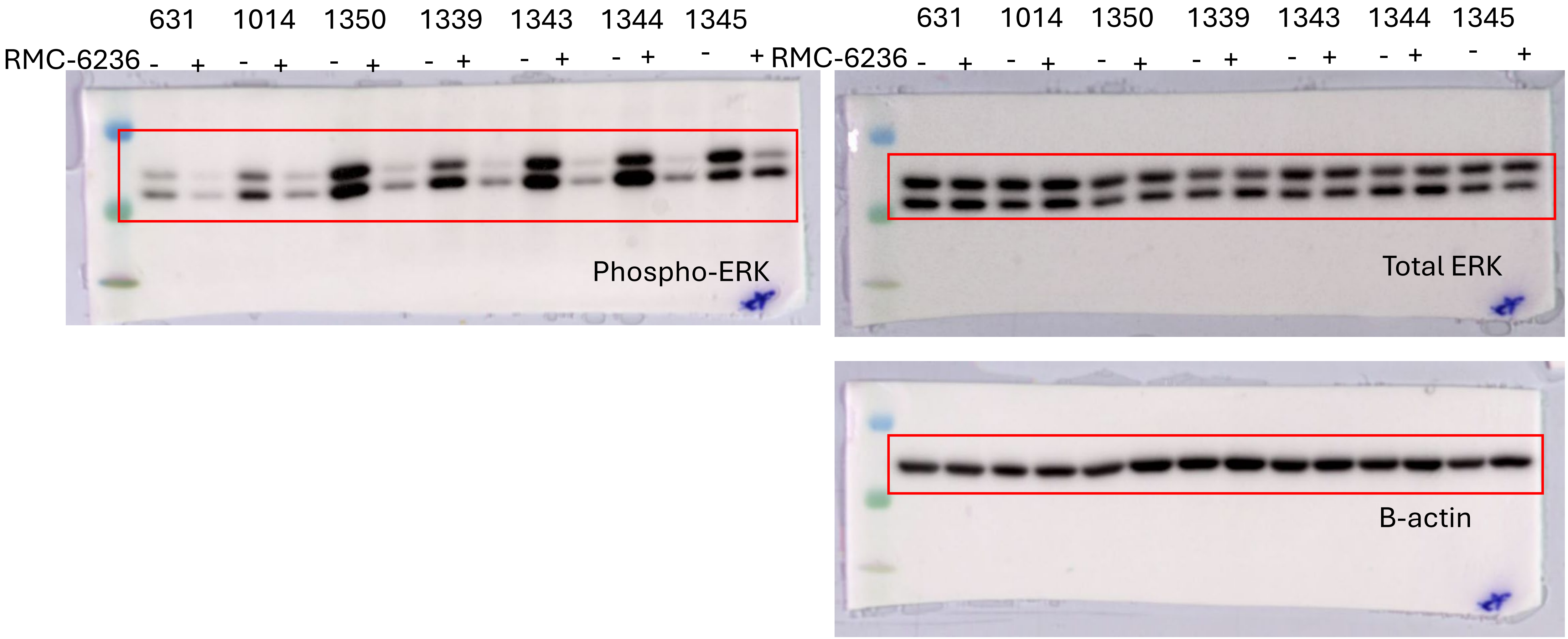

Full unedited immunoblots for Supplemental Figure 10A

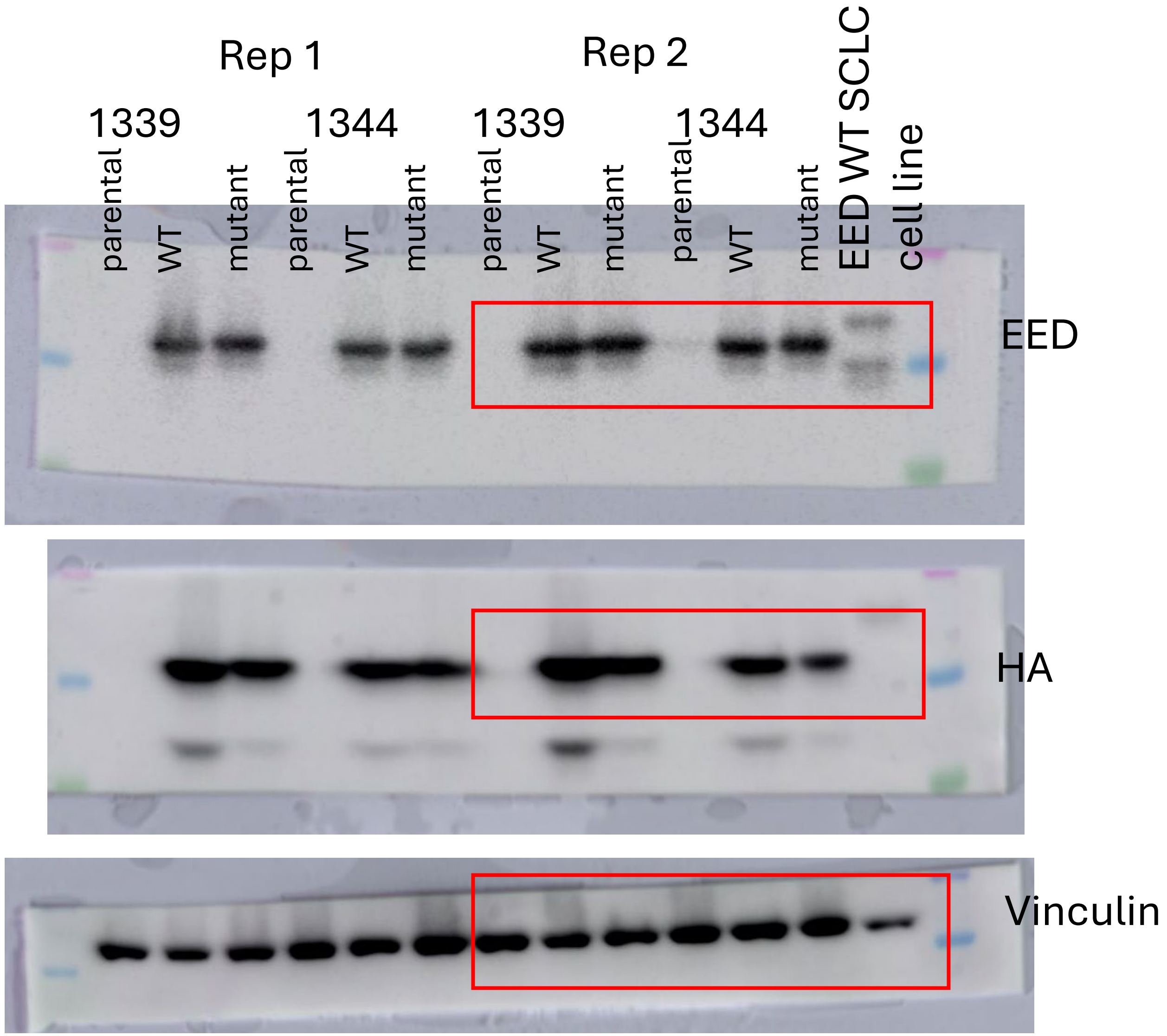

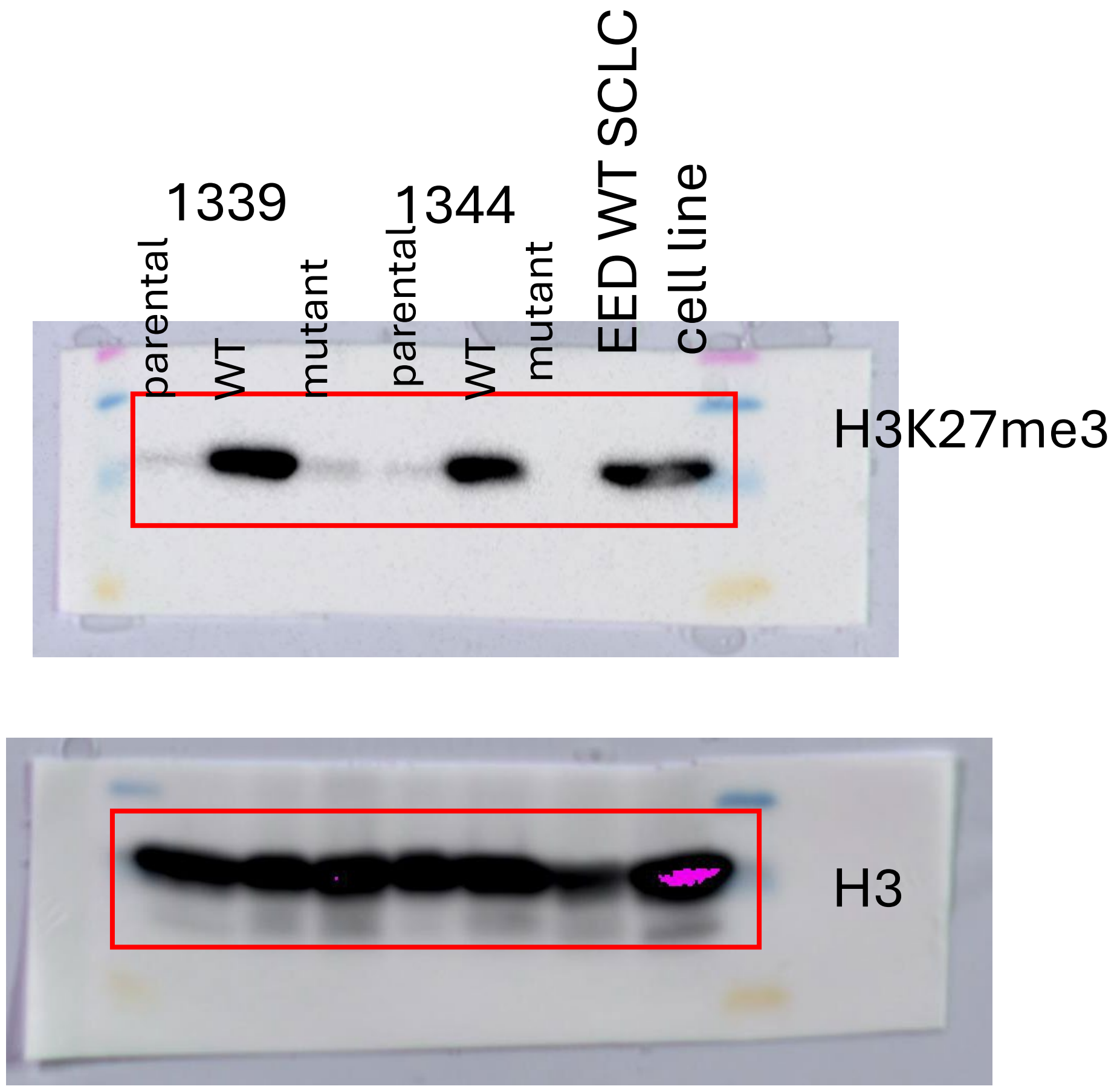

Full unedited immunoblots for Supplemental Figure 12A

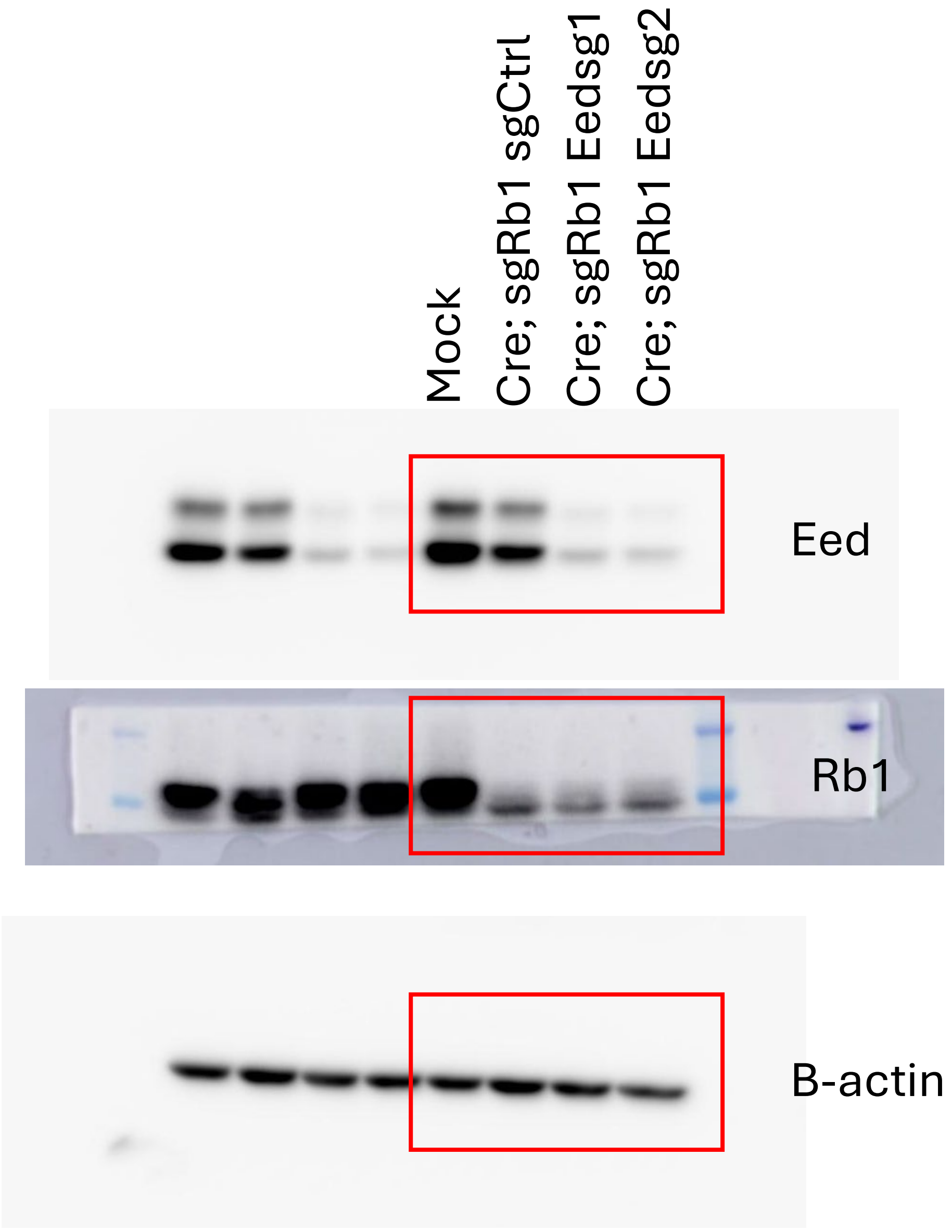

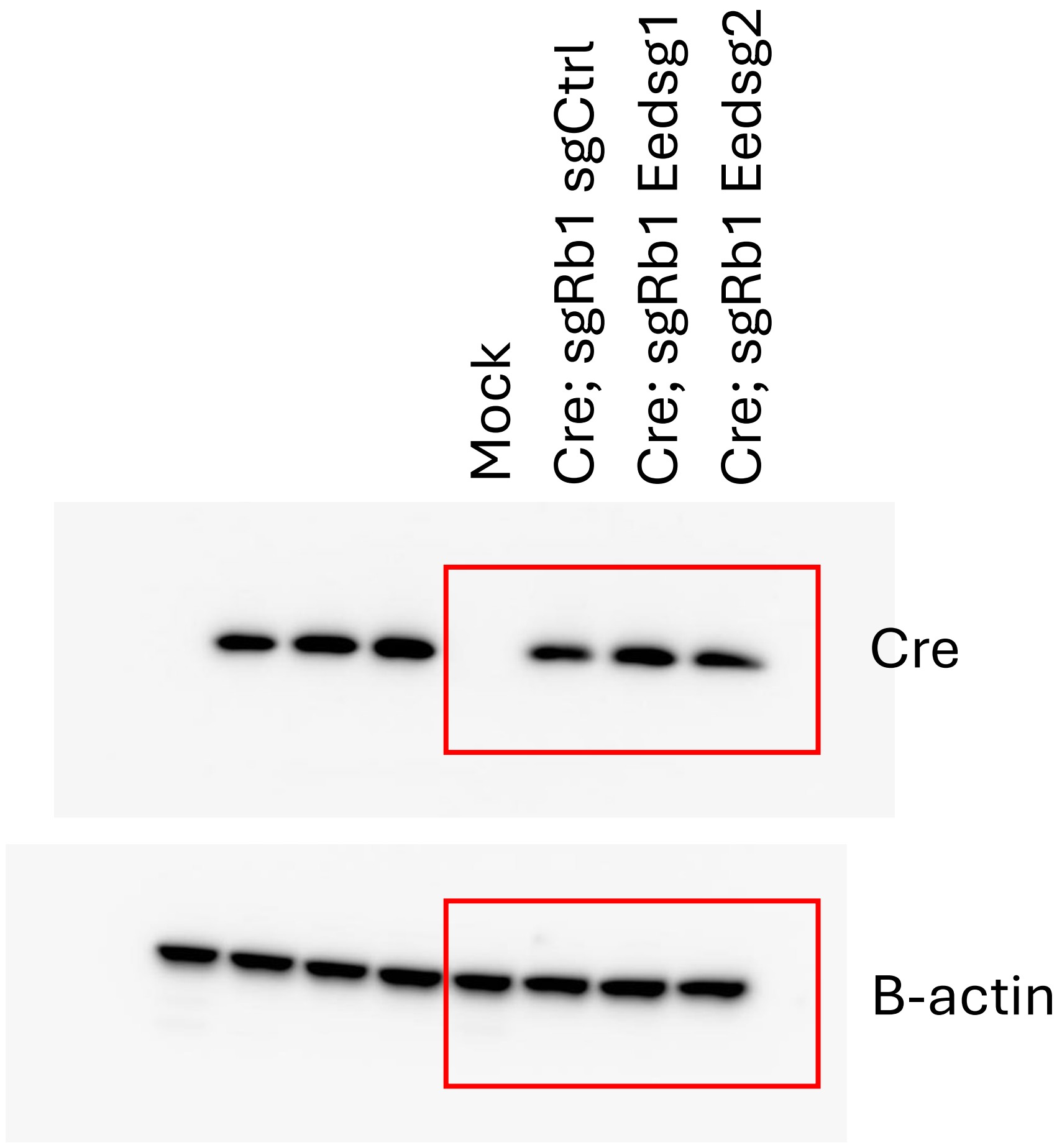

Full unedited immunoblots for Supplemental Figure 12C

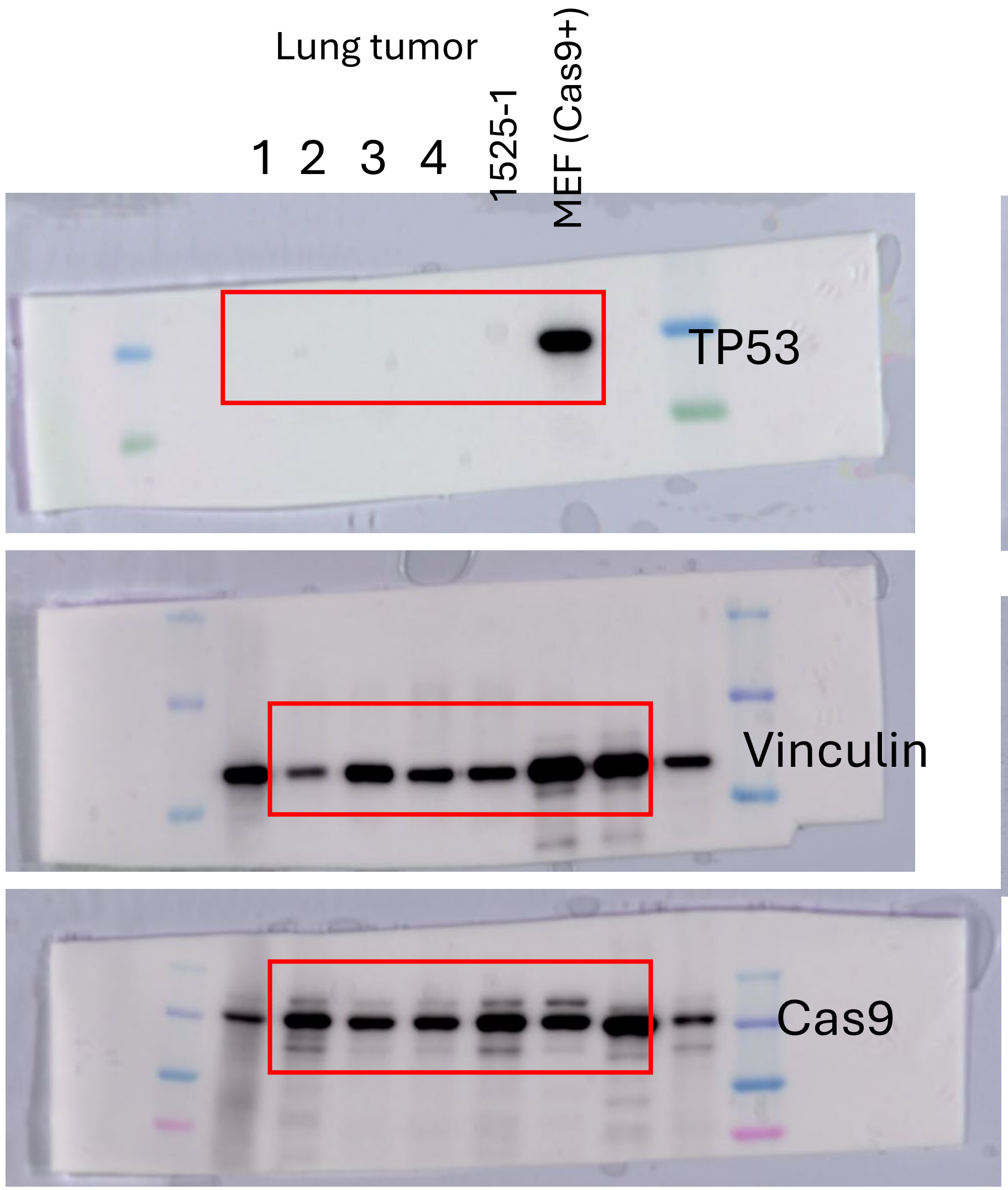

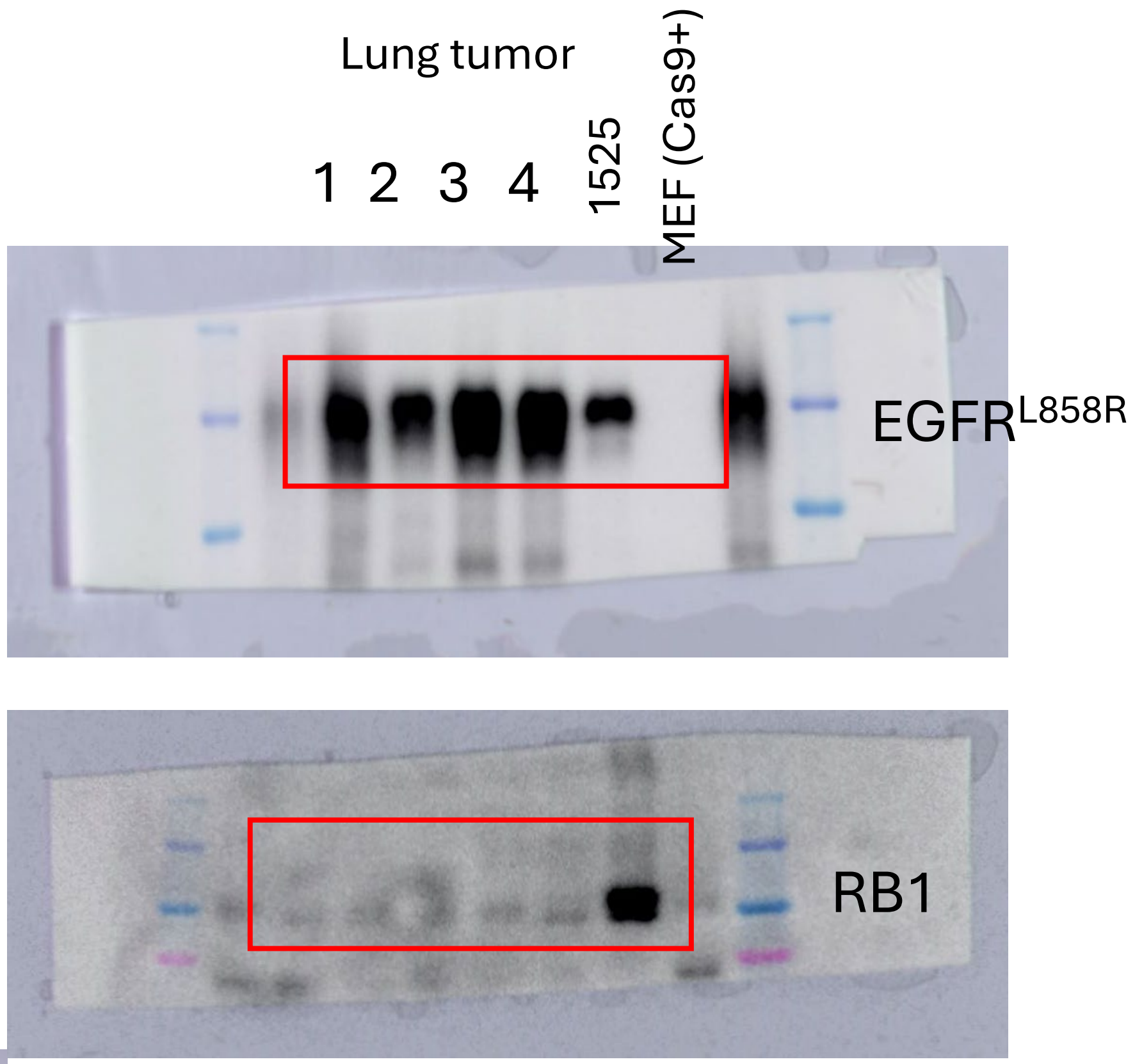

Full unedited immunoblots for Supplemental Figure 14E

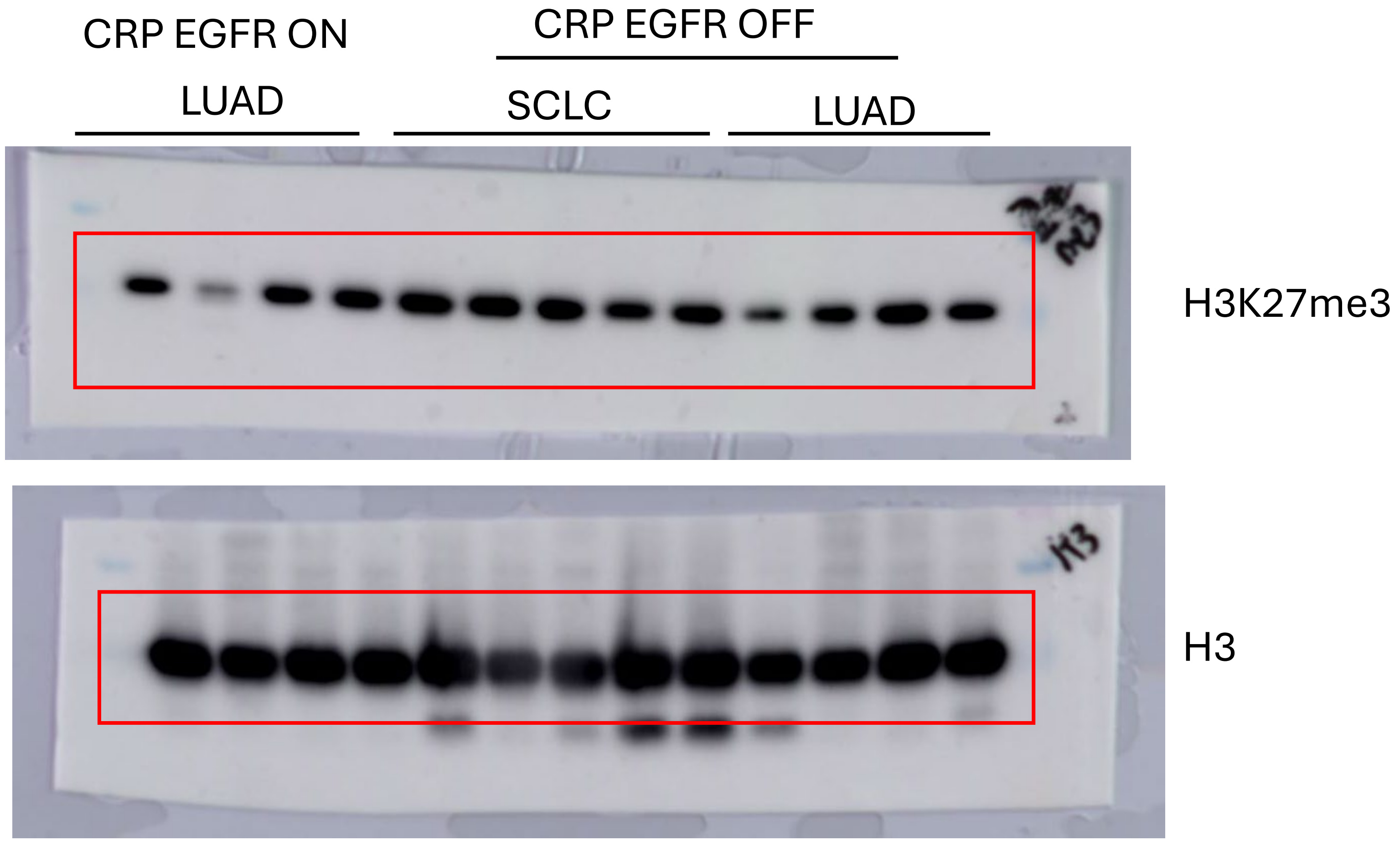

Supplement: Supplementary file 2 [file jci-136-199454-s044.pdf]
